# Nilotinib Effects on Safety, Tolerability, and Potential Biomarkers in Parkinson Disease: A Phase 2 Randomized Clinical Trial

**DOI:** 10.1001/jamaneurol.2019.4200

**Published:** 2019-12-16

**Authors:** Fernando. L. Pagan, Michaeline L. Hebron, Barbara Wilmarth, Yasar Torres-Yaghi, Abigail Lawler, Elizabeth E. Mundel, Nadia Yusuf, Nathan J. Starr, Muhammad Anjum, Joy Arellano, Helen H. Howard, Wangke Shi, Sanjana Mulki, Tarick Kurd-Misto, Sara Matar, Xiaoguang Liu, Jaeil Ahn, Charbel Moussa

**Affiliations:** 1 Translational Neurotherapeutics Program, Laboratory for Dementia and Parkinsonism, Department of Neurology, Georgetown University Medical Center, Washington, DC; 2 Movement Disorders Clinic, Department of Neurology, MedStar Georgetown University Hospital, Washington, DC; 3 Department of Biostatistics, Bioinformatics and Biomathematics, Georgetown University Medical Center, Washington, DC

## Abstract

**Importance:**

This study evaluated nilotinib safety and its effects on biomarkers as a potential disease-modifying drug in Parkinson disease.

**Objectives:**

To assess nilotinib effects on safety and pharmacokinetics and measure the change in exploratory biomarkers in patients with moderately severe Parkinson disease.

**Design, Setting, and Participants:**

This was a single-center, phase 2, randomized, double-blind, placebo-controlled trial with 300 patients approached in clinic; of these, 200 declined to participate, 100 were screened, 25 were excluded, and 75 were randomized 1:1:1 into placebo; nilotinib, 150-mg; or nilotinib, 300-mg groups. Recruitment started on May 17, 2017, and ended April 28, 2018, and follow-up ended August 10, 2019. Parkinson disease was confirmed according to the UK Brain Bank diagnostic criteria and symptoms were stabilized with use of optimal levodopa and/or dopamine agonists and other medications used in Parkinson disease.

**Interventions:**

Nilotinib vs placebo, administered orally once daily for 12 months followed by a 3-month washout period.

**Main Outcomes and Measures:**

It was hypothesized that nilotinib is safe and can be detected in the cerebrospinal fluid, where it alters exploratory biomarkers via inhibition of Abelson tyrosine kinase and potentially improves clinical outcomes.

**Results:**

Of the 75 patients included in the study, 55 were men (73.3%); mean (SD) age was 68.4 (8.2) years. Doses of 150 or 300 mg of nilotinib were reasonably safe, although more serious adverse events were detected in the nilotinib (150 mg: 6 [24%]; 300 mg: 12 [48%]) vs placebo (4 [16%]) groups. The 150-mg nilotinib group showed an increase in cerebrospinal fluid levels of the dopamine metabolites homovanillic acid (159.80nM; 90% CI, 7.04-312.60nM; *P* = .04) and 3,4-dihydroxyphenylacetic acid (4.87nM; 90% CI, 1.51-8.23nM; *P* = .01), and the 300-mg nilotinib group showed an increase in 3,4-dihydroxyphenylacetic acid (7.52nM; 90% CI, 2.35-12.69nM; *P* = .01). The nilotinib 150-mg but not the nilotinib 300-mg group demonstrated a reduction of α-synuclein oligomers (−0.04 pg/mL; 90% CI, −0.08 to 0.01 pg/mL; *P* = .03). A significant reduction of hyperphosphorylated tau levels was seen in the nilotinib 150-mg (−10.04 pg/mL; 90% CI, −17.41 to −2.67 pg/mL; *P* = .01) and nilotinib 300-mg (−12.05 pg/mL; 90% CI, −19.21 to −4.90 pg/mL; *P* = .01) groups.

**Conclusions and Relevance:**

In this study, nilotinib appeared to be reasonably safe and detectable in the cerebrospinal fluid. Exploratory biomarkers were altered in response to nilotinib. Taken together, these data will guide the development of a phase 3 study to investigate the effects of nilotinib therapy in patients with Parkinson disease.

**Trial Registration:**

ClinicalTrials.gov identifier: NCT02954978

## Key Points

**Question:**

Is the use of nilotinib hydrochloride, a drug approved for use in leukemia, safe and effective in patients with Parkinson disease?

**Findings:**

In this randomized clinical trial of use of nilotinib hydrochloride in 75 patients with Parkinson disease, doses of 150 or 300 mg were reasonably safe and did not inhibit plasma Abelson tyrosine kinase. Twelve months of treatment with nilotinib altered exploratory cerebrospinal fluid biomarkers, including brain dopamine turnover, oligomeric α-synuclein, and hyperphosphorylated tau.

**Meaning:**

This phase 2 trial met its objectives and nilotinib should be investigated in a phase 3 study as a potential disease-modifying therapy in Parkinson disease.

## Teaser

This randomized clinical trial evaluates the efficacy and safety of nilotinib hydrochloride for use in treatment of patients with Parkinson disease.

## Introduction

Parkinson disease (PD) is the second most common neurodegenerative disorder worldwide, causing motor and nonmotor symptoms, loss of dopamine, and accumulation of misfolded α-synuclein.^1,2,3^ Nilotinib hydrochloride is a multikinase inhibitor that preferentially targets Abelson (Abl)^4,5,6^ and discoidin domain receptors^4,5,6,7^ and effectively reduces misfolded proteins in several models of neurodegeneration.^7^ Nilotinib is approved in the United States for treatment of Philadelphia chromosome‒positive chronic myeloid leukemia at oral dosages of 300 mg twice daily.^4,5,6^ Several studies reported that low doses of nilotinib enter the brain and degrade α-synuclein and tau in animal models of neurodegeneration.^7,8,9,10,11^ A previous study suggested that nilotinib may increase dopamine metabolism and potentially treat motor and nonmotor symptoms of PD.^12^

The primary objective of this study was to evaluate nilotinib safety, tolerability, and pharmacokinetics in patients with moderately severe PD. A secondary objective was to assess nilotinib effects on exploratory cerebrospinal fluid (CSF) biomarkers, including dopamine metabolites homovanillic acid (HVA) and 3,4-dihydroxyphenylacetic acid (DOPAC), as well as α-synuclein and tau. An exploratory objective was to assess nilotinib effects on motor and nonmotor symptoms of PD at baseline, 6, 12, and 15 months.

## Methods

### Participants

All participants were confirmed to have PD according to the UK Brain Bank diagnostic criteria, with Hoehn and Yahr stage 2.5-3.0 (range indicating moderate severity), Montreal Cognitive Assessment score 22 or higher (mild cognitive impairment), and Movement Disorders Society–Unified Parkinson’s Disease Rating Scale (MDS-UPDRS)-III motor score 20 to 40 (moderate severity per clinician-scored monitored motor evaluation). Other parts of the MDS-UPDRS were used in evaluation of secondary outcomes (I: evaluation of mentation, behavior, and mood; II: self-evaluation of the activities of daily life, including speech, swallowing, handwriting, dressing, hygiene, salivating, turning in bed, walking, and cutting food; IV: complications of therapy).

This was a single-center study that was conducted by the Translational Neurotherapeutics Program at Georgetown University Medical Center, Washington, DC. Recruitment started May 17, 2017, and ended April 28, 2018, and follow-up ended August 10, 2019. This study was conducted in accordance with Good Clinical Practice guidelines and the Declaration of Helsinki,^13^ and was approved by the Georgetown University Medical Center Institutional Review Board. The protocol is available in Supplement 1. All participants provided written informed consent, and their treatment was optimized with current PD medications, including levodopa, 800 mg/d, or less, and levodopa and/or dopamine agonists approximately 1 to 2 months before consenting and undergoing screening. Participants did not receive financial compensation (eMethods in Supplement 2).

### Randomization and Blinding

This study used a block randomization using the blockrand function in R software, version 3.4 (R Foundation) to randomize 75 participants into the 3 treatment groups. The block size varied between 6 and 12 and the randomization was done within blocks to ensure a balance in sample sizes across group blocks.^14^ All site staff, investigators, raters, participants, and caregivers were blinded to dose and treatment. Medications were labeled at the Georgetown University Medical Center Clinical Research Unit with a package medical identification number. Each participant was assigned a specific identification number, which was noted by the investigator on the designated medication package after randomization.

### Statistical Analysis

Baseline descriptive statistics, such as mean (SD) for continuous demographic and dose variables and frequencies of safety end points, were summarized for the 3 treatment groups. The proportions of serious adverse events (SAEs) and nonserious AEs among the 3 groups were compared using Pearson χ^2^ tests. The changes in exploratory biomarkers within each group were compared using paired *t* tests with Welch corrections. Exploratory clinical end points in the 3 groups at baseline, 6 months, 12 months, and 15 months were summarized using sample means (SDs). For each treatment group, a paired Wilcoxon signed rank test was used to test whether changes occurred in each clinical end point between baseline and 6 months, baseline and 12 months, 6 and 12 months, baseline and 15 months, and 12 and 15 months. Trajectories of changes in all clinical end points over visits were plotted over time with means (SDs). Changes among the 3 treatment groups were evaluated using analysis of variance. For each clinical outcome, a linear mixed-effects model was fitted using the treatment group, categorical time, and their interactions as independent variables. For exploratory biomarker end point comparisons, 1-sided, type I error of 5% and 90% CI were used, and 2-sided, type I error of 5% and 95% CI for clinical end point comparisons were used. We adjusted multiple testing using false-discovery rate (FDR), (p*, *P* value after analysis for multiple analysis using FDR) when multiplicity existed. All statistical analyses were performed using R, version 3.40. *P* ≤ .05 was considered significant or, as indicated in the eTables in Supplement 2, following FDR analysis.

## Results

### Patients, Demographics, Enrollment, and Randomization

**Figure 1.**
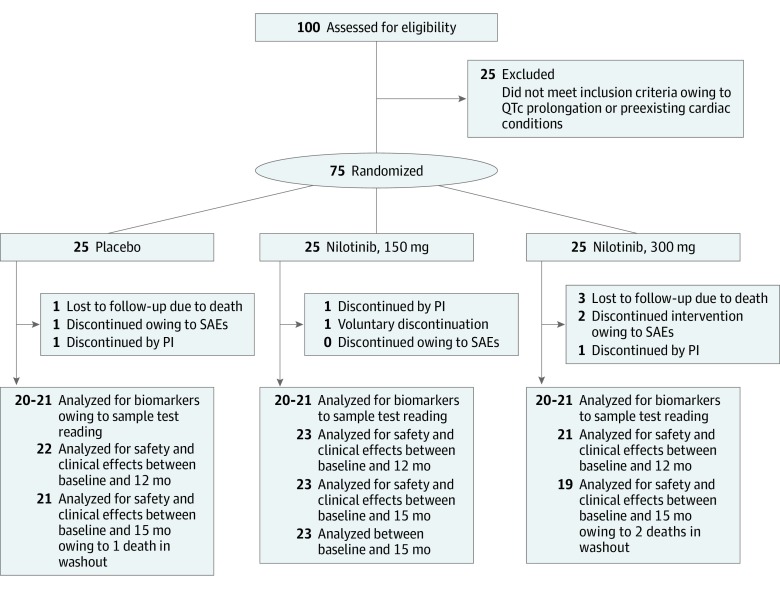
CONSORT Flow Diagram Phase 2, randomized, double-blind, placebo-controlled trial to evaluate nilotinib hydrochloride effects on safety, tolerability, biomarkers, and clinical outcomes in Parkinson disease. For values given as 20-21 in the bottom boxes, biomarkers were analyzed and only those samples who gave a reading were reported. A few samples did not produce a value when analyzed. PI indicates principal investigator; SAE, serious adverse event.

A total of 300 patients were approached in the clinic, 200 declined to participate, and 100 potential participants were screened; of these, 25 did not meet the inclusion criteria due to QTc interval prolongation and 75 were enrolled (Figure 1 and Table 1). Mean (SD) age of the participants was 68.4 (8.2) years and the sample included 20 women (26.7%) and 55 men (73.3%). A total of 66 participants (88.0%) completed the treatment and there were no dropouts due to lack of drug tolerability. The mean (SD) dose of levodopa at enrollment was 606.63 (258.65) mg/d in the placebo group, 602.04 (204.56) mg/d in the nilotinib 150-mg group, and 612.67 (286.63) mg/d in the nilotinib 300-mg group, and these values were less at the end of treatment due to dropouts from each group (Table 2). Some participants with shorter disease duration or those with Hoehn and Yahr stage 2.5 were reluctant to use levodopa and their PD symptoms were managed by dopamine agonists and other PD medications (eTable 8 in Supplement 2).

**Table 1.** Demographics and Enrollment Summary

Characteristic	Placebo	Nilotinib, 150 mg	Nilotinib, 300 mg
Total, No.			
Enrolled	25	25	25
Finished end of treatment, No. (%)	23 (92)	22 (88)	21 (84)
Dropped out during washout period, No. (%)	1 (4)	0	2 (8)
Age, mean (SD), y	68.64 (7.56)	66.56 (9.89)	70 (7.15)
Height, mean (SD), cm	175.07 (8.72)	169.15 (8.17)	173 (10.16)
Weight, mean (SD), kg	82.90 (15.4)	74.912 (14.59)	76.8 (14.45)
BMI	26.95 (4.00)	26.44 (4.81)	25.55 (3.96)
Sex, No. (%)			
Men	21 (84)	14 (56)	20 (80)
Women	4 (16)	11 (44)	5 (20)
Race, No. (%)			
White	24 (96)	25 (100)	25 (100)
Asian	1 (4)	0	0
Levodopa dose mean (SD), mg/d			
Baseline	606.63 (258.65)	602.04 (204.56)	612.67 (286.63)
12 mo	496.17 (323)	567 (310)	518 (259)
MDS-UPDRS-III score at baseline, mean (SD)^a^	28.92 (4.77)	28.52 (6.01)	30.8 (4.95)
Duration of disease, mean (SD), y	9.96 (5.23)	12.28 (4.98)	9.96 (6.54)
Hoehn and Yahr stage, range^b^	2.5-3.0	2.5-3.0	2.5-3.0

Abbreviations: BMI, body mass index (calculated as weight in kilograms divided by height in meters squared); MDS-UPDRS, Movement Disorders Society–Unified Parkinson’s Disease Rating Scale.

^a^ Scores of 20 to 40 indicate moderate severity per clinician-scored monitored motor evaluation.

^b^ Moderate severity.

**Table 2.** Summary of All Standard, Nonserious Adverse Events

Systems/Preferred Organ	Events, No. (%)		
	Placebo (n = 25)	Nilotinib 150 mg (n = 25)	Nilotinib 300 mg (n = 25)
Cardiac disorders: QTc interval prolongation, palpitations, bigeminy, pacemaker adjustment	0	QTc interval prolongation: 2 (8); bigeminy: 1 (4); hypertension: 1 (4)	Pacemaker: 1 (4); whooshing in chest: 1 (4)
Eye disorders: stye, cataract, laceration, blurry vision	Stye: 2 (8); cataract: 1 (4); laceration: 2 (8)	Stye: 2 (8)	Blurry vision: 1 (4)
Gastrointestinal disorders: virus, nausea, constipation, diarrhea, dry heaves, abdominal pain, colitis, hemorrhoids	Stomach virus: 5 (20)	Constipation: 1 (4); nausea: 1 (4); diarrhea: 1 (4); virus: 1 (4)	Abdominal pain: 4 (16); hemorrhoids: 1 (4)
General and hematologic disorders: serum metabolites, anemia, falls, flu, tinnitus	Falls: 9 (36); flu: 2 (8); hematologic: 1 (4); sinusitis: 2 (8); tinnitus: 1 (4)	Falls: 13 (52); hematologic: 1 (4); sinusitis: 2 (8)	Falls: 13 (52); flu: 3 (12); hematologic: 1 (4); sinusitis: 3 (12)
Hepatic and pancreatic disorders: elevated lipase/amylase levels	0	1 (4)	2 (8)
Musculoskeletal/connective tissue: mild pain, dental, sciatica, bursitis, abscess, arthritis, disk herniation	Pain: 10 (40)	Pain: 16 (64)	Pain: 9 (36)
Nervous system disorders: headache, mild confusion, vertigo, amnesia	Vertigo: 1 (4); confusion: 1 (4); post LP headache: 5 (20)	Vertigo: 1 (4); amnesia: 1 (4); post LP headache: 5 (20)	Post LP headache: 1 (4)
Psychiatric disorders: mild hallucinations	Hallucinations: 1 (4)	0	0
Renal and urinary disorders: hematuria, UTI, decreased glomerular filtration rate, prostate infection	Hematuria: 2 (8)	UTI: 1 (4); glomerular filtration rate: 1 (4); prostate infection: 1 (4)	UTI: 2 (8)
Respiratory, thoracic, and mediastinal disorders: mild cough, pneumonia, asthma, dyspnea, bronchitis, URI	Cough: 2 (8); pneumonia: 2 (8); URI: 5 (20); bronchitis: 2 (8)	URI: 6 (27); pneumonia: 1 (4); dyspnea: 1 (4); asthma: 1 (4)	URI: 5 (20); bronchitis: 2 (8)
Skin and subcutaneous disorder: moderate itching, skin lesions, melanoma, cysts, rash, edema	Itching and rash: 3 (12); melanoma excision: 1 (4); skin lesions: 3 (12); edema: 1 (4); cyst: 1 (4)	Itching and rash: 3 (12); wart lesion: 1 (4); skin biopsies: 3 (12); testicular nodule: 1 (4); cyst: 1 (4)	Nonhealing wound: 1 (4); melanoma excision: 1 (4); skin lesions: 3 (12); edema: 1 (4); cyst: 1 (4); tick bites: 1 (4)

Abbreviations: LP, lumbar puncture; URI, upper respiratory tract infection; UTI, urinary tract infection.

### Nonserious AEs

The total number of AEs was 65 in the placebo, 71 in the nilotinib 150-mg, and 57 in the nilotinib 300-mg (Table 2) groups, but there was no significant difference (*P* = .08) in AEs between the 150-mg and 300-mg nilotinib groups compared with the placebo group. Falls were the most common AEs, but there was no significant difference between the nilotinib and placebo groups (placebo: 9 [36%]; nilotinib 150 mg: 13 [52%]; nilotinib 300 mg: 13 [52%]; *P* = .42). Other common AEs in all groups were musculoskeletal (placebo: 10 [40%]; nilotinib 150 mg: 16 [64%]; nilotinib 300 mg: 9 [36%]), respiratory (placebo: 11 [44%]; nilotinib 150 mg: 9 [36%]; nilotinib 300 mg: 7 [28%]), skin and subcutaneous disorders (placebo: 9 [36%]; nilotinib 150 mg: 9 [36%]; nilotinib 300 mg: 8 [32%]). Gastrointestinal (placebo: 5 [20%]; nilotinib 150 mg: 4 [16%]; nilotinib 300 mg: 5 [20%]) and cardiac (placebo: 0; nilotinib 150 mg: 4 [16%]; nilotinib 300 mg: 2 [8%]) symptoms were less common. Hematologic disorders, flulike symptoms, sinus infection, eye disorders, and hearing AEs were rare. Transient but rare elevation of pancreatic enzymes was seen in the nilotinib 150-mg (1 [4%]) and 300-mg (2 [8%]) groups. Hallucinations were reported in the placebo group (1 [4%]) and headache after lumbar puncture was rare in all groups (placebo: 5 [20%], nilotinib 150 mg: 5 [20%], nilotinib 300 mg: 1 [4%]). Central nervous system disorders, including vertigo, confusion, and amnesia were rarely reported. Urinary disorders were rarely observed in all groups.

### Serious Adverse Events

The total number of SAEs was 4 in the placebo group (16%), 6 in the nilotinib 150-mg group (24%), and 12 in the nilotinib 300-mg group (48%) (Table 3), which was a significant difference (*P* = .03) in total SAEs for the 3 treatment groups. Post hoc comparisons showed that there was a significant difference between the placebo and 300-mg nilotinib groups (*P* = .02) but not between the 150- and 300-mg nilotinib groups (*P* = .08). Four cardiovascular SAEs were observed. In the placebo group, 1 participant was hospitalized for palpitations and completed the study, and another participant had a stroke and was withdrawn from the study. In the nilotinib 150-mg group, 1 participant experienced QTc interval prolongation and a surge of catecholamine due to sildenafil citrate, mimicking Takotsubo cardiomyopathy. This individual did not disclose a similar episode and use of sildenafil before enrollment and was withdrawn. In the nilotinib 300-mg group, 1 participant had angina-type symptoms and underwent 1 stent replacement, but this participant completed the study. Another participant had a history of hypertension and atrial flutter post ablation but showed no symptoms or QTc interval abnormalities during screening and enrollment. Non-ST segment elevation myocardial infraction was confirmed and this participant was withdrawn from the study. There was no significant difference (*P* = .81) in cardiovascular SAEs between all 3 groups and no QTc interval prolongation (other than in the patient with Takotsubo cardiomyopathy) was seen in any of the groups (eTable 1, eTable 2, eTable 3 in Supplement 2).

**Table 3.** Summary of All SAEs Reported

Systems/Preferred Organs	Events, No. (%)		
	Placebo (n = 25)	Nilotinib 150 mg (n = 25)	Nilotinib 300 mg (n = 25)
Total SAEs, No. (%)	4 (16)	6 (24)	12 (48)
Patients, No. (%)	3 (12)	5 (20)	9 (36)
Cardiac disorders: QTc interval prolongation, palpitations, angina, stenting	Palpitations: 1 (4)	Prolonged QTc interval due to Takotsubo cardiomyopathy: 1 (4)	Angina type symptoms and stent placement, returned to study and completed: 1 (4); atrial flutter detected on ECG followed by 2 stent replacements, history of hypertension and atrial flutter status post ablation: 1 (4)
Renal and urinary tract: urinary tract infection			Urinary tract infection: 1 (4)
Gastrointestinal disorders: cancer			Death due to metastatic pancreatic cancer: 1 (4)
General disorders: fall, cramp/pain		Fall: 1 (4)	Severe cramp pain and fall: 1 (4)
Musculoskeletal and connective tissue disorders: hip fracture/prosthetic: discitis/osteomyelitis	Right hip fracture: 1 (4); discitis osteomyelitis: 1 (4)	Left hip fracture: 1 (4); Prosthetic repair: 1 (4)	Hip fracture: 1 (4)
Nervous system disorders: stroke, orthostatic hypotension	Stroke: 1 (4)	0	Orthostatic hypotension: 1 (4)
Psychiatric disorders: hallucinations, suicidal ideation	0	0	Psychosis and attempted suicide: 1 (4); hallucinations: 1 (4)
Respiratory, thoracic, and mediastinal disorders: pneumonia, bronchitis	0	Aspiration pneumonia: 1 (4)	Bronchial disorder: 1 (4); pulmonary embolism: 1 (4)
Skin and subcutaneous disorder: cellulitis	0	1 (4)	1 (4)
Death in washout	Death: 1 (4) due to disease progression	0	Death: 1 (4) due to drowning; 1 (4) due to pneumonia

Abbreviations: ECG, electrocardiogram; SAEs, serious adverse events.

In the placebo group, participants were hospitalized for discitis osteomyelitis (1 [4%]) and hip fracture (1 [4%]). In the nilotinib 150-mg group, participants were hospitalized for fall (1 [4%]), hip fracture (1 [4%]), prosthetic repair (1 [4%]), aspiration pneumonia (1 [4%]), orthostasis, and cellulitis (1 [4%]). In the nilotinib 300-mg group, participants were hospitalized for urinary tract infection (1 [4%]), bronchitis (1 [4%]), hip fracture (1 [4%]), fall (1 [4%]), and cellulitis (1 [4%]). One participant (4%) had metastatic pancreatic cancer and 3 weeks later, this participant developed a pulmonary embolism after biopsy and died in hospice. One participant (4%) was hospitalized for psychosis and suicidal ideation and 2 weeks later attempted suicide. The psychiatrist and family members confirmed the same suicidal ideation and psychotic events before enrollment and this participant was withdrawn for nonadherence to his medications for 3 weeks before the SAE. Another participant (4%) was hospitalized due to hallucinations but completed the study.

During the washout period, 1 participant (4%) in the placebo group died while undergoing a computed tomographic scan due to apparent disease progression. In the nilotinib 300-mg group, 1 participant (4%) died due to unintentional drowning and another participant (4%) died in an acute rehabilitation facility after hospitalization for pneumonia.

### Nilotinib Effects on Biomarkers

**Figure 2.**
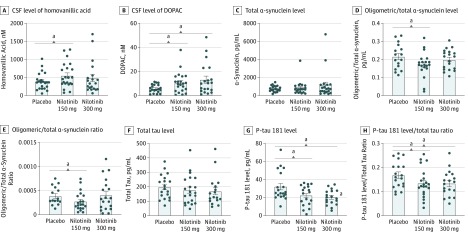
Data Representing the Pharmacokinetics and Pharmacodynamics Effects of 1-Year Nilotinib Treatment Compared With Placebo in Parkinson Disease Cerebrospinal fluid (CSF) levels of homovanillic acid HVA (A), 3,4-dihydroxyphenylacetic acid (DOPAC) (B), total α-synuclein (C), oligomeric α-synuclein (D), ratio of oligomeric/total α-synuclein (E), total tau (F), hyperphosphorylated tau (P-tau181) (G), and ratio of p-tau181/total tau (H). Total of 20 to 21 patients per group. ^a^*P* < .05.

Small amounts of nilotinib were detected in the CSF in the nilotinib 150-mg (0.94nM) and 300-mg (1.6nM) groups (eTable 9 in Supplement 2) but the drug was not found in the placebo group. Nilotinib was detected in the plasma in the 150-mg (245.2nM) and 300-mg (299.5nM) groups. Nilotinib, 150 mg, significantly increased CSF HVA levels (159.80nM; 90% CI, 7.04-312.60nM; *P* = .04) compared with placebo at 12 months (Figure 2A; eTable 4 in Supplement 2) and HVA levels nonsignificantly increased (86.64nM; 90% CI, −104.6 to 277.9nM) in the nilotinib 300-mg group. The CSF level of DOPAC was significantly increased at 12 months in the nilotinib 150-mg (4.87nM; 90% CI, 1.51-8.23nM) and the 300-mg (7.52nM; 90% CI, 2.35-12.69) groups (*P* = .01 for both groups) (Figure 2B; eTable 4 in Supplement 2) compared with placebo. Plasma DOPAC level was significantly increased at 12 months in the nilotinib 150-mg (187.80nM; 90% CI, 48.84-326.70nM) and 300-mg (284.50nM; 90% CI, 48.24-448.70nM) groups compared with placebo (eFigure 1 in Supplement 2). However, with FDR adjustment, plasma DOPAC, but not CSF DOPAC or HVA level, was significantly increased in the nilotinib 150-mg group (*P* = .05). Comparisons between the nilotinib and placebo groups at 12 months are presented herein, because participants were randomized after receiving a single random dose of nilotinib, 150, 200, 300, and 400 mg, vs placebo to perform population-based pharmacokinetics and pharmacodynamics at baseline as previously reported.^15^

No mean differences in total CSF α-synuclein levels were observed between the nilotinib and placebo groups at 12 months (−0.04 pg/mL; 90% CI, −0.08 to 0.01 pg/mL; *P* = .03) (Figure 2C). The ratio of CSF oligomeric/total α-synuclein was also reduced in the nilotinib 150-mg group (33%; 95% CI, 0.0001%-0.0003%) compared with placebo (Figure 2E; eTable 4 in Supplement 2). With FDR adjustment, there was no significant difference in α-synuclein between groups. Furthermore, no significant change was observed in total CSF tau between all study groups (Figure 2F). There were significant decreases in hyperphosphorylated tau (P-tau 181) in the nilotinib 150-mg (−10.04 pg/mL; 90% CI, −17.41 to −2.67 pg/mL; *P* = .01) and 300-mg (−12.05 pg/mL; 90% CI, −19.21 to −4.90 pg/mL; *P* = .01) groups compared with the placebo group (Figure 2G; eTable 4 in Supplement 2). There were significant decreases in the ratio of CSF P-tau 181/total tau (Figure 2H; eTable 4 in Supplement 2) in the nilotinib 150-mg (−3.30%; 90% CI, −6.40% to −0.30%) and 300-mg (−3.40%; 90% CI, −6.40% to −0.40%) groups compared with the placebo group (eTable 4 in Supplement 2). Only the p-tau level in the nilotinib 300-mg group was significantly reduced after false discovery rate (p* = .05). There was no statistically significant difference in CSF-triggered receptors on myeloid cells levels, plasma and CSF pan-tyrosine Abl, and plasma and CSF tyrosine 412 Abl (activity) via phosphorylation at 12 months (eTable 4 in Supplement 2).

### MDS-UPDRS and Timed Up and Go Tests

No differences were observed in MDS-UPDRS-I within and between all study groups (eTable 5, eTable 6, eTable 7 in Supplement 2) tested by a single rater (B.W.). Total MDS-UPDRS I-III and MDS-UPDRS I-IV scores were changed by 2.47 and 2.13 points between baseline and 12 months in the placebo group, but the nilotinib groups did not change (eFigure 2A, eTable 5, eTable 6 in Supplement 2). The placebo and nilotinib 300-mg groups remained stable at 12 months and after washout, but there was a significant improvement in MDS-UPDRS-III score between baseline and 15 months in the nilotinib 150-mg group (−2.82 points, 95% CI, −4.75 to −0.89 points). No significant differences were observed in MDS-UPDRS-IV. No statistically significant differences in MDS-UPDRS measurements were observed between groups (eTable 7 in Supplement 2). There were no statistically significant differences in the results of the Timed Up and Go test (assessment of risk of falling in PD) in the placebo and nilotinib 150-mg groups (eTable 5, eTable 6 in Supplement 2); however, the nilotinib 300-mg group performed significantly worse between baseline and 12 months (2.76 seconds; 95% CI, 0.73-4.79 seconds) and 15 months (2.53 seconds; 95% CI, 0.43-4.62 seconds). There were no significant differences in MDS-UPDRS measures between groups based on false discovery rate. There were no statistically significant differences in Parkinson Disease Questionnaire-39 (assesses the nonmotor symptoms in PD) between groups (eTable 7 in Supplement 2). No significant differences were observed in Montreal Cognitive Assessment score in the placebo and nilotinib 150-mg group (eTable 5, eTable 6 in Supplement 2), but the nilotinib 300-mg group significantly worsened (−1.04 points; 95% CI, −1.65 to −0.44 points) at 6 and 12 months compared with baseline.

## Discussion

Evaluation of the effects of nilotinib in patients with PD showed reasonable safety, although there were more SAEs in the nilotinib groups compared with the placebo group. Overall, there was a significant increase in the total number of SAEs in the nilotinib groups, but no significant difference was seen in cardiovascular SAEs, number of falls, and total number of AEs between all of the groups. It is likely that the increased number of SAEs, including the most common AEs, such as falls and hip fracture, urinary tract infection, skin diseases, pneumonia, and orthostatic hypotension, are PD-related issues that are unrelated to the study drug. In addition, some SAEs were due to preexisting cardiac and psychiatric (psychosis and hallucinations) conditions. Metastatic cancer, pulmonary embolism, and death occurred in the same participant and were probably not related to the study drug. Nilotinib is approved by the US Food and Drug Administration for treatment of chronic myeloid leukemia at a dose of 300 mg twice daily and carries a black-box warning of sudden death due to QTc interval prolongation and myocardial infarction,^16^ but no hypokalemia, hypomagnesemia, or long QT syndrome were observed in this study. Abelson inhibition seems to lead to cardiac and hematologic (myelosuppression) diseases, but our data indicate (eFigure 1, eTable 4 in Supplement 2) that plasma nilotinib does not inhibit Abl, suggesting that a low dose (≤300 mg once daily) may be safe in patients with PD. No myelosuppression was observed in this study. According to the nilotinib prescribing information, sudden deaths have been reported in 0.3% of patients with chronic myeloid leukemia treated with nilotinib in clinical studies of 5661 patients.^16^ Therefore, the small sample size in the present study cannot preclude a similar risk in the general PD population. Transient elevation of pancreatic enzymes and gastrointestinal symptoms in the nilotinib groups were rare and not clinically significant, did not require medical intervention, and resolved without intervention. Larger and long-term studies are needed to further confirm nilotinib safety in PD.

The pharmacokinetic data indicate that small amounts of nilotinib are detected in the CSF at 12 months in a dose-dependent manner. The CSF/plasma ratio of nilotinib is less than 1%, and this is consistent with the single-dose study performed in the same participants at baseline.^15^ However, the single dose of nilotinib resulted in dose-independent concentrations in the CSF, indicating that the participants were underexposed to nilotinib compared with concentrations noted at 1 year. The single-dose study also showed a significant increase of dopamine metabolism at lower doses (150-200 mg) compared with higher doses (300-400 mg) of nilotinib. At 12 months, the present study demonstrated an increase in CSF levels of dopamine metabolites in the nilotinib groups compared with the placebo group, which is consistent with previous results.^12,15^ DOPAC is a principal metabolite of dopamine and the primary metabolite in humans.^17^ Our data indicate an increase in CSF levels of HVA and CSF and plasma levels of DOPAC in the nilotinib 150-mg group. In contrast, CSF and plasma DOPAC levels were increased in the nilotinib 300-mg group, suggesting that dopamine metabolism is differentially increased with 150- and 300-mg doses of nilotinib. Furthermore, the brain may contribute 10% to 15% of the circulating plasma levels of HVA^18^; most of the circulating levels of HVA are due to intestinal metabolism of dopamine.

In the present study, the plasma HVA level did not change and CSF and plasma DOPAC levels were similar, suggesting that plasma DOPAC may be transported from the CSF. This dose-dependent change in dopamine metabolism seems to occur in the CSF. The CSF and plasma biomarkers are exploratory in this study, but adjustment for multiple comparisons shows that plasma DOPAC significantly increased in the 150-mg group, suggesting that dopamine metabolites may be used as biomarkers in further nilotinib studies in PD. The reduced level of levodopa at 12 months compared with baseline is due to dropouts from all groups. There was no pharmacokinetic analysis of levodopa in this study, and levodopa absorption is variable from individual to individual. However, the change in dopamine metabolites is unlikely due to absorption, because preclinical evidence demonstrates that nilotinib increases dopamine levels independent of levodopa in transgenic animals.^9,11,19,20,21^

The single-dose study showed that changes of CSF dopamine metabolism with lower (150-200 mg) doses of nilotinib may be concurrent with a reduction of oligomeric α-synuclein in a time-dependent manner.^15^ The CSF levels of α-synuclein oligomers increase in PD, while the total α-synuclein level decreases, compared with aged-matched controls.^22,23,24^ There was no change of CSF total α-synuclein at 12 months, but the level of oligomeric α-synuclein was significantly reduced in the 150-mg, but not the 300-mg, nilotinib group. A previous study showed more reduction of α-synuclein with a lower nilotinib dose (1 mg/kg) compared with a higher dose (10 mg/kg) in animal models of α-synucleinopathies.^10^ It is likely that a reduction of α-synuclein oligomers may result in improved dopaminergic neuron activity; therefore, the increased levels of CSF dopamine metabolites may be an index of functional activity of dopaminergic neurons in the brain. Furthermore, both doses of nilotinib significantly reduced the level of CSF p-tau, consistent with previous data that show that nilotinib lowers the level of tau in animal models of neurodegeneration.^7,8,9,10,11^

One study demonstrated that a reduction of tau in nilotinib-treated models of tauopathies results in enhanced astrocyte activity and improved neurotransmitter balance.^25^ Autophagy clearance of α-synuclein and tau is concurrent with improved astrocytic activity and balance of neurotransmitters, including dopamine.^9,10,25,26,27^ Collectively, the effects of nilotinib on CSF biomarkers indicate that a reduction of oligomeric α-synuclein and p-tau may improve dopamine metabolism in patients with PD. Nilotinib preferentially targets Abl^4,5,6^ and discoidin domain receptors.^4,5,6,7^ However, our data indicate no CSF Abl inhibition in the nilotinib groups compared with the placebo group (eFigure 1 in Supplement 2), suggesting that nilotinib effects on disease-related biomarkers are independent of Abl inhibition. These findings are consistent with profiling tyrosine kinase selectivity and efficacy, showing discoidin domain receptors as alternative nilotinib targets using lower concentrations of small molecule inhibitors of discoidin domain receptors.^4,5,6,7^ Nonetheless, all evidence of Abl inhibition by nilotinib comes from cell culture assays or animal models showing Abl inhibition in total brain lysates^9,11,19,20,21^; therefore, Abl inhibition in brain tissue cannot be ruled out.

No significant differences were seen in motor and nonmotor outcomes between the nilotinib groups and the placebo group. The study was underpowered for this analysis, so it is impossible to know if the lack of a difference relates to sample size, lack of efficiency, or another factor. No clinical worsening in MDS-UPDRS scores was observed in the nilotinib groups compared with the placebo group, and although there was a 1- to 1.5-point change in Montreal Cognitive Assessment score in the nilotinib 300-mg group, these changes are not clinically significant in patients with PD with an average Montreal Cognitive Assessment score greater than 26/30, indicating mild cognitive impairment. Our data revealed that patients in the placebo group who received an average levodopa dosage of 600 mg/d for 12 months showed a change of MDS-UPDRS I-III and MDS-UPDRS I-IV scores of 2.47 and 2.13 points, respectively (eTable 5 in Supplement 2), but there was no significant difference between the nilotinib groups, which did not change, and the placebo groups. The MDS-UPDRS-II scores worsened in the nilotinib 300-mg and placebo groups between baseline and 12 and 15 months, respectively, but not in the nilotinib 150-mg group. The MDS-UPDRS-III motor score was significantly different (−2.82 points) between baseline and 15 months in the nilotinib 150-mg group. The Earlier vs Later L-DOPA study investigated disease progression in patients with early PD who were treated for 10 months with levodopa, 150, 300, and 600 mg/d, and showed a change of MDS-UPDRS-III scores of 1.9, 1.9, and −1.4 points respectively.^28^ No change in Parkinson Disease Questionnaire-39 scores was seen in the nilotinib groups between baseline and 12 months. Taken together, the results of these exploratory clinical outcomes will guide the development of phase 3 studies.

### Limitations

This trial has limitations. The study was underpowered and performed in a single center and, as PD symptoms vary, different movement disorders centers may have a different approach to PD management and care, which may affect the results. In addition, 25% of screened participants were excluded based on electrocardiographic findings, suggesting that cardiac disease limited the enrollment of this subpopulation of patients with PD.

## Conclusions

This phase 2 study met its primary objectives and showed that nilotinib is reasonably safe, tolerated, and detected in the CSF of patients with PD. Exploratory biomarkers were altered in response to nilotinib treatment, and future phase 3 studies may identify dopamine metabolites, including HVA and DOAPC, as biomarkers of dopamine metabolism. Taken together, our results will guide the future development of a definitive phase 3 study to evaluate the effects of nilotinib as a disease-modifying drug in PD.

## Notes

Supplement 1. Trial Protocol

Supplement 2. **eMethods.** Detailed Methods **eReferences.** **eFigure 1.** Data Represent the Effects of 1-Year Nilotinib Treatment on Abl Activation via Tyrosine Phosphorylation and Plasma Dopamine Metabolites Compared to Placebo **eFigure 2.** Clinical Effects of Nilotinib in PD Using the Movement Disorders Society—Unified Parkinson Disease Rating Scale (MDS-UPDRS) and Parkinson’s Disease Questionnaire (PDQ)-39 **eTable 1.** Summary of EKG Values for All Participants Throughout All Study Visits Showing No QTc Prolongation in the Placebo Group **eTable 2.** Summary of EKG Values for All Participants Throughout All Study Visits Showing No QTc Prolongation in the 150mg Nilotinib Group **eTable 3.** Summary of EKG Values for All Participants Throughout All Study Visits Showing No QTc Prolongation in the 300mg Nilotinib Group **eTable 4.** Levels of Exploratory CSF and Plasma Biomarkers **eTable 5.** Pairwise Comparison of Clinical Endpoints Using Mean Differences Based on Raw Value at 6, 12 and 15 Months Compared to Baseline **eTable 6.** Average Values of Each Clinical Endpoint in Placebo, 150mg Nilotinib and 300mg Nilotinib Groups **eTable 7.** Statistical Analysis Showing in Mixed Effects Model and Test P Values and ANOVA Test for Group Differences **eTable 8.** Demographics Showing Participants Who Received Dopamine Agonists (DA) Alone Versus Participants Who Received DA+ Levodopa **eTable 9.** Concentration of Nilotinib in CSF and Plasma

Supplement 3. **Data Sharing Statement**
