# Supplementary material for: Nilotinib Effects on Safety, Tolerability, and Potential Biomarkers in Parkinson Disease: A Phase 2 Randomized Clinical Trial
Source: JAMA Neurol. 2019 Dec 16;77(3):309–17. doi: 10.1001/jamaneurol.2019.4200 (PMC6990742; doi:10.1001/jamaneurol.2019.4200)
Supplement: Supplement 1. — Trial Protocol [file jamaneurol-77-309-s001.pdf]

CLINICAL TRIAL PROTOCOL

A randomized, double blind, placebo-controlled study to evaluate the impact of low doses of Nilotinib treatment on safety, tolerability, pharmacokinetics and biomarkers in Parkinson's disease

Author: Charbel Moussa MD, PhD

Investigators:
Fernando Pagan, MD
Charbel Moussa, MD, PhD
Yasar Torres-Yaghi, MD (Fellow)
Ellen Valladez, MD (Fellow)
Abigail Lawler, MD (Fellow)
Michaeline Hebron, MS (Biomarkers and Data Manager)
Jaeil Ahn, PhD (Biostatistician)
Sorell Schwartz (Clinical Pharmacologist)
Barbara Wilmarth, NP (Nurse Practitioner)
Ashot Shekonyan, MD, PhD (Clinical Trial Manager)
Helen Howard, RN (Outreach Nurse Coordinator)

Georgetown University Medical Center (GUMC)
National Parkinson's Foundation Center for Excellence
Translational Neurotherapeutics Program (TNP)
4000 Reservoir Rd, NW,
Building D, Room 203-C
Washington D.C. 20057. USA.

Study Product: Nilotinib (Tasigna®, AMN107, Novartis)

Development phase: II

Draft or Version Number: v01 (Jan 23, 2017)

Property of Georgetown University Medical Center may not be used, divulged, published, or otherwise disclosed without the consent of Georgetown University

Clinical Trial Protocol Template Version 1.0, Nov, 2016

TABLE OF CONTENTS

List of Abbreviations

1- PROTOCOL SUMMARY AND RATIONALE	PAGE
1.1- Study Title	6
1.2- Version Number	6
1.3- Study Indication	6
1.4- Phase of Development	6
1.5- Background	6
1.6- Objectives and Endpoints	8
1.7- Study Rationale	12
2- STUDY DESIGN	13
2.1- Treatment	13
2.2- Drug Administration	13
2.3- Dosing Guidelines	13
2.4- Number of Planned Subjects and Treatment Plan	15
2.5- Study Population	15
3- VISIT SCHEDULE AND ASSESSMENTS	16
4- PATIENT POPULATION	20
4.1- Number of Patients & Centers	20
4.2- Inclusion Criteria	21
4.3- Exclusion Criteria	21
4.4- Randomization and Registration	22
4.5- Blinding	22
4.6- Un-blinding	22
4.7- Withdrawal of subjects	23
4.8- Handling of Withdrawals	23
4.9- Termination of Study	23
4.10- Protocol Adherence	23
5- SAFETY MONITORING REPORTING	24
5.1- All Non-serious Adverse Events	24
5.2- All Serious Adverse Events	24
5.3- All Reports of Drug Exposure during Pregnancy	25
5.4- All reports of misuse and abuse of Nilotinib	25
5.5- Assessment and Recording of Adverse Events	26
5.6- Assessment of Adverse Events	26
5.7- Relatedness of Adverse Event to Investigational Product	26
5.8- Recording of Adverse Events	27
6- STUDYMONTORING	28
6.1- Safety Monitoring	28

6.2- Data Safety and Monitoring Board (DSMB) 28
6.3- Study Monitor and auditing 28

7- Institutional Review Board (IRB) 29
7.1- Ethical Conduct of Study 29
7.2- Subject Information and Consent 29

8- STATISTICAL METHODS AND DATA ANALYSIS 30
8.1- Sample size determination 30
8.2- Analyses Plan 31

9- STUDY FEASIBILITY AND PRELIMINARY DATA 32
9.1- Preclinical Evidence 32
9.2- Human Studies 38
9.2. a- Pharmacokinetic studies 40
9.2. b- Pharmacodynamics 40
9.3.- Safety 41
9.3.1- Serious Adverse Events 41
9.3.2- Non-Serious AEs 41
9.3.3- QTc prolongation 42
9.3.4- Blood chemistry 44
9.4- Disease-related biomarker studies 45
9.5- Nilotinib ameliorates motor imprairment 47
9.6- Nilotinib improves cognitive symptoms 48

10-TREATMENTS ADMINISTERED 49
10.1- Name of the drug and all active ingredients 49
10.2- Pharmacological Class of the Drug 49
10.3- Structural Formula of the Drug 49
10.4- Formulation of the dosage form to be used 49
10.5- Summary of Previous Human Experience 50
10.6- Status of Drug in Other Countries 51
10.7- Drug Related Risks and Potential Side Effects 51

11-PRIOR AND CONCOMITANT THERAPY 52
11.1- Prohibited Medications and Contraindications 52

12-RATIONALE- NILOTINIB TO TREAT NEURODEGENERATIVE DISEASES 53

13-CLINICAL ASSESSMENTS AND OUTCOME MEASURES 55
13.1- Clinical Variables 55
13.2- Vital Signs, Height & Weight 55
13.3- Clinical Laboratory Assessments 55
13.4- Physical Examination 56
13.5- Neurological Examination 56
13.6- Adverse Events 57
13.7- MDS-UPDRS 57
13.8- PDQ-39 57

13.9- MOCA 57
13.10- C-SSRS 57
13.11- GDS 58
13.12- GSRS 58
13.13- CSF and Blood Biomarkers 58

14-BIOMARKERS 58
14.1- Rationale for CSF and plasma biomarkers 58
14.2- Pharmacokinetics 59
14.3- Phospho Abl (pan-tyrosine) ELISA 60
14.4- Cell death and exploratory biomarkers 60
14.5- Alpha-Synuclein ELISA 60
14.6- Homovanillic Acid ELISA 61
14.7- Total Tau and p-Tau181 measurement. 61
14.8- Human Neurodegenerative Disease Panels 61

15-DATA COLLECTION 62
15.1- Role of Data Management 62
15.2- Data Entry and Checks 62
15.3- Data Lock Process 62
15.4- Data handling and record keeping 62
15.5- Confidentiality 62
15.6- Retention of Records 62
15.7- Publications 63

16-LITERATURE REFERENCES 64

17-APPENDIX 69

PDQ-39 70
MoCA 73
C-SSRS Baseline 74
C-SSRS Since Last Visit 79
GSRS 83
GDS 84
MDS-UPDRS 85

LIST OF ABBREVIATIONS

AE	Adverse Event/Adverse Experience
AD	Alzheimer's Disease
ADL	Activity of Daily Living
Abl	Abelson Tyrosine Kinase
BBB	Blood Brain Barrier
CIB	Clinical Investigator's Brochure
CNS	Central Nervous System
CRF	Case Report Form
CSF	Cerebrospinal Fluid
C-SSRS	Columbia Suicide Severity Rating Scale
CRU	Clinical Research Unit
CML	Chronic Myeloid Leukemia
DSMB	Data Safety Monitoring Board
DLB	Dementia with Lewy bodies
DMSO	Dimethyl Sulfoxide
DA	Dopaminergic
EDC	Electronic Data Capture
ELISA	Enzyme-Linked Immunosorbent Assay
FTLD	Fronto-Temporal Lobar Dementia
GUMC	Georgetown University Medical Center
HVA	Homovanillic Acid
HCG	Human Chorionic Gonadotropin
ICH	International Conference on Harmonization
IND	Investigational New Drug Application
IHC	Immunohistochemistry
IRB	Institutional Review Board
ITT	Intent to treat
I.P.	Intraperitoneal
IV	Intravenous
LP	Lumbar Puncture
LB	Lewy Bodies
MoCA	Montreal Cognitive Assessment
MDS	Movement Disorders Society
N	Number (typically refers to subjects)
NMR	Nuclear Magnetic Resonance
NSE	Neuron Specific Enolase
NIH	National Institutes of Health
OHRP	Office for Human Research Protections
OHSR	Office of Human Subjects Research
PHI	Protected Health Information
PHRC	Partners Human Research Committee
PDGF	Platelet Derived Growth Factor
PI	Principal Investigator
PK	Pharmacokinetic

PDD	Parkinson's Disease with Dementia
PDQ-39	Parkinson Disease Questionaire-39
PD	Parkinson's disease
SAE	Serious Adverse Event/Serious Adverse Experience
SBP	Systolic Blood Pressure
SI	Site Investigator
SMC	Safety Monitoring Committee
SOP	Standard Operating Procedure
SN	Substantia Nigra
SCOPA-Cog	Scales for Outcomes in Parkinson's Disease-Cognition
Src	Protein Kinase SRC
TDP-43	Trans-Activation DNA Binding Protein-43
TKI	Tyrosine Kinase Inhibition
TNF	Tumor Necrosis Factor
VEGF	Vascular Endothelial Growth Factor
UPDRS	Unified Parkinson's Disease Rating Scale
UIS	University Information Service
WOCBP	Women of Child Bearing Potential

1- PROTOCOL SUMMARY AND RATIONALE

1.1- Study Title
A randomized, double blind, placebo-controlled study to evaluate the impact of low doses of Nilotinib (Tasigna®) on safety, tolerability, pharmacokinetics and biomarkers in Parkinson's disease

1.2- Version Number
Original Protocol

1.3- Study Indication
Parkinson's disease (PD)

1.4- Phase of Development
II

1.5- Background
Parkinson's disease (PD) is the second most common neurodegenerative disorder causing motor and non-motor symptoms. PD is characterized by death of dopaminergic (DA) neurons in the substantia nigra (SN) *pars compacta* and formation of inclusions known as Lewy bodies (LBs) that primarily contain aggregated alpha-Synuclein (1-3). Misfolded alpha-Synuclein accumulates intra-neuronally within LBs and SYN (alpha-Synuclein) is the highest genetic risk factor for PD followed by the microtubule associated protein Tau (MAPT) (4-8). L-Dopa replacement therapies are effective for symptomatic management in PD. However, a major challenge facing PD is to develop a therapy that can halt DA neurons death and alleviate symptoms. No therapeutic approach exists to alter the levels of alpha-Synuclein aggregates and halt DA death in PD. Therefore, degradation of misfolded alpha-Synuclein is the most desirable therapeutic approach. One mechanism to degrade alpha-Synuclein is autophagy (9-13), which is a process by which the cell can degrade its own contents. There is evidence that autophagy is impaired in neurodegeneration (14-21), leading to failure of degradation of accumulating protein aggregates, including misfolded alpha-Synuclein and Tau. Importantly, autophagy is exploited therapeutically in several diseases, including adult chronic myeloid leukemia (CML). Tyrosine kinase inhibitors (TKIs) induce autophagy (13,22,23), leading to destruction of rapidly dividing tumor cells in CML (22) and degradation of intracellular amyloids, including alpha-Synuclein and hyper-phosphorylated Tau (p-Tau) in PD and Alzheimer's disease (AD) models (13,22-25). Nilotinib (Tasigna®, AMN107, Novartis, Switzerland) is approved by U.S. Food and Drug Administration (FDA) and is well tolerated for CML treatment at oral doses of 600-800mg daily. Nilotinib penetrates the brain and promotes autophagic degradation of alpha-Synuclein and p-Tau, leading to survival of DA neurons and improvement of motor function in PD models (13,23-26). For these studies, Nilotinib (1-10mg/kg daily) was used at significantly less than the clinically approved dose (up to 1200mg daily) in CML (27-29). Additionally, Nilotinib levels peak in the mouse brain 4hrs after intraperitoneal (I.P) injection and wash out by 8hrs (13,23). This is important because the lower dose and short bio-availability (up to 8hrs) will prevent Nilotinib side effects and induce autophagic degradation of alpha-Synuclein and p-Tau in post-mitotic neurons, providing a strategy to prevent forcing neurons into apoptosis.

Based on strong pre-clinical evidence of the effects of Nilotinib on neurodegenerative pathologies, including autophagic clearance of neurotoxic proteins, neurotransmitters (dopamine and glutamate), immunity and behavior (25,26,30-34), we conducted an open label pilot clinical trial in advanced PD

with dementia (PDD) and Dementia with Lewy Body (DLB) (stage 3-4) patients. Participants (N=12) were randomized 1:1 to once daily oral dose of 150mg and 300mg Nilotinib for 6 months. Our data suggest that Nilotinib penetrates the brain and inhibits CSF Abelson (Abl) activity via reduction of phosphorylated Abl in agreement with pre-clinical data (13,35,36). Several studies (see biomarkers section) show that CSF alpha-Synuclein and Abeta42 are decreased and CSF total Tau and p-Tau are increased in PD and DLB (37-39). Our data show attenuation of loss of CSF alpha-Synuclein and Abeta40/42 with 300mg (50% of the CML dose) compared to 150mg Nilotinib after 6 months treatment. CSF homovanillic acid (HVA), which is an end by-product of dopamine, is significantly increased; and CSF total Tau and p-Tau are significantly reduced (N=5, $P<0.05$) with 300mg Nilotinib between baseline and 6 months treatment. Despite the reduction of L-Dopa replacement therapies in our study, UDPRS I-IV scores improved with 150mg (3.5 points) and 300mg (11 points) from baseline to 6 months and worsened (13.7 points and 11.4 points) after 3 months withdrawal of 150mg and 300mg, respectively. Other non-motor functions e.g. constipation was resolved in all patients and cognition was also improved (3.5 points) using both the Mini-Mental Status Exam (MMSE) or the Scales for Outcomes in Parkinson's Disease-Cognition (SCOPA-Cog) between baseline and 6 months. MMSE scores returned to baseline after 3 months of Nilotinib withdrawal. These data are very compelling to evaluate the effects of Nilotinib in a phase II, randomized, double-blind, placebo-controlled trial in patients with PD.

We hypothesize that:

Primary outcomes: Nilotinib will be safe and tolerated in individuals with PD.
We also hypothesize that low daily oral doses of Nilotinib will lead to CSF penetration.

Secondary outcomes: Nilotinib will increase CSF HVA ≥50% compared to placebo.

Clinical outcomes: Low daily doses of Nilotinib will improve motor, autonomic and cognitive symptoms in PD.

We predict that Nilotinib will lead to CSF inhibition of Abl, stabilize CSF alpha-Synuclein and reduce CSF markers of cell death, including total Tau and p-Tau levels, neuron specific enolase (NSE) and S100B.

1.6- Objectives and Endpoints

Primary outcomes, we will evaluate the effects of Nilotinib on safety and tolerability

1- Safety will be measured using the occurrence of adverse events (AEs) and serious adverse events (SAEs) deemed to be possibly, probably, or definitely related to the study drug. AEs of interest are defined as QTc prolongation, myelosuppression, hepatotoxicity and pancreatitis as listed in Table 1. These AEs will be tracked over the course of the trial and reviewed by the data and safety monitoring board (DSMB) at scheduled meetings and in real time. SAEs and AEs are known to be related to drug use at 800mg daily in cancer. A small safety trial using lower oral daily doses of 150 mg and 300 mg Nilotinib in 12 PD patients showed one cardiac SAE over a six-month treatment period. Based on preliminary clinical data, investigator's brochure (IB) and scheduled EKGs and lab tests, SAEs and AEs will be evaluated real-time and on case-by-case basis.

2- Tolerability for a given participant will be defined as the ability of participants to remain on treatment. Overall tolerability of the drug will be defined as an acceptable number of up to 25% discontinuations.

Warnings and precautions: Nilotinib (Tasigna) prescribing information includes risks associated with arterial vascular occlusive events, including ischemic heart disease-related cardiac events, peripheral arterial occlusive disease and ischemic cerebrovascular events.

Nilotinib has been shown to prolong cardiac ventricular repolarization as measured by the QT interval on the surface EKG in a concentration-dependent manner. Prolongation of the QT interval can result in a type of ventricular tachycardia called torsade de pointes, which may result in syncope, seizure, and/or sudden death. **The baseline QTc range for inclusion into this study is 350-460 ms. If QTc prolongs ≥ 60 ms from baseline of individual participants AND to a value ≥ 480 ms, Nilotinib must be held and participants will be re-evaluated in one week on a case-by-case basis in consultation with a cardiologist before continuing on Nilotinib. If QTcF prolongs ≥ 500 ms then Nilotinib will be completely stopped and participants will be withdrawn from the study.**

Nilotinib should not be used in patients who have hypokalemia, hypomagnesemia or long QT syndrome. Before initiating Nilotinib electrolyte, calcium and magnesium blood levels should be tested. Hypokalemia or hypomagnesemia must be corrected prior to initiating Nilotinib and these electrolytes should be monitored periodically during drug treatment [See Table 3].

Gastrointestinal (GI) hemorrhage and effusions (including pleural effusion, pericardial effusion, ascites) or pulmonary edema were also reported in a small percentage of patients taken Nilotinib and GI hemorrhage and effusions should be monitored at every visit.

The bioavailability of Nilotinib is increased with food, thus Nilotinib must not be taken with food. No food should be consumed for at least 2 hours before and for at least 1 hour after the dose is taken.

Significant prolongation of the QT interval may occur when Nilotinib is inappropriately taken with food and/or strong CYP3A4 inhibitors and/or medicinal products with a known potential to prolong QT. Therefore, co-administration with food must be avoided and concomitant use with strong CYP3A4 inhibitors and/or medicinal products with a known potential to prolong QT should be avoided *[see exclusion criteria, section 4.3].*

The presence of hypokalemia and hypomagnesemia may further prolong the QT interval.

Table 1 summarizes some key laboratory tests to monitor AEs and SAEs and includes stopping rules and withdrawal for individual patients.

Table 1- Monitoring laboratory tests for drug safety and stopping rules or withdrawal. Grade 3 classification of organ toxicity is according to standardized definitions of AEs published by the National Cancer Institute (NCI) of the National Institutes of Health (NIH), known as the Common Terminology Criteria for AEs (CTCAE, Version 4.0) or common toxicity criteria.

Grade 1 Mild; asymptomatic or mild symptoms; clinical or diagnostic observations only; intervention not indicated.

Grade 2 Moderate; minimal, local or noninvasive intervention indicated; limiting age-appropriate instrumental activities of daily living (ADL).

Grade 3 Severe or medically significant but not immediately life-threatening; hospitalization or prolongation of hospitalization indicated; disabling; limiting self-care ADL.

Grade 4 Life-threatening consequences; urgent intervention indicated.

Grade 5 Death related to AE.

Assessments	Toxicity Criteria	Endpoints
**Myelosuppression**	Thrombocytopenia and Neutropenia	Perform complete blood counts (CBC) every 2 weeks for the first 2 months and then monthly thereafter, or as needed.<br>Withhold Nilotinib for hematological toxicities if absolute neutrophil count (ANC) <1.0 x 10^9 /L and/or platelet count <50 x 10^9 /L.<br>Monitor blood counts every 7 days and if problem resolves resume treatment within 2 weeks at prior dose.<br>If myelosuppression recurs after resumption of 300 mg Nilotinib, the dose should be lowered to 150 mg and patient's blood counts must be monitored every 7 days.<br>If Myelosuppression recurs with 150 mg, patients must be withdrawn from the study.
**QT Prolongation**	EKGs with a QTc ≥480 ms<br>and/or an increase of QTc ≥ 60 ms from baseline	Withhold Nilotinib, and perforrm an analysis of serum potassium and magnesium, and if below lower limit of normal, correct with supplements to within normal limits. Concomitant medication usage must be reviewed. Patients with prolonged QTc ≥60 ms change from baseline or QTc ≥480 ms should be withdrawn from the study unless there is a causative electrolyte abnormality that has been corrected or CYP inhibitor that may be withdrawn.<br>Resume within 2 weeks at prior dose if QTcF returns to <450 ms and to within 20 ms of baseline.<br>If QTcF is between 450 ms and 480 ms after 2 weeks, reduce the 300 mg dose to 150 mg dose.<br>Repeat EKG 7 days after dose adjustment and if QTcF returns to ≥480 ms following dose reduction or withholding, Nilotinib must be discontinued.

**Hepatotoxicity**	Elevated hepatic transaminases ≥ Grade 3	Withhold Nilotinib and monitor hepatic transaminases, including alanine aminotransferase (ALT), aspartate aminotransferase (AST) and alkaline phosphatase every 7 days. If transaminase level returns to baseline, resume treatment at the prior dose.<br>If hepatotoxicity recurs after resumption of 300 mg Nilotinib, the dose should be lowered to 150 mg and patient's must be monitored every 7 days. If hepatotoxicity recurs with 150 mg, patients must be withdrawn from the study.
**Bilirubin**	Elevated Bilirubin ≥ Grade 3	Withhold Nilotinib and monitor bilirubin every 7 days. If bilirubin level returns to baseline, resume treatment at the prior dose.<br>If bilirubin elevation recurs after resumption of 300 mg Nilotinib, the dose should be lowered to 150 mg and patient's must be monitored every 7 days. If bilirubin elevation recurs with 150 mg, patients must be withdrawn from the study.
**Pancreatitis**	Elevated serum lipase and amylase ≥ Grade 3	Withhold Nilotinib and monitor lipase and amylase every 7 days. If lipase and amylase levels return to baseline, resume treatment at the prior dose.<br>If pancreatic toxicity recurs after resumption of 300 mg Nilotinib, the dose should be lowered to 150 mg and patient's must be monitored every 7 days. If pancreatic toxicity recurs with 150 mg, patients must be withdrawn from the study.

3- Pharmacokinetics: We will measure the CSF concentration of Nilotinib.

Secondary outcomes: We will determine the effects of Nilotinib on primary biomarkers, including

- **1)** changes of CSF levels of HVA between baseline and 12 months. Based on the data collected from the proof of concept study in PD we predict an increase in CSF HVA levels following Nilotinib treatment.

We hypothesize ≥50% HVA increase between baseline and 12 months treatment.

We will also measure the CSF concentration of surrogate/exploratory biomarkers: including:

- measurement of phosphorylated Abl (activity) in CSF as evidence of CNS Abl inhibition, indicating direct CNS target engagement
- CSF α-Synuclein (total and oligomeric), total Tau and p-Tau, neuron specific enolase (NSE) and neuron and glial marker S100B.
- We will use ELISA to measure CSF and plasma neuro-inflammatory/modulatory markers, including interleukins (IL)- 1α&β, 2, 3, 4, 5, 6, 7, 8 (CXCL8), 9, 10, 12, 13, 15, 17 α, and chemokines (C-C) including, CXCL10, CCL2, CL7, CCL22, CCL3, CCL4, platelet-derived growth factor (PDGF)-AA, PDGF-AB/BB, CCL5, CX3CL1 (fractalkine), Tumor necrosis

growth factor (TNF)-α, transforming growth factor (TGF)-α, and vascular endothelial growth factor (VEGF).

Clinical outcomes will include assessment of:

a- Motor function using MDS-UPDRS, PDQ-39 and timed-up and go
b- Cognitive function using the Montreal Cognitive Assessment (MoCA).
c- Autonomic nervous system, including orthostatic and gastrointestinal motility

Based on preclinical and clinical studies (see below), **we hypothesize that Nilotinib will be safe and tolerable in individuals with PD.** We also predict that Nilotinib will be detected in the CSF and and will inhibit Abl activity. Nilotinib will also increase CSF HVA levels and improve clinical outcomes. Nilotinib may also stabilize CSF levels of alpha-Synuclein, affect cell death markers, including total Tau, p-Tau, NSE and S100B. We further hypothesize that we may see evidence of change in surrogate and biomarkers of pathophysiology as outlined in our secondary outcomes that will help us to build a better clinical development program going forward.

1.7- Rationale to advance to Phase II Nilotinib trial in PD (Study Rationale)

We performed an open label phase I clinical trial using two commercially available doses of Nilotinib (150 and 300mg capsules) in patients with advanced PDD and DLB. These indications have some overlapping pathologies and clinical symptoms and share common plasma and CSF biomarkers, including alpha-Synuclein, Abeta42/40, total Tau and p-Tau (181, 231). We obtained preliminary data showing that Nilotinib crosses the BBB and is detected in the CSF, suggesting Abl inhibition and downstream target engagement (alpha-Synuclein, Tau and Abeta) in the CNS (pharmacodynamics). Nilotinib increased CSF HVA levels as a downstream biomarker of dopamine metabolism. These data provide strong feasibility to test Nilotinib in a larger placebo-controlled, phase II clinical trial to demonstrate safety, tolerability, pharmacokinetics/pharmacodynamics (chart 1) and changes in disease biomarkers and clinical outcomes in patients with PD.

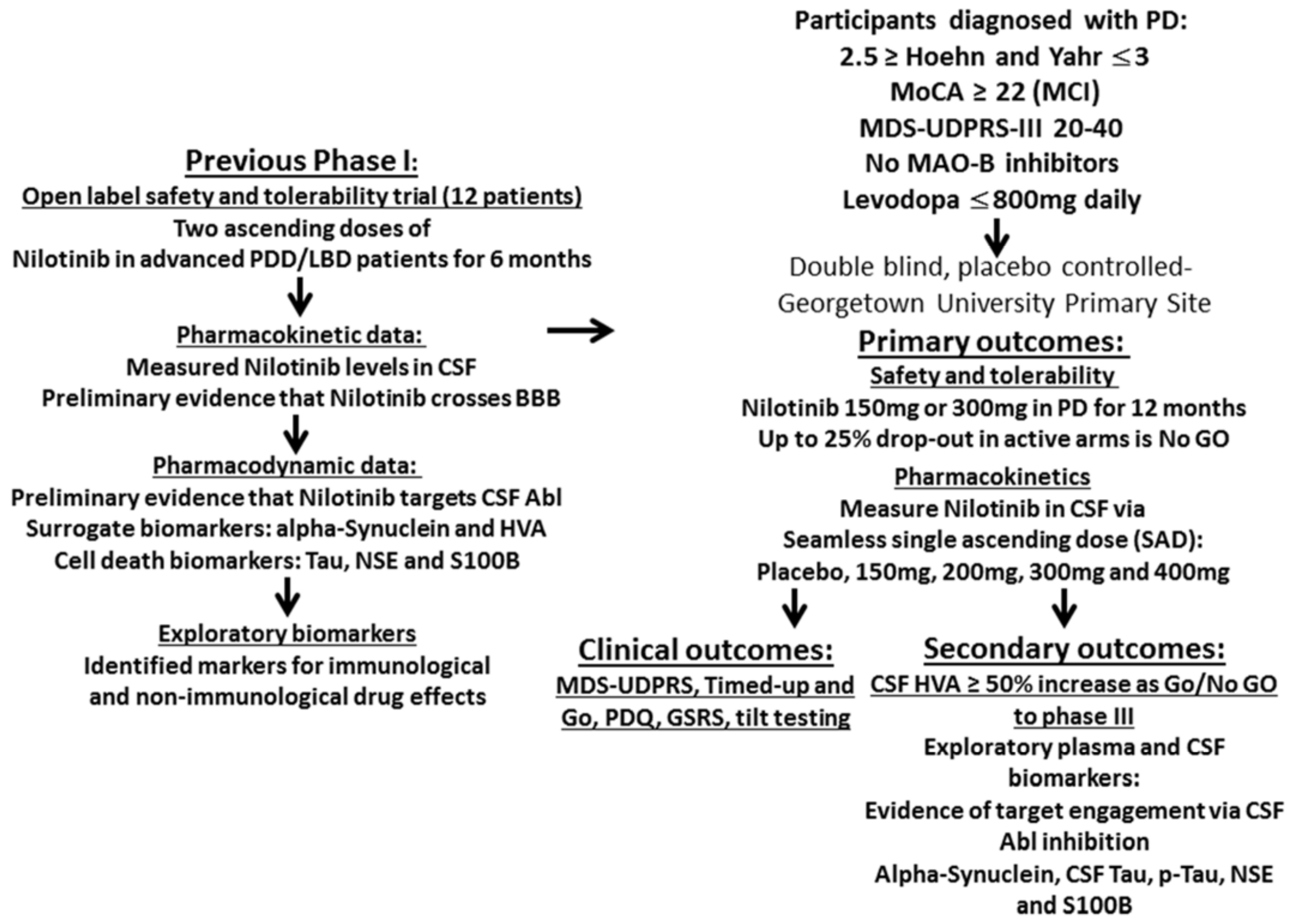

Chart 1- Summary of research activities in phase I Nilotinib clinical trial that provide strong rationale and preliminary evidence supporting development of clinical research into a double-blind, placebo controlled, phase II trial in early PD.

2- Study Design

3- We propose to perform a randomized, double-blinded, placebo-controlled, phase II clinical trial and aim to evaluate the impact of low doses of Nilotinib treatment on safety, tolerability, pharmacokinetics and biomarkers in patients with PD (Chart 2).

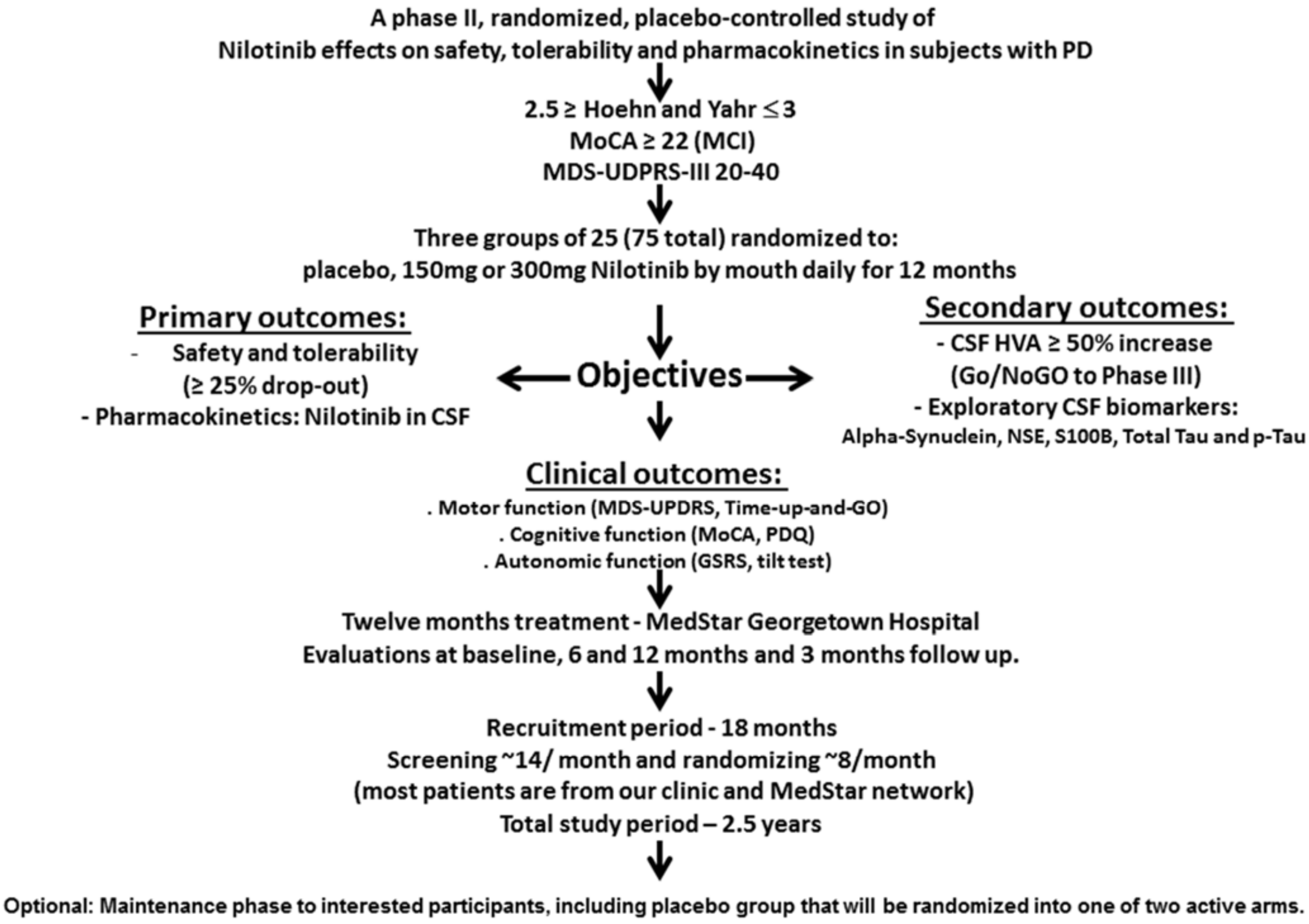

Chart 2- Study design of a randomized, double blind placebo controlled, phase II trial of Nilotinib in PD.

2.1- Treatment: Seventy five (75) participants will be recruited and randomly assigned 1:1:1 to placebo (group 1), 150mg (group 2) or 300mg (group 3).

2.2- Drug Administration: Nilotinib will be taken orally once daily (taken without a meal) for 12 months and 3 months follow up. Study partners must provide written informed consent prior to screening. Based on data obtained in our proof of concept phase I study, a phase II study will primarily examine the effects of Nilotinib on safety and tolerability and clinical outcomes. An optional open-label maintenance protocol will be performed after follow-up for one year.

2.3- Dosing Guidelines

One or two 150 mg capsules of Tasigna or matching placebo will be administered orally once a day. Key elements will be included in the educational brochure a special warnings and pre-cautions, including (Table 2), including:

- Brief background on Tasigna, its authorized indication and posology

- Information on the cardiac risks associated with the use of Tasigna
- That Tasigna can cause prolongation of the QT interval and that patients at risk of arrhythmia, especially torsade de pointes, should not be prescribed Tasigna.

The need to avoid co-prescription with any other medicines that might prolong the QT interval

- Caution in prescribing to patients with a history of or risk factors for coronary heart disease
- That Tasigna may cause fluid retention, cardiac failure and pulmonary edema
- That Tasigna is metabolized by CYP3A4 and that strong inhibitors or inducers of this enzyme may significantly affect exposure to Tasigna.
- That inhibitors may increase the potential for adverse drug reactions in particular QT interval prolongation
- To warn participants about over the counter (OTC) medicines in particular St John's Wort
- The need to inform patients about the effects of food on Nilotinib (Tasigna)
- Not to eat within two hours before and one hour after taking Nilotinib (Tasigna)
- The need to avoid foods such as grapefruit juice which inhibit CYP3A4 enzymes

Planned exposure (e.g. Duration of the study administration)

We anticipate this project to be completed in 2 years, including 12 months for pre-screening and enrolment, 6-12 months for participant treatment, neurological and safety examinations and 3-6 months for post treatment evaluation as well as sample and statistical data analysis.

Table 2- Summary of primary, secondary and exploratory objectives with the associated endpoints and evaluation criteria:

**Outcomes**	**Objective**	**Endpoint**
**Primary**	Safety, tolerability and pharmacokinetics	1- Safety will be measured using the occurrence of AEs) and SAEs deemed to be possibly, probably, or definitely related to the study drug. AEs of interest are defined as QTc prolongation, myelosuppression, hepatotoxicity and pancreatitis as listed in Table 1 and Novartis IB, on which we base our power consideration. These AEs will be tracked over the course of the trial and reviewed by the data and safety monitoring board (DSMB) at scheduled meetings and in real time. SAEs and AEs are known to be related to drug use at 800mg daily in cancer. A small safety trial using lower oral daily doses of 150mg and 300mg Nilotinib in 12 PD patients showed one cardiac SAE over a six-month treatment period. Based on preliminary clinical data, IB and scheduled EKGs and lab tests, SAEs and AEs will be evaluated real-time on case-by-case basis.<br>2- Tolerability for a given participant will be defined as the ability of participants to remain on treatment. Overall tolerability of the drug will be defined as an acceptable number of up to 25% discontinuations.<br>3- We will measure the CSF concentration of Nilotinib.
**Secondary**	Biomarkers	CSF levels of HVA between baseline and 12 months.

		**We hypothesize ≥50% HVA increase between baseline and 12 months treatment as GO/NoGO to phase III.** <u>surrogate/exploratory biomarkers:</u> - measurement of CSF phosphorylated Abl - CSF α-Synuclein, total Tau and p-Tau, NSE) and S100B. - We will use ELISA to measure CSF and plasma neuro-inflammatory/modulatory markers.
**clinical outcomes**	Motor	MDS-UPDRS, PDQ-39 and timed-up and go
	Cognition	MoCA
	Autonomic	Orthostatic and bowel movement

2.4- Study Objectives and Endpoints

2.5- Number of Planned Subjects and Treatment Plan
Up to 100 subjects will be screened for the study with the goal of enrolling 75 for treatment (25 placebo, 25 low dose (150 mg Nilotinib) and 25 high dose (300 mg Nilotinib)).

2.6- Population

This study will be conducted in subjects with PD with Hoehn & Yahr between 2.5 and 3. Montreal Cognitive Assessment (MoCA) score ≥22 (inclusive) will be used to identify mild cognitive impairment (MCI) at screening. Eligible participants must not be on MAO-B inhibitors (Rasageline or Selegeline) for 6 weeks and must not be on ≥800mg Levodopa daily.

4-VISIT SCHEDULE AND ASSESSMENTS

Screening. This visit (Table 3) will determine study eligibility. Potential participants and their study partners must review and sign an informed consent form (ICF) prior to any study-related procedures. Information regarding demographics, concurrent medications, and medical history will be gathered from the participant and study partner at the screening visit. Prior to any study-related activities, subjects will be thoroughly informed on all aspects of the study and will be requested to sign an ICF. Prior to obtaining written informed consent, information will be given at a complexity level that is understandable by the subject in both oral and written form by staff. Participants will also be informed about the drug safety and side effects.

Visits/month	Screening	Baseline	0.5	1	1.5	2/3/4/5	6	7/8/9	10/11	12	15
Consent	X										
Demographis	X										
Vitals	X	X	X	X	X	X	X	X	X	X	X
H&P	X	X	X	X	X	X	X	X	X	X	X
Neuro Exam	X	X	X	X	X	X	X	X	X	X	X
Orthostatic	X	X	X	X	X	X	X	X	X	X	X
EKG	X	X	X	X	X	X	X	X	X	X	X
Blood draw*	X	X	X	X	X	X	X	X	X	X	X
CSSRS	X	X	X	X	X	X	X	X	X	X	X
GSRS	X	X	X	X	X	X	X	X	X	X	X
GDS	X	X	X	X	X	X	X	X	X	X	X
Hoehn & Yahr	X	X					X			X	X
MoCA	X	X					X			X	X
PDQ-39	X	X					X			X	X
Timed-up&GO	X	X					X			X	X
MDS- UPDRS	X	X					X			X	X
LP		X								X	

Table 3- A detailed schedule of 17 visits and the assessments done at each visit. History and Physical (H&P), Electrocardiograms (EKG), Columbia Suicide Severity Rating Scale (CSSRS), Gastrointestinal Symptoms Rating Scale (GSRS), Geriatric Depression Scale (GDS), Montreal Cognitive Assessment (MoCA), Parkinson's Disease Quality of Life (PDQ)-39,Movement Disorders Society-Unified Parkinson's Disease Rating Score (MDS-UPDRS), Lumbar Puncture (LP).

Monitoring and laboratory tests will be performed at the screening visit and at every visit 2 weeks after Nilotinib initiation for the first 2 months and every month thereafter for one year (Table 3). Vital signs will be recorded with comprehensive physical and neurological examinations. EKGs will be performed with standard blood chemistry, including Cholesterol, low density lipoprotein (LDL), high density lipoprotein (HDL), albumin, total protein, alkaline phosphatase, total bilirubin, creatinine, calcium, chloride, sodium, potassium, magnesium, inorganic phosphorus, bicarbonate, creatine phosphokinase (CPK), gamma-glutamyl transferase (γ-GT), lactate dehydrogenase (LDH), lipase, α-amylase, alanine aminotransferase (ALT), aspartate aminotransferase (AST), blood urea nitrogen (BUN), thyroid stimulating hormone (TSH), vitamin B12, and glucose. A standard hematology panel including, complete blood count (CBC) with differential counts will be performed. Hemoglobin (Hb), hematocrit, red blood cell count, platelet count, and white blood cell (WBC) with differential count will be measured. In addition, prothrombin time-international normalized ratio (PT-INR) will be measured for assessment of coagulation. Should treatment with any HMG-CoA(or 3-hydroxy-3-methylglutaryl-coenzyme A) reductase inhibitor (a lipid lowering agent) be needed to treat lipid elevations, evaluate

the potential for a drug-drug interaction before initiating therapy as certain HMG-CoA reductase inhibitors are metabolized by the CYP3A4 pathway. If test results warrant therapy, local standards of practice and treatment guidelines will be followed.

The Columbia Suicide Severity Rating Scale (C-SSRS) and Geriatric Depression Scale (GDS), orthostatic exams and the Gastrointestinal Rating scales (GSRS) will be performed at the screening visit and at every visit as indicated in Table 3.

Baseline visit will be scheduled 2-4 weeks after screening and results from all screening procedures will be reviewed and all inclusion/exclusion criteria must be met prior to baseline assessments. The baseline procedures may be completed over several visits. After recruitment, an open label randomized single dose (RSD) study will be performed and participants will be randomized into 5 groups (1:1:1:1:1) receiving a single daily dose (once) of placebo, 150 mg, 200 mg, 300 mg or 400 mg Nilotinib. Blood collection and lumbar puncture (LP) will be staggered as shown in chart 3. Following 1 week wash-out period all participants will be randomized to a double-blind placebo-controlled study for 12 months as shown in chart 4. Participants will be randomized into 3 groups of placebo, 150 mg and 300 mg doses for 12 months and LP and blood will be collected to obtain physiological population pharmacokinetics. This experiment will be conducted to obtain stronger PK data by adding 200 mg and 400 mg doses. We intend to perform this seamless open label RSD experiment as part of a larger long term study with two active arms (150 mg and 300 mg) and placebo (Chart 3). Our preliminary data indicate that 150 mg and 300 mg are safe and suggest clinical and biomarker signals, therefore we will use these doses for active arm treatment to determine safety and tolerability. We predict that a higher rate of drop-outs will take place in the 300 mg group compared to 150 mg as our proof of concept study indicated that participants in the 300 mg group were more tired (fatigue), had one SAE (myocardial infract), two episodes of QTc prolongation and one participant was taken out of the 300 mg into the 150 mg group

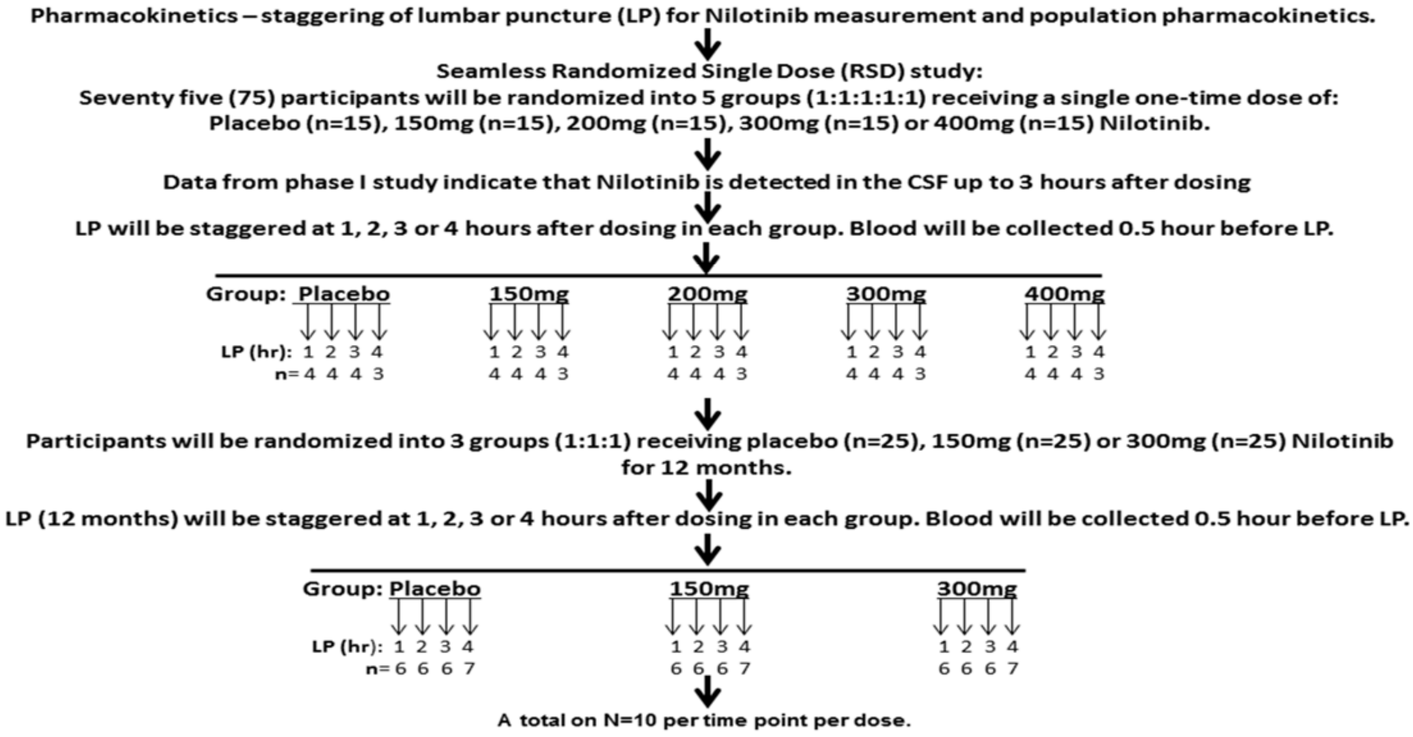

Chart 3- Study design of a randomized, double blind placebo controlled, phase II trial of Nilotinib in PD.

due to QTc prolongation. Therefore, we will only also use the highest concentration of 400 mg Nilotinib only once to determine PK in the RSD study.

CSF collection via LP and blood draw will be performed for biomarker pharmacokinetics, pharmacodynamics and biomarker measurement (CSF alpha-Synuclein, CSF total Tau/p-Tau18, HVA

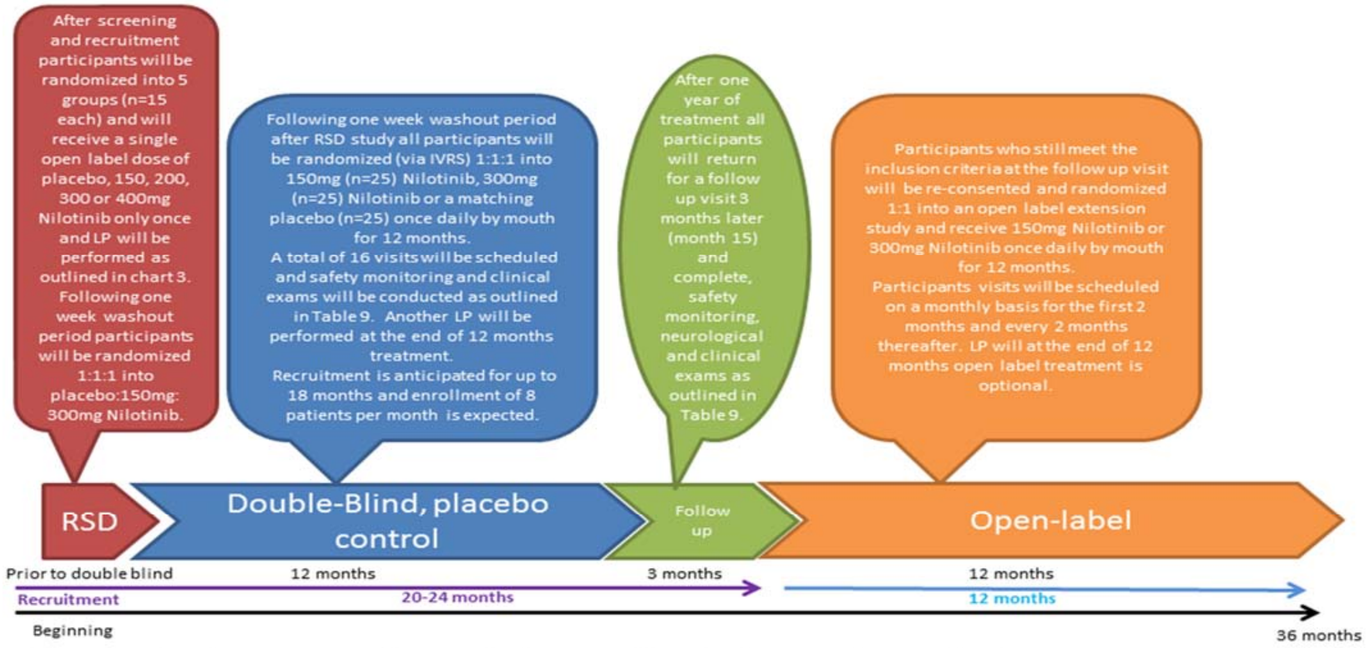

Chart 4- Graphical presentation of the study design and length of each study period and treatment arms in the double-blind and open label phases of study. The Randomized Single Dose (RSD) is seamless and consists of only one dose of drug or placebo prior to randomization into the double-blind phase after one week wash-out period. Randomization into the Open-label phase will be after the 3 months follow up visit.

and inflammatory/neurotrophic markers). Blood will also be collected for monitoring laboratory tests as indicated above. Vital signs, brief physical and neurological examination along with documentation of concurrent medications and AEs will be performed every visit. LPs from 5-10 patients will be performed pre-dose at 12 months in the double blind placebo-controlled phase (Chart 4) and an additional blood draw for PK studies will also be performed at 6 months during the double blind phase. To study the long term safety effects of Nilotinib in the PD population, participants will be re-consented and randomized for an open label study for 12 more months into either 150mg or 300mg Nilotinib (chart 4). Table 3 provides a detailed summary of activities that will be performed to measure the primary outcomes (safety and tolerability) and secondary clinical and biomarkers outcomes (pharmacokinetics/CSF HVA levels) for the double-blind placebo-controlled trial. The same safety monitoring tests and clinical assessments will be performed every month during the open label trial. LP at 12-month end of open label trial is optional. Blood draws for PK and biomarkers studies will also be performed at 6 and 12 months of open label trial.

A pregnancy test will be performed at each visit for all women of child bearing potential.

At baseline, 6, 12 and 15 months - Motor assessments will be performed via standardized tests such Movement Disorder Society-Unified Parkinson Disease Rating Scale (MDS-UPDRS), Parkinson's Disease Questionnaire (PDQ)-39 and Timed-Up and Go. **Cognitive assessments** will be performed via Montreal Cognitive Assessment (MoCA). LP will be performed at baseline and 12 months visits. A follow up visit is scheduled at 15 months. This 15-month wash-out visit will be a revolving visit when participants will be re-consented to participate in an open label extension study for 1 year.

Table 3.1- describes what will happen at all the visits in open label extension (OLE) study:

Visits/month (OLE)	baseline	1m	2m	4m	6m	8m	10m	12 m	15m
Consent	X								
Vitals	X	X	X	X	X	X	X	X	X
H&P	X	X	X	X	X	X	X	X	X
Neuro Exam	X	X	X	X	X	X	X	X	X
Orthostatic	X	X	X	X	X	X	X	X	X
EKG	X	X	X	X	X	X	X	X	X
Blood draw*	X	X	X	X	X	X	X	X	X
CSSRS	X	X	X	X	X	X	X	X	X
GSRS	X	X	X	X	X	X	X	X	X
GDS	X	X	X	X	X	X	X	X	X
Hoehn & Yahr	X				X			X	X
MoCA	X				X			X	X
PDQ-39	X				X			X	X
Timed-up&GO	X				X			X	X
MDS- UPDRS	X				X			X	X
LP (optional)								X	

Open Label Extention (OLE) schedule of events- A detailed schedule of 9 visits and the assessments done at each visit. History and Physical (H&P), Electrocardiograms (EKG), Columbia Suicide Severity Rating Scale (CSSRS), Gastrointestinal Symptoms Rating Scale (GSRS), Geriatric Depression Scale (GDS), Montreal Cognitive Assessment (MoCA), Parkinson's Disease Quality of Life (PDQ)-39,Movement Disorders Society-Unified Parkinson's Disease Rating Score (MDS-UPDRS), Lumbar Puncture (LP).

***Blood draw** will be performed for standard blood chemistry as in table 3.

The LP should be performed 1-4 hours after your last dose of Nilotinib at 12 months OLE visit. An additional 15 ml of blood will be drawn for measurement of disease biomarkers (alpha-synuclein and dopamine) in your blood.

Medication Compliance Check. Site staff will remind subjects to bring unused study drug with them **at each visit** as well as the completed drug compliance diary. Site staff will count the number of capsules remaining at each in-person clinic visit for which the subject brings the unused drug with him/her. This information will be recorded in the subject specific drug accountability form. The total number of returned medication will be reviewed against the drug compliancy diary. If drug compliance is determined to be problematic site staff will review with the subject the importance of using the study drug as per protocol. Any dose adjustments will be documented on the drug exposure form (indicating start and stop date). Additionally site staff will check the subject's overall compliance with the study requirements. This will also include checks of protocol compliance, and concomitant medication use, in addition to the proper use of study drug in order to assess the reliability of subject generated data.

Drug Related Risks and Potential Side Effects

Nilotinib has a number of side effects as stated in the IB, including gastrointestinal complications, vomiting and nausea, and in some rare cases dizziness (27). Abl has an essential role in mammalian heart growth and development and adult complications with Abl inhibition, include edema, nausea/vomiting, muscle cramps, neutropenia, thrombocytopenia, fever, liver toxicity, arthralgia, and exanthema/rash (40). Side effects are identified for Abl in homozygous Abl mutant mice, which display dramatically enlarged hearts due to abnormally increased cardiomyocyte proliferation during later stages of embryogenesis (41). Disruption of *Abl* in mice results in neonatal lethality accompanied by

pleiotropic developmental defects with variable penetrance, including ranting, splenic and thymus atrophy, B cell lymphopenia, dysfunctional osteoblasts, and foreshortened crania (42-44). However, clinical use of Nilotinib (600-800mg daily) is fairly tolerated in CML patients, but no clinical and longitudinal data exist about the long term side effects of this drug since its approval in 2007. Therefore, clinical use of Abl inhibition may have dose-limiting toxicity but we will use much lower doses in the current studies (see study design). Importantly, Nilotinib is washed out of the brain within several hours (13,23), so lower dose and prolonged period of administration may decrease misfolded protein levels, leading to slowing of motor, non-motor and cognitive decline. Furthermore, to evaluate adverse drug effects, all patients will be regularly (every two week for the first two months) monitored for specific warnings, including <u>prolongation of QT interval</u>. Prior to administration and periodically, patients will be monitored for hypokalemia or hypomagnesaemia. EKGs will also be obtained to monitor the QTc at baseline, 12 days after initiation, and periodically thereafter as detailed in Table 3. Nilotinib will not be administered to patients with hypokalemia, hypomagnesaemia, or long QT syndrome. Concomitant drugs known to prolong the QT interval and strong CYP3A4 inhibitors will also be avoided.
Nilotinib should be taken on an empty stomach 2hrs after eating to ensure appropriate absorption and no food should be consumed one hour after Nilotinib. The following SAEs can occur with Nilotinib:

1- Myelosuppression
2- QT Prolongation
1- Sudden Deaths
2- Cardiac and Arterial Vascular Occlusive Events
3- Pancreatitis and Elevated Serum Lipase
4- Hepatotoxicity
5- Electrolyte Abnormalities
6- Hemorrhage
7- Fluid Retention

<u>4-*PATIENT POPULATION</u>

4.1- Number of Patients & Centers

This is a single center study that will be conducted at Georgetown University Medical Center (GUMC) Clinical Research Unit (CRU) of Georgetown-Howard Universities Center for Clinical and Translational Science (GHUCCTS). GHUCCTS encompasses MedStar Health Research Institute (10 hospitals), including Georgetown University Hospital (GUH), Howard University, the Washington DC VA Medical Center (with its hospital and five clinics) and the Oak Ridge National Laboratory. CRU GHUCCTS is NIH-funded and combines the five institutions into one research powerhouse, creating one of the largest clinical trials networks in the country.
A total of 75 participants with PD with MCI (MoCA≥22) will be enrolled. We anticipate screening 100 individuals to enroll a total of 75 participants over 18-month period. The Movement Disorders Program (MDP) at MedStar Georgetown Hospital (GUH), a National Parkinson's Foundation Center for Excellence, will screen on overage 14 patients and enroll 8 per month. There are no competing clinical trials for PD at GUH or the greater Washington-Baltimore area. MDP-GUH follows approximately 3000 PD patients. Screening for 100 patients will be predominately from our program, however we will advertise to community support groups and websites (Clinicaltrials.gov). Only patients that meet all inclusion/exclusion criteria and are willing to make all visits will be included in the study. Our program has had significant success with recruitment and retention in clinical trials because of the active clinical involvement at GUH and in the satellite centers, including McLean Medical Center, Montgomery Hospital and Washington Hospital Center. Most patients will be local to study center, but any patient who qualifies and can commit to all study visits will be allowed enrollment into the study regardless of

residence. Overall we do not anticipate any issues in recruiting or maintaining 75 patients in this clinical trial for 12-month treatment. We will also advertise in our newsletters and websites.

4.2- Inclusion Criteria

1- Written informed consent
2- Capable of providing informed consent and complying with study procedures. Subjects who are unable to provide consent may use a Legally Authorized Representative (LAR).
3- Patients between the age of 40-90 years, medically stable
4- Diagnosis of PD according to the UK Brain Bank Diagnostic Criteria
5- PD subjects with MoCA ≥ 22
6- 2.5 ≥Hoehn and Yahr stage ≤3
7- MDS-UPDRS-III 20-40
8- No mono-amine oxidase (MAO)-B inhibitors (Selegeline or rasagiline) are allowed at least 6 weeks before enrollment
9- Must be medically stable on ≤800mg Levodopa daily for at least 4 weeks
10-QTc interval 350-460 ms, inclusive
11-Participants must be willing to undergo LP at baseline and 12 months after treatment

4.3- Exclusion Criteria

1. Patients with hypokalemia, hypomagnesaemia, or long QT syndrome- QTc≥461 ms
2. Concomitant drugs known to prolong the QTc interval and history of any cardiovascular disease, including myocardial infraction or cardiac failure, angina, arrhythmia
3. History or presence of cardiac conditions including:
    a. Cardiovascular or cerebrovascular event (e.g. myocardial infarction, unstable angina, or stroke)
    b. Congestive heart failure
    c. First, second- or third-degree atrioventricular block, sick sinus syndrome, or other serious cardiac rhythm disturbances
    d. Any history of Torsade de Pointes
4. Treatment with any of the following drugs at the time of screening or the preceding 30 days, and/or planned use over the course of the trial:
    a. Treatment with Class IA or III antiarrhythmic drugs (e.g. quinidine)
    b. Treatment with QT prolonging drugs (*www.crediblemeds.org)-* excluding Selective Serotonin Reuptake Inhibitors (SSRIs) (e.g. Citalopram, Paxil, Zoloft, Cymbalta, Sertraline, etc...)
    c. Strong CYP3A4 inhibitors (including grapefruit juice). The concomitant use of strong CYP3A4 inhibitors (e.g., ketoconazole, itraconazole, clarithromycin, atazanavir, indinavir, nefazodone, nelfinavir, ritonavir, saquinavir, telithromycin, voriconazole) must be avoided. Grapefruit products may also increase serum concentrations of Nilotinib. Should treatment with any of these agents be required, therapy with Nilotinib should be interrupted.
    d. Anticoagulants, including Coumadin (warfarin), heparin, enoxaparin, daltiparin, xarelto, etc.
    e. St. John's Wort and the concomitant use of strong other CYP3A4 inducers (e.g., dexamethasone, phenytoin, carbamazepine, rifampin, rifabutin, rifapentine, phenobarbital) must be avoided since these agents may reduce the concentration of Nilotinib.
5. Abnormal liver function defined as AST and/or ALT > 100% the upper limit of the normal

6. Renal insufficiency as defined by a serum creatinine > 1.5 times the upper limit of normal
7. History of HIV, clinically significant chronic hepatitis, or other active infection
8. Females must not be lactating, pregnant or with possible pregnancy
9. Medical history of liver or pancreatic disease
10. Clinical signs indicating syndromes other than idiopathic PD, including corticobasal degeneration, supranuclear gaze palsy, multiple system atrophy, chronic traumatic encephalopathy, signs of frontal dementia, history of stroke, head injury or encephalitis, cerebellar signs, early severe autonomic involvement, Babinski sign
11. Current evidence or history in past two years of epilepsy, focal brain lesion, head injury with loss of consciousness or DSM-IV criteria for any major psychiatric disorder including psychosis, major depression, bipolar disorder, alcohol or substance abuse
12. Evidence of any significant clinical disorder or laboratory finding that renders the participant unsuitable for receiving an investigational drug including clinically significant or unstable hematologic, hepatic, cardiovascular, pulmonary, gastrointestinal, endocrine, metabolic, renal or other systemic disease or laboratory abnormality
13. Active neoplastic disease, history of cancer five years prior to screening, including breast cancer (history of skin melanoma or stable prostate cancer are not exclusionary)
14. Contraindications to LP: prior lumbosacral spine surgery, severe degenerative joint disease or deformity of the spine, platelets < 100,000, use of Coumadin/warfarin, or history of a bleeding disorder
15. Must not be on any immunosuppressant medications (e.g. IVig)
16. Must not be enrolled as an active participant in another clinical study
17. Diagnosis of DLB

4.4- Randomization and Registration will be performed by an internet based randomization module). Randomization of the subjects to the 3 treatment groups will be performed in a stratified manner. The chance for randomization to the groups is 1:1:1 for placebo:150 mg:300 mg Nilotinib.

4.5- Blinding. The investigators will be blinded to the dosage. Medications for any patient will be labeled by the CRU with a package medical identification number (Med. Id). A patient specific patient identification number (Pat. Id.) will be assigned to each patient. The investigator will have to note the Pat.Id on the designated medication package number after randomization. Drug dispensation will take place once a month during the monthly visit.

4.6- Un-blinding may occur for emergency purposes in case an AE or SAE makes it necessary for the treating physician to unblind the study treatment– if possible prior contact will be made with the clinical trials co-coordinator (CTC) or project manager. If this is not feasible, the CTC will be contacted within 24 hours after un-blinding. The CTC should not be made aware of what the treatment assignment was. If un-blinding occurs the subject is automatically withdrawn and the procedure for withdrawal will be followed.

4.7- Withdrawal of subjects. In accordance with the Declaration of Helsinki, each subject is free to withdraw from the study at any time without giving reasons for her/his decision. The investigator may also withdraw the subject at any time in the interest of the subject's safety, including severe study-related toxicity as detailed in Table 1 or in the case of un-blinding as described above. The primary reason for withdrawal (e.g. subject wish, safety, withdrawal of consent, etc.) must be recorded in the subject's medical record and on the withdrawal form in the electronic Case Report Form (eCRF). Should

a subject decide to withdraw after administration of study drug, or should the investigator decide to withdraw the subject, all efforts will be made to complete and report the observations up to the time of withdrawal as thoroughly as possible.
A study subject will be discontinued from participation in the study if:

- Any clinical AE, laboratory abnormality, concurrent illness, or other medical condition or situation occurs such that continued participation in the study would not be in the best interest of the subject.
- The participant meets any exclusion criteria (either newly developed or not previously recognized).

Subjects are free to withdraw from participation in the study at any time upon request.

4.8- Handling of Withdrawals
A subject may choose to discontinue participation in the study at any time. Subjects who permanently discontinue study drug should complete early study drug termination procedures per protocol. The subject should then return any unused study drug and will be asked to return to the study site for a final safety visit.

4.9- Termination of Study
This study may be prematurely terminated if, in the opinion of the principal investigator(s) (PIs), there is sufficient reasonable cause.
Circumstances that may warrant termination include, but are not limited to:

- Determination of unexpected, significant, or unacceptable risk to subjects.
- Enrollment is unsatisfactory.
- Insufficient adherence to protocol requirements.
- Data are not sufficiently complete and/or evaluable.

4.10- Protocol Adherence
The Principal Investigator (PI) agrees to adhere to the protocol detailed in this document and agrees that any changes to the protocol must be approved by the site Institutional Review Board (IRB). The PIs will be responsible for enrolling only those study subjects who have met protocol eligibility criteria.

5- SAFETY MONITORING AND REPORTING

The AE definitions and reporting procedures provided in this protocol comply with all applicable regulations and ICH guidelines. The PIs will carefully monitor each subject throughout the study for possible AEs. All AEs will be documented on CRFs designed specifically for this purpose. It is also important to report all AEs, especially those that result in permanent discontinuation of the investigational product being studied, whether serious or non-serious.

5.1- All Non-serious Adverse Events

An AE is any unfavorable and unintended sign (including a clinically significant abnormal laboratory finding, for example), symptom, or disease temporally associated with a study, use of a drug product or device whether or not considered related to the drug product or device.

Adverse drug reactions (ADR) are all noxious and unintended responses to a medicinal product related to any dose. The phrase "responses to a medicinal product" means that a causal relationship between a medicinal product and an adverse event is at least a reasonable possibility, i.e., the relationship cannot be ruled out. Therefore, a subset of AEs can be classified as suspected ADRs, if there is a causal relationship to the medicinal product.

Examples of AEs include: new conditions, worsening of pre-existing conditions, clinically significant abnormal physical examination signs (i.e. skin rash, peripheral edema, etc.), or clinically significant abnormal test results (i.e. lab values or vital signs), with the exception of outcome measure results, which are not being recorded as AEs in this trial (they are being collected, but analyzed separately). Stable chronic conditions (i.e., diabetes, arthritis) that are present prior to the start of the study and do not worsen during the trial are NOT considered AEs. Chronic conditions that occur more frequently (for intermittent conditions) or with greater severity, would be considered as worsened and therefore would be recorded as AEs.

AEs are generally detected in two ways:

Clinical → symptoms reported by the subject or signs detected on examination.

Ancillary Tests → abnormalities of vital signs, laboratory tests, and other diagnostic procedures (other than the outcome measures, the results of which are not being captured as AEs).

If discernible at the time of completing the AE log, a specific disease or syndrome rather than individual associated signs and symptoms should be identified by the PI and recorded on the AE log. However, if an observed or reported sign, symptom, or clinically significant laboratory anomaly is not considered by the Site Investigator to be a component of a specific disease or syndrome, then it should be recorded as a separate AE on the AE log. Clinically significant laboratory abnormalities, such as those that require intervention, are those that are identified as such by the PI.

Subjects will be monitored for AEs from the time they sign consent until completion of their participation in the study (defined as death, consent withdrawal, loss to follow up, and early study termination for other reasons or following completion of the entire study).

5.2- All Serious Adverse Events

An SAE is defined as an adverse event that meets any of the following criteria:

1. Results in death.

2. Is life threatening: that is, poses an immediate risk of death as the event occurred.
    a. This serious criterion applies if the study subject, in the view of the PI or Sponsor, is at immediate risk of death from the AE as it occurs. It does not apply if an AE hypothetically might have caused death if it were more severe.
3. Requires inpatient hospitalization or prolongation of existing hospitalization.
    a. Hospitalization for an elective procedure (including elective PEG tube/g-tube/feeding tube placement) or a routinely scheduled treatment is not an SAE by this criterion because an elective or scheduled "procedure" or a "treatment" is not an untoward medical occurrence.
4. Results in persistent or significant disability or incapacity.
    a. This serious criterion applies if the "disability" caused by the reported AE results in a substantial disruption of the subject's ability to carry out normal life functions.
5. Results in congenital anomaly or birth defect in the offspring of the subject (whether the subject is male or female).
6. Necessitates medical or surgical intervention to preclude permanent impairment of a body function or permanent damage to a body structure.
7. Important medical events that may not result in death, are not life-threatening, or do not require hospitalization may also be considered SAEs when, based upon appropriate medical judgment, they may jeopardize the subject and may require medical or surgical intervention to prevent one of the outcomes listed in this definition. Examples of such medical events include blood dyscrasias or convulsions that do not result in inpatient hospitalization, or the development of drug dependency or drug abuse.

An inpatient hospital admission in the absence of a precipitating, treatment-emergent, clinical AE may meet criteria for "seriousness" but is not an adverse experience, and will therefore, not be considered an SAE. An example of this would include a social admission (subject admitted for other reasons than medical, e.g., lives far from the hospital, has no place to sleep).

The PI is responsible for classifying AEs as serious or non-serious.

5.3- All Reports of Drug Exposure during Pregnancy.

Females, who are lactating/breast feeding, pregnant or with possible pregnancy must not participate in this study. Nilotinib and other drugs and procedures used in this study may be unsafe for a fetus, an infant, sperm and eggs. Women of child bearing potential must agree to avoid pregnancy during participation in this study and for 3 months after the completion of this study. A man must agree not to conceive a child during participation in this study and for 3 months after the completion of this study. If a woman becomes pregnant during the study or if a man fathers a child during the study, they should immediately notify the principal investigator and study coordinator(s) and must be immediately withdrawn from the study.

5.4- All reports of misuse and abuse of Nilotinib and other medication.

Site staff will check the subject's overall compliance with the study requirements. This will also include checks of protocol compliance, Nilotinib and concomitant medication use, in addition to the proper use of study drug in order to assess potential risks of drug abuse or misuse. The PI shall report within 24 hours of discovery any drug misuse experience to the IRB, FDA, Novartis and study monitor.

5.5- Assessment and Recording of Adverse Events

The PIs will carefully monitor each subject throughout the study for possible AEs. All AEs will be documented on CRFs designed specifically for this purpose. All AEs will be collected and reported in

the electronic data capture (EDC) system. The PIs shall promptly review all information relevant to the safety of the investigational product, including all SAEs. Special attention will be paid to those that result in permanent discontinuation of the investigational product being studied, whether serious or non-serious.

5.6- Assessment of Adverse Events
At each visit (including telephone interviews), the subject will be asked if they have had any problems or symptoms since their last visit in order to determine the occurrence of AEs. If the subject reports an AE, the Investigator will probe further to determine:

1. Type of event
2. Date of onset and resolution (duration)
3. Severity (mild, moderate, severe)
4. Seriousness (does the event meet the above definition for an SAE)
5. Causality, relation to investigational product and disease
6. Action taken regarding investigational product
7. Outcome

5.7- Relatedness of Adverse Event to Investigational Product
The relationship of the AE to the investigational product should be specified by the PIs, using the following definitions:

1. Not Related: Concomitant illness, accident or event with no reasonable association with treatment.

2. Unlikely: The reaction has little or no temporal sequence from administration of the investigational product, and/or a more likely alternative etiology exists.

3. Possibly Related: The reaction follows a reasonably temporal sequence from administration of the investigational product and follows a known response pattern to the suspected investigational product; the reaction could have been produced by the investigational product or could have been produced by the subject's clinical state or by other modes of therapy administered to the subject. (Suspected ADR)

4. Probably Related: The reaction follows a reasonably temporal sequence from administration of investigational product; is confirmed by discontinuation of the investigational product or by re-challenge; and cannot be reasonably explained by the known characteristics of the subject's clinical state. (Suspected ADR)

5. Definitely Related: The reaction follows a reasonable temporal sequence from administration of investigational product; that follows a known or expected response pattern to the investigational product; and that is confirmed by improvement on stopping or reducing the dosage of the investigational product, and reappearance of the reaction on repeated exposure. (Suspected ADR)

5.8- Recording of Adverse Events

All clinical AEs are recorded in the AE Log in the subject's study binder. Study staff should fill out the AE Log and enter the AE information into the EDC system within 48 hours of the site learning of a new AE or receiving an update on an existing AE. Entries on GUMC MEDUSA database and the AE Log (and into the EDC) will include the following: name and severity of the event, the date of onset, the date of resolution, relationship to investigational product, action taken, and primary outcome of event. The PI shall report within 24 hours of discovery any serious adverse experience whether or not considered related to the study to the IRB, FDA, Novartis and study monitor who will contact the DSMB. Serious adverse experiences should also be reported immediately by telephone and subsequently in writing within 48 hours of the occurrence to the following:

Robert Schiff, Ph.D., RAC, CQA, FRAPS
Medical Monitor
1120 Bloomfield Avenue
West Caldwell, NJ 07006
Tel: 973-227-1830
Fax: 973-227-5330
Cell: 973-568-3361
Email: *Rschiff13@aol.com*

The Institutional Review Board will be notified of such adverse experiences

The minimum necessary information to be provided at the time of the initial report will include the following:

Study identifier	A description of the event	Whether study was discontinued
Study Center	Date of onset	The reason why the event is classified as serious
Subject number	Current status	Investigator assessment of the association between the event and study treatment

Copies of each report and documentation of IRB notification and receipt will be kept in the Clinical Investigator's study file/site binder.

Adverse Event Reporting Period

The study period during which adverse events must be reported is normally defined as the period from the initiation of any study procedures to the end of the study treatment follow-up

6- STUDY MONITORING

6.1- Safety Monitoring

The study PIs will review safety data throughout the trial and may stop the trial for safety if they determine that there is a significant difference in the rate of a particular AE that would indicate a risk that is greater than the possible benefit of the study drug.

Unanticipated problems involving risks to subjects or others including adverse events will be reported to the Partners Human Research Committee (PHRC) in accordance with PHRC unanticipated problems including AEs reporting guidelines.

6.2- Data Safety Monitoring Board (DSMB) will be formed at the time of the initiation of the study and will include medical experts on Nilotinib effects on cardiac toxicity (Dr. Ana Barac), movement disorders neurologist (Dr. Mark Hallett), a clinical pharmacologist (Dr. Robert Bies), a hematologist and biostatistician (TBD). No investigator involved in the trial will be a member of the DSMB. The DSMB will review the protocol to identify any necessary modifications. If modifications are necessary, revisions will be reviewed by the DSMB prior to its recommendation on initiation of the project. The DSMB, based on its review of the protocol, will identify the data parameters and format of the information to be regularly reported. The DSMB will be informed of the occurrence of any SAEs and immediately notified of fatal or life-threatening events. The DSMB may at any time request additional information from the PIs. The DSMB will be provided with data blinded to treatment status, but they may request un-blinded data if there is a safety concern. Based on the review of safety data, the DSMB will make recommendations regarding the conduct of the study. These may include amending safety monitoring procedures, modifying the protocol or consent, terminating the study or continuing the study as designed. The discussions and decisions of the DSMB will be summarized in written reports and provided to the PIs. The DSMB will meet in person or by conference call on a quarterly basis or as necessary when requested by the PI or the medical monitor.
At the initiation of the study, a DSMB will be immediately formed and an independent study monitor, Dr. Robert Schiff will directly communicate with the DMSB that will create a charter outlining all duties to develop stopping rules for serious and potentially serious irreversible adverse events. The DSMB will also provide boundaries for when to terminate the study or end participation of an individual patient. The stopping rules should include stopping rules as in Table 1 and other criteria related to:
Pancreatitis, QT prolongation (select a method for QT correction (typically Fridericia's method) and include in the protocol), develop individual stopping rules for withdrawal from the study for QT prolongation, and create algorithms for the follow-up of abnormal blood tests that potentially signal the start of a drug induced event (e.g. neutropenia or thrombocytopenia). The DSMB will monitor each patient in real-time and on a case-by-case basis and unblind any case when necessary in consultation with the study monitor.

6.3- Study monitor and auditing will ascertain adherence to the study protocol by investigators and ensure proper documentation and investigator blinding of biomarker data processing and deposition. This study will be monitored by a clinical monitor. The monitors will maintain liaison with the investigators by telephone, letter, email and personal visits in order to assure the sponsors that the clinical study is completed according to the protocol requirements and that Good Clinical Practices are being followed according to 21 CFR parts 50, 56, 812, FDA Guidelines and the ICH E6 Guideline. The PI will allocate adequate time for such monitoring activities. The PI will also ensure that the monitor or other compliance or quality assurance reviewer is given access to all the above noted study-related documents and study related facilities (device testing location), and has adequate space to conduct the monitoring visit.

Auditing inspections will serve to verify strict adherence to the protocol and the accuracy of the data management, in accordance with the federal regulations. The PI will permit study-related monitoring and audits by the IRB, the sponsor, and government regulatory bodies, of all study related documents (e.g. source documents, regulatory documents, data collection instruments, study data etc) at regular intervals throughout the study. The PI will ensure the capability for inspections of applicable study-related facilities (device testing location). Participation as a PI in this study implies acceptance of potential inspection by government regulatory authorities and applicable University compliance and quality assurance offices.
The investigator should be aware that representatives of the FDA might also inspect the study site and subject records. If contacted for an audit of this study by any regulatory agency, the investigator should notify the sponsor immediately.
Study monitor will ascertain adherence to the study protocol by investigators and ensure proper documentation and investigator blinding of biomarker data processing and deposition.

Robert Schiff, Ph.D., RAC, CQA, FRAPS
Medical Monitor
1120 Bloomfield Avenue
West Caldwell, NJ 07006
Tel: 973-227-1830
Fax: 973-227-5330
Cell: 973-568-3361
Email: *Rschiff13@aol.com*

7- Institutional Review Board (IRB)

This study will be conducted in compliance with current Good Clinical Practices (GCP) and Title 21Part 56 of the United States of America Code of Federal Regulations (CFR) relating to IRBs.

7.1- Ethical Conduct of Study

The study will be conducted in accordance with GCP defined by the International Conference on Harmonization (ICH) and the ethical principles of the Declaration of Helsinki.

7.2- Subject Information and Consent

This study will be conducted in compliance with Title 21 Part 50 of the United States of America Code of Federal Regulations (CFR), Federal Regulations and ICH Guidance Documents pertaining to informed consent. At the first visit, prior to initiation of any study-related procedures, subjects will be informed about the nature and purpose of the study, participation/termination conditions, and risks and benefits. Subjects will be given adequate time to ask questions and become familiar with the study prior to providing consent to participate. Subjects will give their written consent to participate in the study and will be provided with a copy of the fully executed consent form for their records.

8- Statistical Methods and Data Analysis

8.1- Sample size determination

Safety: No formal sample-size calculation for 25 participants in each arm was performed. Safety of each dose will be judged by the absence of events of major concern, including QTc prolongation and myelosuppression. According to Novartis Investigator Brochure, the frequency of AEs of all grades leading to discontinuation in the Nilotinib 300mg treatment was 14.0% and the incidence of SAEs in the Nilotinib 300 mg treatment recorded 9%. With 25 participants exposed to each treatment arm, the study will have a 97.7% probability of observing at least one instance of any AE with an expected incidence of at least 14% in both arms when the binomial distribution is assumed. For SAEs, this probability of at least one event will decrease to 94.6% with an expected incidence of at least 9% in both arms.

Tolerability: If we define a dose as tolerable when we observe no more than 25% intolerance or the discontinuation ratio is greater than 25%, the probability of early discontinuation of this trial (>25%), i.e., observing 8 or more AEs out of 25 is 1.8% (1-98.2%) given 25 participants in Nilotinib 300mg Arm. If we assume 9% of probability of SAEs occurrence, the probability of discontinuation due to SAEs of this trial, i.e., observing 8 or more severe AEs out of 25 is 0.1% (1-99.9%). We expect that the probabilities of discontinuation due to drug-related AEs or SAEs in the 150mg treatment group will be at most the discontinuation probability in Nilotinib 300mg Arm.

Primary biomarkers. Given sample size of 25 per arm, we calculate powers to Nilotinib effects in terms of primary biomarkers between baseline and 12 months. Given a primary biomarker, we will test the null hypothesis that the biomarker change in the control group (Arm I) over 12 months equal the biomarker change in the treatment group of Arm II (150mg) or Arm III (300mg) using a two-sided significance level 0.05, respectively.
It is assumed that 10% complete losses and additional 20% partial loss of information due to symptomatic treatment leads to ``drop-out'' rate of 30%. Considering 30% drop-out, a total of 51 patients (17 patients in each arm) will be considered. Given observed statistics on biomarker changes in the pilot study, we anticipate powers of each biomarker analysis as stated in Table 10.

Table 4- Estimated power based on CSF HVA biomarkers

Arm	Biomarker	Placebo	Treatment	Difference	SD	Power (N=25)	Power (N=17)
Arm II (150mg) vs Arm I (placebo)	HVA	0%	50%	50%	50%	94%	83%
Arm III (300mg) vs Arm I (placebo)	HVA	0%	50%	50%	40%	99%	95%

We will have at least 83% power to test our null hypotheses for HVA biomarkers based on comparing means using a normal distribution. Given the same set-up in the Table 5, a number of 25 patients will achieve at least 68% power at a two-sided at 0.05 for testing clinical outcomes (MDS- UPDRS). Due

to normality given a small sample size, a simulation based non-parametric Mann-Whitney-Wilcoxon test (MWW) power is also computed given the mean change rate and SD. For HVA, MWW power is >0.77 with 17 patients at each arm and MWW power for UPDRS >0.62 with 17 patients at each arm.

Table 4.1- Estimated power based on two types of UPDRS

Arm	Clinical outcomes	Placebo	Treatment	Difference	SD	Power (N=25)	Power (N=17)
Arm II vs Arm I	UPDRS I-IV	3	-7	10	12	84%	68%
Arm III vs Arm I	UPDRS I-IV	3	-11	14	8	100%	100%

<u>8.2- Analyses plan</u>:

Safety: The frequency of AEs classified by MedDRA system organ class and preferred term and clinically significant changes in EKG and laboratory parameters will be summarized as simple proportions with exact 95% confidence bounds. The proportion of participants experiencing each type of event will be compared by Fisher's exact test.

Tolerance: The proportion tolerant of each dose will be estimated with exact confidence intervals.

Target Engagement: The measurements of biomarkers at baseline and the change from baseline to 6 months will be summarized in terms of mean and standard deviation. To compare efficacy of the two doses of Nilotinib, 150 mg and 300 mg per day, a shared-baseline repeated-measures ANOVA will be used with estimates of treatment-specific changes over 6 months and differences between treatments obtained from linear contrasts. This model will include fixed effects of visit (baseline and 12 months) and a dose by post-baseline visit interaction and unstructured covariance for the repeated measurements. One additional baseline covariate may be included if a large difference in a potentially prognostic measure results by chance. A significant dose effect will be judged based on two-tailed testing at a p-value of 0.05. Other CSF and plasma exploratory biomarkers, and clinical outcomes will be analyzed by the same model. The relationship between 12-month changes in Abl activity or other biomarkers and 12-month changes in clinical outcomes will be summarized as simple correlations with a visual verification that correlations within each dose roughly match correlations across doses, i.e., absence of Simpson's paradox.

9- STUDY FEASIBILITY AND PRELIMINARY DATA

9.1- Preclinical Evidence

We previously demonstrated that Nilotinib penetrates the brain, inhibits Abl activity and promotes autophagic degradation of amyloid proteins in transgenic mice (13,23,24,26,45). These findings were also reproduced by several other laboratories (13,35,36). To determine whether Nilotinib enters the brain, male and female 2 months old wild type (WT) C57BL/6 mice were intraperitoneally (I.P.) injected with an escalating dose of 1, 10, 20, 40 and 80 mg/kg Nilotinib dissolved in 30µL DMSO (n=3 per group) and sacrificed at 2, 4, 6, 8 and 12hr post-injection (n=3 per time point). The concentration of Nilotinib was measured by mass spectrometry using ^{13}C labeled Nilotinib as a standard control in the plasma (Fig. 1A) and total brain homogenates (Fig. 1B). At 10 mg/kg Nilotinib reached the highest brain/blood ratio (5.08%) at 305nM peak (T_{max}) 4hr post-injection; and 1 mg/Kg brain/blood was 3.06% at 205nM (Fig. 1C). Higher doses of Nilotinib (20, 40 and 80mg/kg) injection did not result in higher brain levels, perhaps due to the role of the ATP-binding cassette (ABC) transporters, which facilitate Nilotinib efflux at the BBB (45).

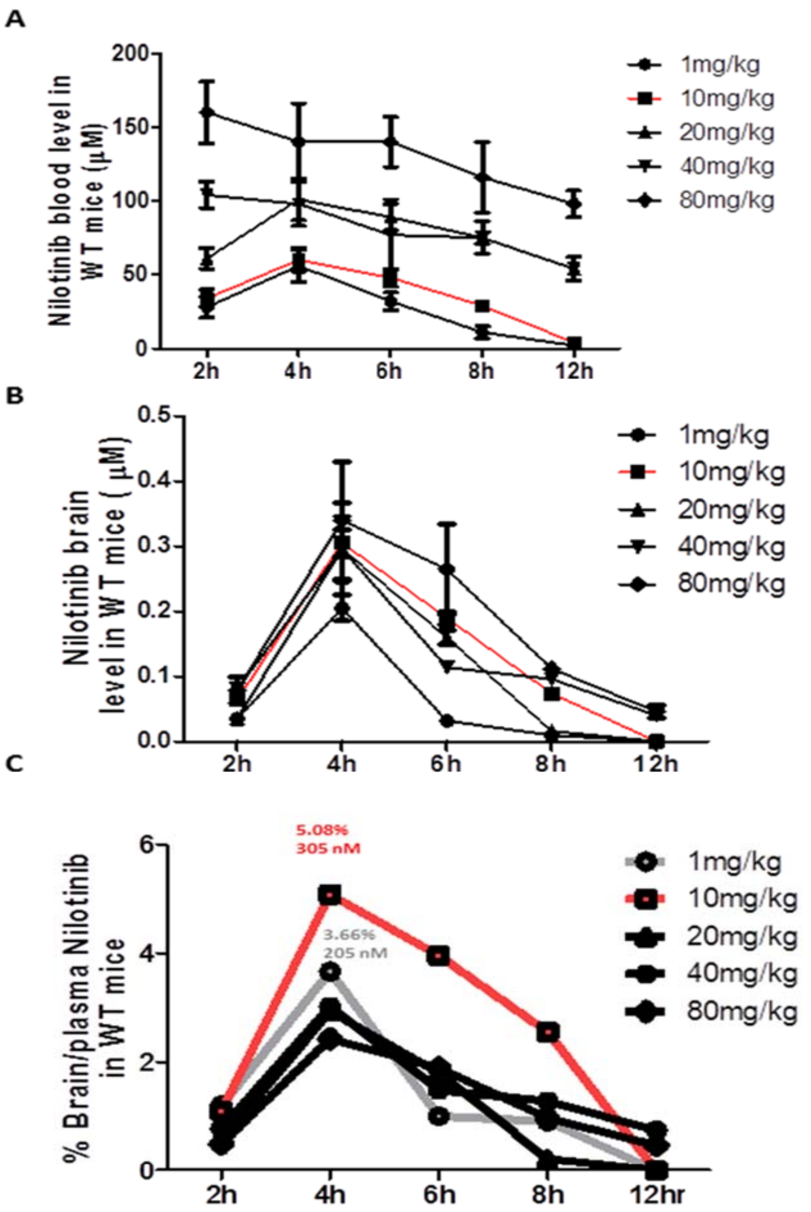

Figure 1. Pharmacokinetics analysis showing Nilotinib concentration in A) plasma, B) brain and C) percentage brain/plasma

Fig. 1- Abl activation is associated with accumulation of alpha-Synuclein. WB on 10% SDS-NuPAGE gel shows **A**) Lentiviral alpha-Synuclein expression (1st blot), total Abl (2nd blot) and tyrosine 412 (T412) phosphorylated Abl (3rd blot) relative to actin (N=9), and **B**) graphs represent densitometry analysis. **C**) Total Abl (1st blot) and tyrosine 412 (T412) phosphorylated Abl (2nd blot), and mouse alpha-Synuclein expression (3rd blot) relative to actin (N=9) in lentiviral Abl and LacZ injected mice, and **D**) graphs represent densitometry analysis. IHC in 20 µm thick brain sections showing **E**) V5, **F**) Abl and **G**) merged V5 and Abl staining in the SN of mice injected with lentiviral Abl. IHC in 20 µm thick brain sections showing **H**) V5, **I**) Abl and **J**) merged V5 and Abl staining in the SN of mice injected with lentiviral LacZ. WB on 4-12% SDS-NuPAGE gel shows **K**) total Abl (1st blot) and T412 Abl (2nd blot) relative to actin in human post-mortem striatal extracts, N= 9 PD and 7 controls, $p<0.02$, two-tailed t-test, and **L**) Densitometry of human WBs. **M**) Graph represents quantification of Mass Spectroscopy analysis of brain Nilotinib (N=5/time point). **N**). WB on 4-12% SDS-NuPAGE gel shows total Abl (1st blot), T412 Abl (2nd blot) T245 Abl (3rd blot) and phospho-tyrosine (4th blot) relative to parkin in wild type mice injected with DMSO or Nilotinib once daily for 3 weeks and **O**). Graphs represent densitometry analysis. *Significantly different, ANOVA, Neumann Keuls multiple comparison, $p<0.05$. N= number of animals, and bars are means.

Abl activation is associated with accumulation of alpha-Synuclein. To examine the relationship between Abl and alpha-Synuclein, male C57BL/6 mice were stereotaxically injected with 1x10^4 multiplicity of infection (m.o.i) lentiviral clones driving the expression of Abl, or alpha-Synuclein (or LacZ) bilaterally into the substantia nigra (SN). Lentiviral injection significantly increased alpha-Synuclein (42%) over LacZ level (Fig. 2A&B, 1st blot, N=9 animals) 6 weeks post-injection. Alpha-Synuclein expression led to an increase in total Abl (110%) relative to actin and tyrosine 412 (T412) phosphorylation (289%) relative to total Abl (Fig. 2A&B, p<0.05, N=9) compared to LacZ expressing mice, indicating Abl activation. Conversely, lentiviral expression of Abl in the mouse SN led to an increase (204%) in total Abl relative to actin (Fig. 2D&E, p<0.05, N=9) and T412 phosphorylation (231%) relative to total <u>Abl and resulted in increased levels of monomeric (51%) and high molecular weight alpha-Synuclein</u> (relative to actin) 6 weeks post-injection

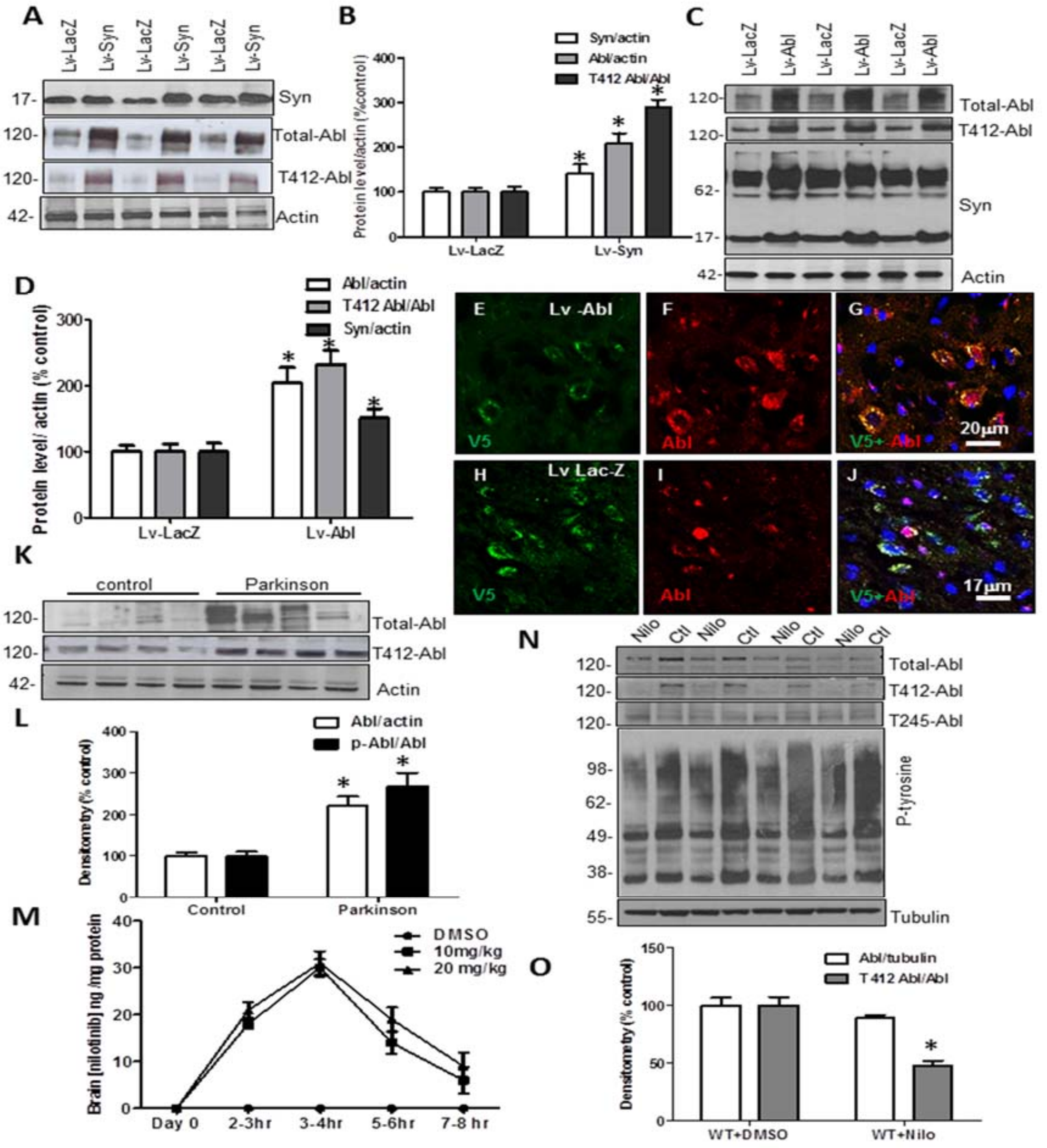

Fig. 2- Abl activation is associated with accumulation of alpha-Synuclein. Lentiviral alpha-Synuclein expression (1st blot), total Abl (2nd blot) and tyrosine 412 (T412) phosphorylated Abl (3rd blot) relative to actin (N=9), and **B**) graphs represent densitometry analysis. **C**) Total Abl (1st blot) and tyrosine 412 (T412) phosphorylated Abl (2nd blot), and mouse alpha-Synuclein expression (3rd blot) relative to actin (N=9) in lentiviral Abl and LacZ injected mice, and **D**) graphs represent densitometry analysis. IHC in 20 µm thick brain sections showing **E**) V5, **F**) Abl and **G**) merged V5 and Abl staining in the SN of mice injected with lentiviral Abl. IHC in 20 µm thick brain sections showing **H**) V5, **I**) Abl and **J**) merged V5 and Abl staining in the SN of mice injected with lentiviral LacZ. WB on 4-12% SDS-NuPAGE gel shows **K**) total Abl (1st blot) and T412 Abl (2nd blot) relative to actin in human post-mortem striatal extracts, N= 9 PD and 7 controls, p<0.02, two-tailed t-test, and **L**) Densitometry of human WBs. **M**) Graph represents quantification of Mass Spectroscopy analysis of brain Nilotinib (N=5/time point). **N**). WB on 4-12% SDS-NuPAGE gel shows total Abl (1st blot), T412 Abl (2nd blot) T245 Abl (3rd blot) and phospho-tyrosine (4th blot) relative to parkin in wild type mice injected with DMSO or Nilotinib once daily for 3 weeks and **O**). Graphs represent densitometry analysis. *Significantly different, ANOVA, Neumann Keuls multiple comparison, p<0.05. N= number of animals, and bars are means.

compared to Lac-Z. To verify that both lentiviral LacZ and Abl were expressed, a V5 tag of the lentiviruses were probed with IHC. Staining of 20μm thick SN sections showed V5 (Fig. 2E) and Abl (Fig. 2F) co-localized (Fig. 2G) in lentiviral-Abl injected brains, while staining of Lac-Z injected SN showed V5 (Fig. 2H) and endogenous Abl (Fig. 2I) without co-localization (Fig. 2J), indicating lentiviral expression.

Western Blot (WB) was performed on homogenized frozen striatal (caudate) brain tissues (described in (12)) from 9 sporadic PD patients and 7 age-matched control subjects. Human post-mortem PD striatal extracts showed an increase in total Abl (220%) relative to actin and T412 (267%) relative to total Abl (Fig. 2K&L) compared to control subjects ($p<0.02$, two-tailed t-test), suggesting a relationship between Abl activation and alpha-Synuclein in PD.

Intraperitoneal (I.P.) injection of 10-20 mg/kg Nilotinib into WT mice (N=5 animals/time point), led to detection of up to 30ng Nilotinib (310nM) per mg brain tissue 3-4hr after injection (Fig. 2M) and Nilotinib was still detectable at 3.4ng/mg (35nM) 7-8 hr., indicating that Nilotinib enters the brain and is washed out after several hours. Two months old C57BL6 mice were I.P. injected once a day with 10mg/kg Nilotinib or DMSO (30μL) for 3 consecutive weeks, and total brain extracts were analyzed with WB. Nilotinib led to a slight (11%) decrease in total Abl relative to tubulin but T412 Abl was significantly decreased (52%) relative to total Abl compared to DMSO (Fig. 2N&O, $p<0.05$, N=10 animals). No

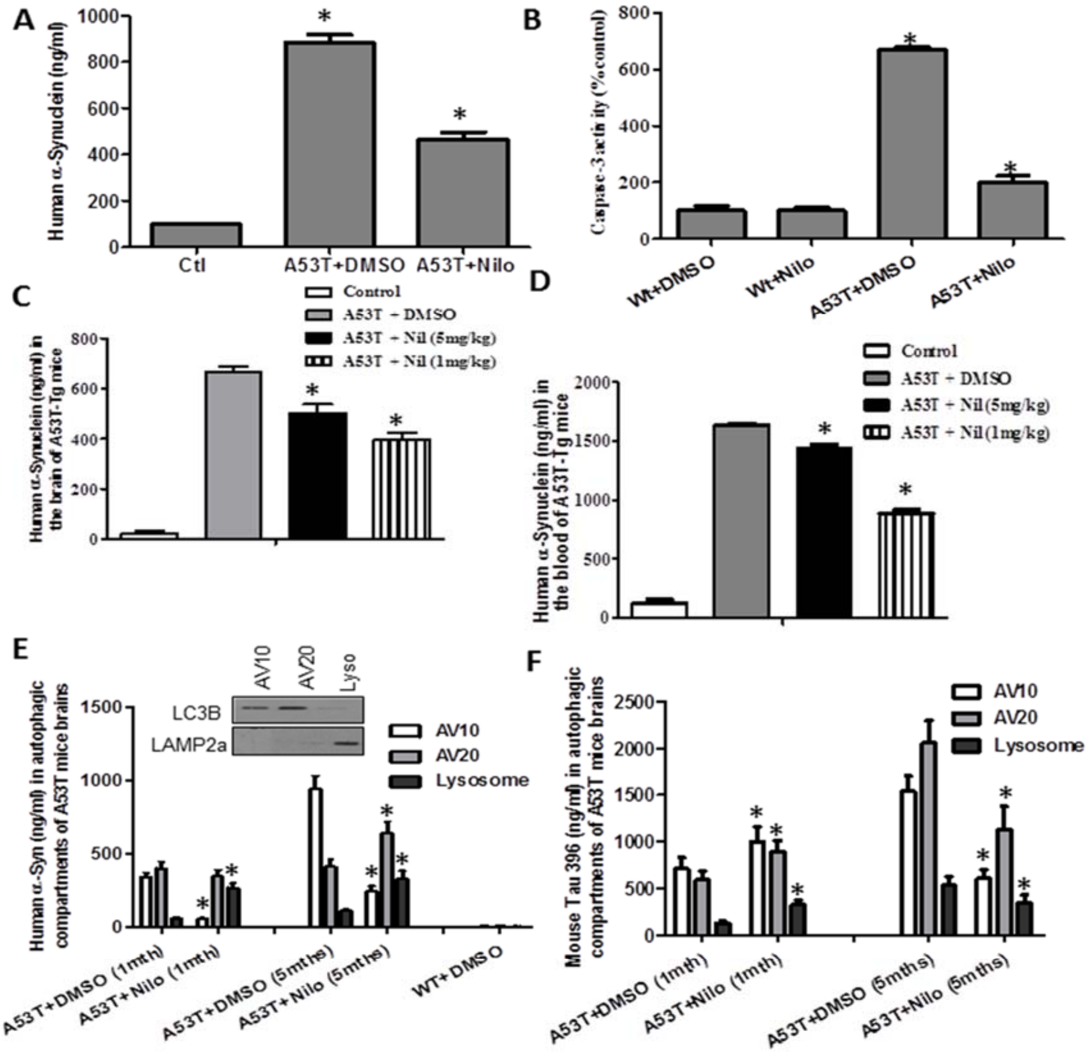

Fig. 3- Abl inhibition via Nilotinib promotes autophagic degradation. ELISA measurement of **A)** human alpha-Synuclein (N=14) and **B)** caspase-3 activity (N=64) in 6-8 months old transgenic A53T mice and wild type age-matched controls injected daily IP with 10mg/kg Nilotinib for 3 weeks. Graphs represent ELISA measurement of **C)** brain levels of human alpha-Synuclein (N=10) and **D)** blood levels of human alpha-Synuclein (N=10) in 5 months old transgenic A53T mice and wild type age-matched controls treated IP with 5mg/kg or 1mg/kg Nilotinib every other day for 6 weeks. Graphs represent ELISA measurement of **E)** human alpha-Synuclein (insert shows WB of AVs) and **F)** p-Tau levels in A53T mice (N=5) treated daily IP with 10mg/kg Nilotinib for 3 weeks. *Significantly different, ANOVA, Neumann Keuls multiple comparison, p<0.05. N=number of animals, bars are means.

changes were detected in T245 Abl relative to actin or total Abl (Fig. 2N, 3rd blot). These data were confirmed by other groups (13,35,36).

Nilotinib decreases brain and blood alpha-Synuclein levels. Nilotinib effects on alpha-Synuclein levels were tested in 7-8 months old transgenic alpha-Synuclein mice that harbor the A53T mutation of alpha-Synuclein (46). Total brain lysates showed accumulation (ELISA) of up to 885ng/ml human alpha-Synuclein (Fig. 3A) in 7-8 months mice treated with DMSO compared to wild type control ($p<0.05$, N=10), but daily IP injection of 10mg/kg Nilotinib for 3 weeks significantly decreased alpha-Synuclein to 467ng/ml ($p<0.05$, N=10), indicating that once a day treatment with Nilotinib decreases brain alpha-Synuclein levels. A significant increase in caspase-3 activity (Fig. 3B, 670%, $p<0.05$, N=64), indicating apoptotic death, was observed in A53T mice brains and Nilotinib reversed this increase to 201% compared to wild type age-matched controls with and without Nilotinib (N=64). The

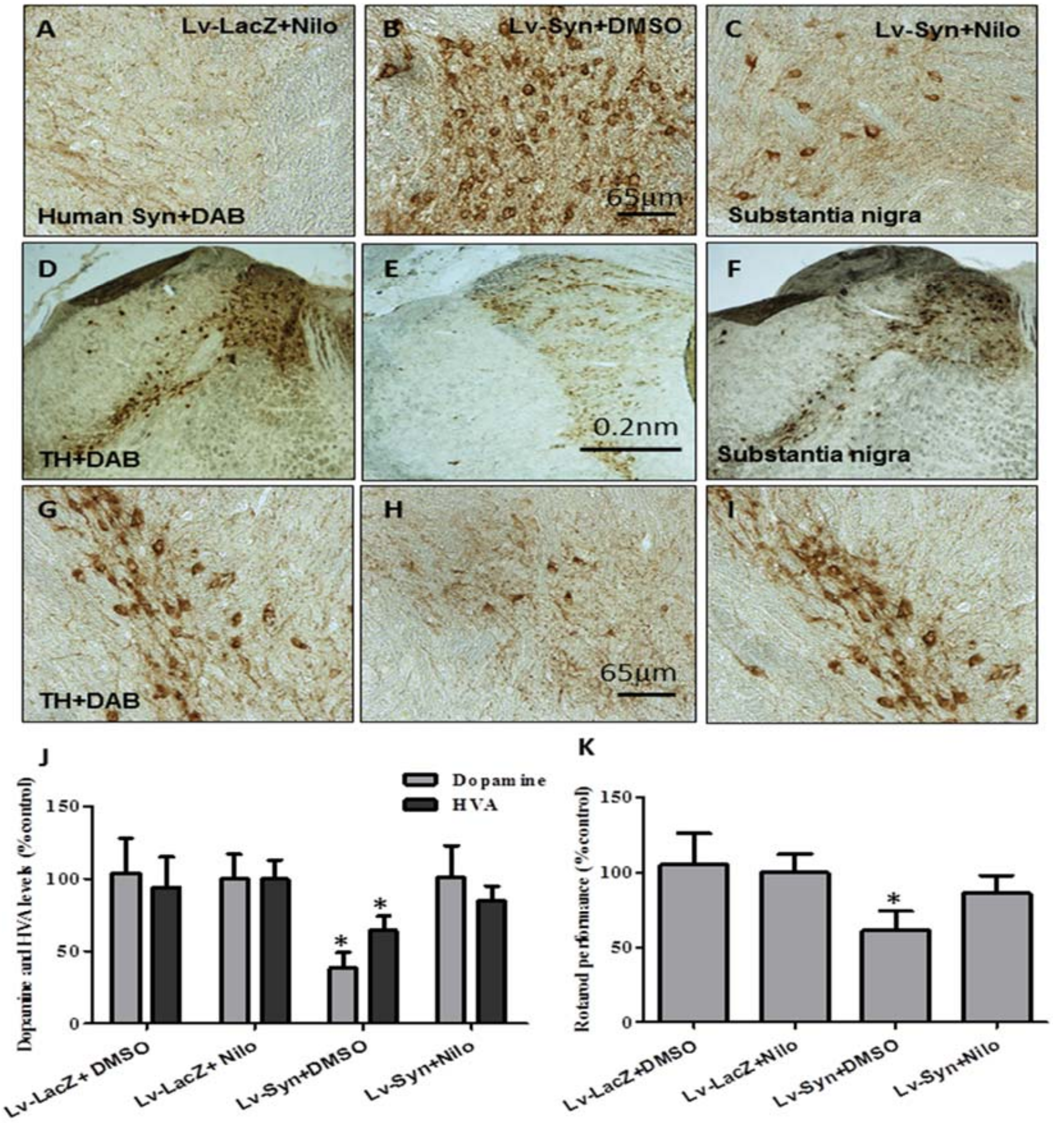

Fig. 4- Nilotinib protects dopamine neurons and improves motor. Staining of 20μM thick brain sections shows human alpha-Synuclein in the SN of **A**) lentiviral LacZ injected mice treated with 10mg/kg Nilotinib, **B**) lentiviral alpha-Synuclein treated with DMSO and **C**). lentiviral alpha-Synuclein treated with 10mg/Kg Nilotinib daily for 3 weeks. Staining of 20 µm thick brain sections show TH in **D**). lentiviral LacZ injected with Nilotinib, and **G**) is higher magnification from a different animal, **E**) lentiviral alpha-Synuclein and treated with DMSO, and **H**) is higher magnification from a different animal. **F**). lentiviral alpha-Synuclein treated with Nilotinib, and **I**) is higher magnification from a different animal. Graphs represent concentrations of **J**). DA and HVA ELISA levels in mesencephalon brain extracts of lentiviral alpha-Synuclein treated with DMSO compared to LacZ mice (N=8) and **K**). shows time spent (%) on rotarod in lentiviral a-Synuclein or LacZ injected mice with and without Nilotinib (N=14). *Significantly different, ANOVA, Neumann Keuls multiple comparison, $p<0.05$. N= number of animals, Bars are means.

effects of lower dose and longer periods of treatment were evaluated in the blood and brain of 5-6 months old A53T mice, which were injected every other day with 1mg/kg or 5mg/kg Nilotinib for 6 weeks (compared to 3 weeks). Whole blood was collected via cardiac puncture and brain tissues were extracted in lysis buffer and analyzed by ELISA. A total of 665ng/ml human alpha-Synuclein was observed in the brain of A53T treated with DMSO compared to C57BL/6 age-matched control (Fig. 3C, $p<0.05$, N=10), but alpha-Synuclein levels decreased to 503ng/ml with 5mg/kg and 344ng/ml with 1mg/kg Nilotinib for 6 weeks. A higher concentration of human alpha-Synuclein (1635ng/ml) was detected in the whole blood of A53T mice treated with DMSO compared to C57BL/6 age-matched control (Fig. 3D, $p<0.05$, N=10), and, again, alpha-Synuclein levels decreased to 1439ng/ml with 5mg/kg and 888ng/ml with 1mg/kg Nilotinib for 6 weeks, suggesting that the decrease in brain levels may also decrease blood alpha-Synuclein. Daily IP injection of 10mg/kg Nilotinib for 3 weeks into 7-8 months A53T mice again decreased monomeric (41%) and high molecular weight human alpha-Synuclein (13) relative to actin compared to A53T mice treated with DMSO.

To ascertain that autophagy is involved in Nilotinib-mediated alpha-Synuclein clearance in A53T mice *in vivo*, autophagic vacuoles (AVs) were isolated via subcellular fractionation using a discontinuous Metrizamide gradient (47) and the levels of alpha-Synuclein and p-Tau were measured via ELISA. Subcellular fractionation was performed in an age-dependent manner to determine whether higher levels of protein accumulation alter autophagic flux through the AV10 or AV20 Metrizamide gradients, which contain LC3B (Fig. 3E, inset), indicating phagophore/autophagosome presence and the lysosomal fraction containing lysosomal associated membrane protein (LAMP)-2a. Human alpha-Synuclein was detected in AV10 (340ng/ml) and AV20 (401ng/ml) in 1 month old A53T brains (Fig. 3E, $p<0.05$, N=5) but 10mg/kg Nilotinib significantly decreased alpha-Synuclein levels (56ng/ml) in AV10 (Fig. 3E, $p<0.05$, N=5). However, Nilotinib significantly increased alpha-Synuclein levels in the lysosomes (268ng/ml) compared to DMSO (59ng/ml). Alpha-Synuclein was even higher in AV10 (940ng/ml) and AV20 (410ng/ml) in 5 months old A53T mice treated with DMSO (Fig. 3G, $p<0.05$, N=5) but alpha-Synuclein levels were decreased by Nilotinib in AV10 (245ng/ml) and increased in AV20 (642ng/ml) compared to DMSO. Nilotinib also significantly increased alpha-Synuclein levels in the lysosomes (333ng/ml) compared to DMSO (109ng/ml) in the same age group. P-Tau was also used as another protein marker that can potentially be degraded by autophagy. P-Tau was detected in AV10 (710ng/ml), AV20 (590ng/ml) and lysosomes (129ng/ml) in 1 month old A53T mouse brain (Fig. 3F, $p<0.05$, N=5), indicating Tau hyper-phosphorylation. However, 10mg/kg Nilotinib increased p-Tau in AV10 (1001ng/ml), AV20 (890ng/ml) and lysosomes (321ng/ml) compared to DMSO within the same age group (Fig. 3F, $p<0.05$, N=5). p-Tau was higher in AV10 (1540ng/ml) and AV20 (2055ng/ml) in 5 months old A53T mice treated with DMSO (Fig. 3F, $p<0.05$, N=5) but Nilotinib decreased p-Tau in AV10 (610ng/ml) and increased it in AV20 (1133ng/ml) compared to DMSO. Nilotinib also decreased p-Tau in the lysosomes (345ng/ml) compared to DMSO (530ng/ml).

These data suggest that Nilotinib inhibition of Abl triggers autophagic protein clearance in vivo.

Nilotinib protects SN tyrosine hydroxylase (TH) neurons from alpha-Synuclein toxicity. Staining of 20μm thick brain sections showed human alpha-Synuclein expression in mice injected with lentiviral alpha-Synuclein into the SN and treated with DMSO (Fig. 4B) compared to LacZ treated with Nilotinib (or DMSO data not shown) mice (Fig. 4A, N=12); but Nilotinib led to 84% (by stereology)

decrease of human alpha-Synuclein (Fig. 4C, $p<0.05$, N=12) in SN neurons. A significant decrease in TH^+ neurons (89% by stereology) was observed in lentiviral alpha-Synuclein treated with DMSO (Fig. 4E&H) compared to LacZ treated with Nilotinib (Fig. 4D&G), and Nilotinib treatment of alpha-Synuclein expressing mice reversed TH^+ neuron loss back to 82% (Fig. 4F&I, by stereology) of LacZ level ($p<0.05$, N=12). To evaluate alpha-Synuclein and Nilotinib effects on dopamine (DA) metabolism, DA and its metabolite Homovanilic acid (HVA) in SN brain extracts were measured using ELISA. A significant decrease ($p<0.05$, N=8) in DA (62%) and HVA (36%) were observed in SN extracts of lentiviral alpha-Synuclein treated with DMSO compared to LacZ with and without Nilotinib (Fig. 4J). However, Nilotinib significantly ($P<0.05$, N=8) reversed DA and HVA loss back to control (Fig. 4J, N=8). Alpha-Synuclein expression in SN decreased rotarod performance 39% (Fig. 4K, $p<0.05$, N=14) of LacZ with and without Nilotinib, but Nilotinib reversed motor performance to 86% of LacZ (Fig. 4K, $p<0.05$, N=14).

9.2- Human Studies

To examine the effects of lower doses of Nilotinib on safety and efficacy in PDD and PD with MCI and DLB, twelve late stage (Hoehn and Yahr 3-5) PDD and DLB participants were randomized into 150mg (N=5) or 300mg (N=7) groups and received oral daily doses of Nilotinib for 6 months with 3-month follow up (Chart 3 and Table 6). The primary objective was to determine the safety and tolerability of Nilotinib for 6 months throughout a total of 11 visits. Electrocardiograms (EKG), physical and neurological exams and blood draws for laboratory chemistry were performed every 2 weeks for the first 2 months, and every month thereafter. Participants were on stable medical conditions on L-Dopa treatment and other PD medications for 4 weeks prior to study enrollment as indicated in Table 6. Patients with cardiovascular complications, infarcts, QTc interval >450ms and those who are on drugs that prolong QTc or with potential drug contraindications were excluded as per package insert. Thirty patients were screened and 12 enrolled. The secondary objectives determined whether Nilotinib

Chart 4- Standard CONSORT flow diagram for the trial

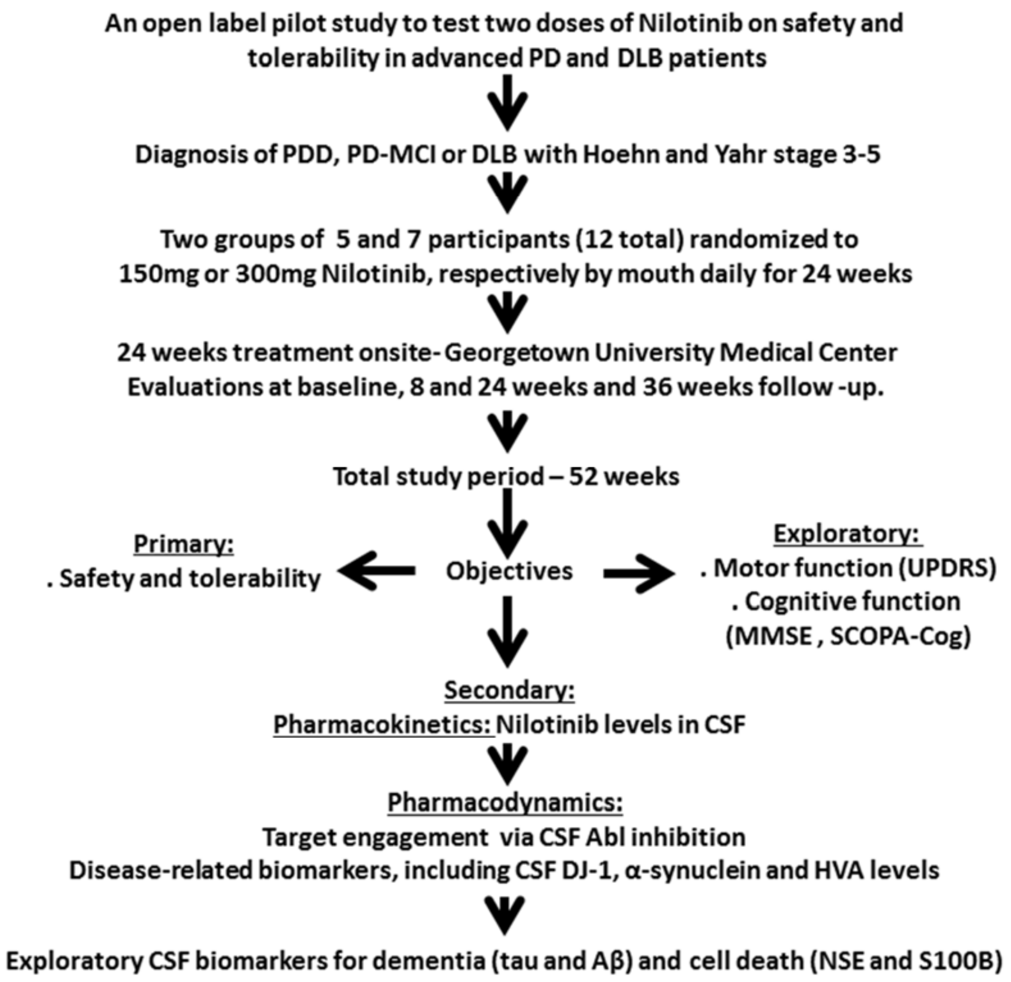

crosses the BBB. Nilotinib concentration was measured in plasma and CSF at baseline, 2 months (interim) and 6 months. Pharmacodynamics analysis was performed to determine target engagement via measurement of CSF phosphorylated Abl (activity) in addition to neurodegeneration biomarkers,

including alpha-Synuclein, dopamine metabolite HVA, total Tau and p-Tau181/231, Aβ42 and Aβ40. Cell death markers, including CSF NSE and glial and neuronal marker S100B were measured. Tertiary/exploratory outcomes included measurement of motor and non-motor symptoms using UPDRS I-IV. Montreal Cognitive Assessment (MoCA) in the range of 18-26 was used at screening to determine mild cognitive impairment (MCI), moderate cognitive impairment (17-10) and severe cognitive impairment (<10) in PD and LBD patients. Nilotinib effects on progression of dementia were evaluated

Table 6- Demographics summary of all participants, including 18 "screen fail" and 12 enrollment. PD: Parkinson's disease; DLB: dementia with Lewy body; MCI: mild cognitive impairment; PDD: Parkinson's disease with dementia; MoCA: Montreal Cognitive Assessement.

Total screened	30											
Total enrolled	12											
Total withdrawal due to adverse events	1											
Voluntary discontinuation	1											
Study duration	24 weeks											
Nilotinib study group (mg)	150	150	150	150	150	300	300	300	300	300	300	300
	NIL-01	NIL-05	NIL- 08	NIL-14	NIL-11	NIL-06	NIL-09	NIL-12	NIL-13	NIL-03	NIL-15	NIL-02
Age	69	72	73	74	74	67	81	49	65	74	89	75
Gender	M	M	F	M	M	M	M	M	M	F	F	M
Weight (kgs.)	60.1	71.9	52.3	63.2	76	83.2	78.9	110.2	54.5	69.8	46.7	75.7
Height (cm)	165.8	170.2	142	166.6	175.2	175.1	178.4	170.4	177	168.1	160	178.2
BMI	21.9	24.8	25.9	22.8	24.8	27.1	24.8	38	17.4	24.7	18.2	23.8
Levodopa at baseline	450	500	750	400	1000	500	400	550	500	1150	1160	950
Levodopa at week 24	450	500	650	400	750	500	400	550	500	1025	1160	500
Azilect at baseline	0.5mg	0.5mg	0.5mg	none	1mg	1mg	1mg	1mg	1mg	1mg	1mg	0.5mg
Azilect at week 24	none	0.5mg	none	none	1mg	0.5mg	1mg	none	0.5mg	none	0.5mg	none
Years since diagnosis	10	9	13	3	18	13	20	15	8	11	13	7
Race	Caucasian	Caucasian	Caucasian	Caucasian	Caucasian	Other	Asian	Caucasian	Caucasian	Caucasian	Caucasian	Other
Diagnosis	DLB	DLB	DLB	DLB	PD-MCI	PDD	PD-MCI	PD, MCI	DLB	PD	PDD	PD-MCI
MoCA	11	Unable	9	None	21	14	19	23	22	28	16	22
Stage	Stage 3	Stage 5	Stage 5	Stage 3	Stage 3	Stage 3	Stage 4	Stage 3	Stage 5	Stage 3	Stage 5	Stage 3

using the MMSE and SCOPA-Cog.

9.2.a- Pharmacokinetic studies

Blood and CSF were collected from twelve patients at baseline prior to treatment (N=12, control) and 2 months into treatment (N=12). Blood and CSF were collected from nine patients at six months (N=9) of Nilotinib treatment (total N=33 samples) and collection time via lumbar puncture (LP) was staggered at 0, 1, 2, 3, 4 or 5hrs after oral administration of Nilotinib. Blood was collected half an hour prior to LP. Two CSF samples were eliminated because they had >25ng/ml hemoglobin.

Figure 2

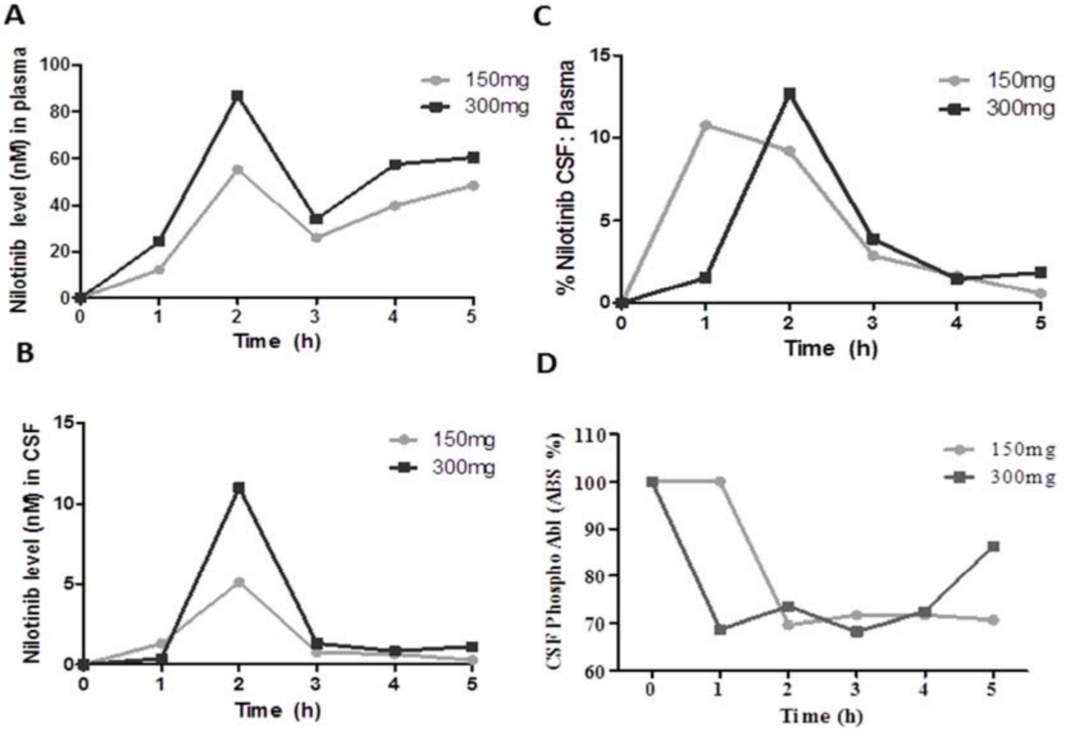

E

Noncompartmental anaylsis		
CSF	150mg	300mg
Tmax (h)	2	2
Cmax (nM)	5.09	11.01
AUC ng/ml*h	7.98	14.13
t1/2	1.854	1.735
Plasma	150mg	300mg
Tmax (h)	2	2
Cmax (nM)	55.27	86.75
AUC ng/ml*h	157.27	232.8
t1/2	12.4	12.4

Fig. 5- Pharmacokinetic analysis showing A) Nilotinib concentration (T_{max}=2hrs) in human brain. Pharmacological paramaters after treatment with A1) 150 mg and A2) 300 mg Nilotinib. Pharmacodynamics of Nilotinib B) showing direct target engagement via inhibition of CSF Abl.

Pharmacokinetic analysis demonstrates that Nilotinib (Fig. 5A&E) peaks in the plasma 2hrs (T_{max}) after dosing at 55.27nM and 86.75nM (C_{max}) in the 150mg and 300mg groups, respectively. Nilotinib (Fig. 5B&E) also peaks in the CSF 2hrs (T_{max}) after dosing at 5.09nM and 11.1nM (C_{max}) in the 150mg and 300mg groups, respectively. Area under the curve (AUC) is 157.27 and 232.8 ng/ml*h for plasma and 7.98 and 14.13 ng/ml*h for CSF in 150mg and 300mg groups, respectively. Nilotinib was quantified in reference to ^{13}C Nilotinib standard and samples were precipitated and dialyzed to obtain unbound Nilotinib. Nilotinib was not detected in the CSF (t1/2 ~1.8) 3hrs after dosing suggesting short bioavailability of unbound or free Nilotinib compared to plasma t1/2=12.4 (Fig. 5E). The CSF:plasma ratio of Nilotinib (Fig. 5C) is 12% and 5% with 300mg and 150mg Nilotinib, respectively.

9.2.b- Pharmacodynamics. To demonstrate whether Nilotinib inhibits Abl via de-phosphorylation, we measured pan-tyrosine CSF Abl relative to total Abl via ELISA. Phosphorylated Abl was reduced with 150mg and 300mg Nilotinib (Fig. 5B), suggesting that Nilotinib (~12% -22%) decreases CSF Abl (>30%) phosphorylation (inhibition). These data support the pre-clinical results that Nilotinib penetrates the brain. The differences in T_{max} and C_{max} between human and mice can be attributed to several factors, including route of administration (i.e. I.P-mouse versus oral-

human), methods of dissolving the drug (DMSO in mice), integrity of BBB in advanced PDD and DLB, differences in drug metabolism between mice and human, concomitant medication and diet. Additionally, Nilotinib was quantified in whole brain extracts in mice versus human CSF, where drug-protein binding is likely to be different. Collectively these data support further investigation to determine Nilotinib concentration in CSF in larger trials with early PD.

9.3- Safety

9.3.1- Serious Adverse Events (SAEs). Adverse Events (AEs) that required or resulted in hospitalization were classified as SAEs and were listed in Table 7. One patient in the 300 mg Nilotinib group (NIL-9) was withdrawn at week 4 due to moderate cardiac ischemia and myocardial infarct and was double-stented for left bundle block. One participant in the 300mg Nilotinib group was hospitalized with urinary tract infection (UTI) and one participant in the 150mg group was hospitalized with pneumonia and UTI (Table 7).

Table 7- Serious Adverse Events (SAEs) requiring hospitalization in all [illegible]

System Organ Class/Preferred Term	Number of affected participants N=12	Total number of events	Numb1er of affected participants in 150mg group. N=5	Number of affected participants in 300mg group. N=7
Cardiac disorders	1	2	0	1
Moderate cardiac ischemia			0	1
Myocardial Infarct			0	1
Infections and infestations	2	3	1	1
Urinary tract infection			1	1
Pneumonia			1	

9.3.2- Non-serious AEs. Non-serious AEs, which did not require hospitalization were classified per system and/or preferred term and summarized in Table 8 as number of events and number of affected participants in each group. One participant in 150mg and one in 300mg group had slight QTc prolongation>450ms (detailed in Table 9). Participants in the 150mg group reported three UTIs, two cases of pneumonia, one cold virus, one mild back pain, one mild headache, one mild dysgraphia, one mild left foot drag, one mild confusion, one mild hallucination, one mild paranoia, one mild agitation, one moderate anxiety, one mild incontinence, one moderate itching and one skin irritation. Participants in the 300mg group reported one incident of blurry vision, one diarrhea, one nausea, one mild fatigue, and two generalized weakness, three UTIs, one pneumonia, one weight loss, one tooth extraction, one dizziness, two mild hallucination, one mild paranoia, one mild crying episode, one mild urinary urgency, one mild cough and one eczematous lesion.

Table 8- Non-Serious Adverse Events in all participants throughout the study

System Organ Class/Preferred Term	Number of affected participants N=12	Total number of events	Number of affected participants in 150mg group . N=5	Number of affected participants in 300mg group. N=7
Cardiac disorders	2	3	1	1
QTc prolongation			1	2
Eye disorders	1	1	0	1
Mild blurry vision			0	1
Gastrointestinal disorders	2	2	0	2
Diarrhea				1
Nausea				1
General disorders	3	3	0	3
Mild fatigue			0	1
Generalized weakness			0	2
Infections and infestations	8	10	4	4
Urinary tract infection			3	3
Pneumonia			2	1
Cold virus			1	
Metabolism and nutrition disorders	1	1	0	1
Mild weight loss				1
Musculoskeletal and connective tissue disorders	2	2	1	1
Mild back pain			1	
Tooth extraction				1
Nervous system disorders	3	5	2	1
Mild headache			1	
Mild dysphragia			1	
Mild left foot drag			1	
Mild confusion			1	
Dizziness				1
Psychiatric disorders	4	8	3	1
Mild hallucinations			1	2
Mild Paranoia			1	1
Mild crying episodes				1
Mild agitation			1	
Moderate anxiety			1	
Renal and urinary disorders	2	2	1	1
Mild urinary urgency				1
Mild incontinence			1	
Respiratory, thoracic and mediastinal disorders	1	1	0	1
Mild cough				1
Skin and subcutaneous tissue disorders	2	3	1	1
Moderate itching			1	
Moderate skin irritation			1	
[illegible]				[illegible]

9.3.3- QTc prolongation. One participant (NIL-9) in the 300 mg was withdrawn due to moderate cardiac ischemia and myocardial infarct and was double-stented for left bundle block (Table 7&9). NIL-8 had slight QTc prolongation>450ms at week 8, and NIL-15 had two episodes of slight QTc prolongation>450ms at week 2 and week 8 but QTc returned to normal range in subsequent visits. NIL8 withdrew from the study at week 24 due to caregiver burden, but returned for safety follow up visit (week 36) and another patient (NIL-12) did not return at week 36.

Table 9- A detailed list of QTc values throughout the study in the 150mg (top grid) and 300mg (bottom grid) groups. Normal QTc was specified in the study protocol in the range of 350-450ms. EKG: electrocardiogram.

150 mg

EKG QTc (Normal range per study protocol: 350-450 ms)

	Screening	Baseline	Week-2	Week-4	Week-6	Week-8	Week-12	Week-16	Week-20	Week-24	Week-36
NIL-1	421	418	416	421	417	401	428	408	421	419	419
NIL-5	380	411	439	430	445	440	442	435	416	429	429
NIL-8	435	412	419	426	436	456	423	444	441	out	419
NIL-11	399	392	399	411	397	425	417	398	421	410	420
NIL-14	410	424	422	416	424	424	414	424	427	423	426

300 mg

EKG QTc (Normal range per study protocol: 350-450 ms)

	Screening	Baseline	Week-2	Week-4	Week-6	Week-8	Week-12	Week-16	Week-20	Week-24	Week-36
NIL-2	394	409	407	403	387	410	404	414	408	414	414
NIL-3	411	396	405	412	410	409	421	428	407	411	411
NIL-6	410	413	405	434	438	432	444	440	442	432	432
NIL-9	441	430	447	510	out						
NIL-12	431	435	443	440	432	440	444	422	434	424	out
NIL-13	418	415	426	403	409	413	433	412	420	442	388
NIL-15	428	440	456	444	445	463	449	442	446	450	432

9.3.4- Blood chemistry. Laboratory tests (Table 10) were performed throughout eleven visits according to study protocol. Laboratory results of some participants' were outside the normal range at baseline due to comorbidities that were not exclusionary. A total number of three AEs below normal range of ALT level (<100%) were observed in the 150mg group Nilotinib. Two AEs above normal range (>100%), including one ALT and one bilirubin were observed in the 150mg group Nilotinib. Two AEs with an increase of ALT and one AST above normal range (>100%) were observed in the 300mg group Nilotinib.

Table 10- List of results of laboratory tests at baseline and after Nilotinib administration. At baseline, 3 participants had slightly below normal range of ALT, 1 amylase and 1 LDL. A slight increase above the normal range was recorded in WBC in 1 participant, 4 ALT, 1 amylase, 2 cholesterol, 1 HDL, 2 LDL and 3 triglyceride. Low density lipoprotein (LDL): high density lipoprotein (HDL), alanine aminotransferase (ALT), aspartate aminotransferase (AST). white blood cells (WBC).

Normal Range	Lab Test	Baseline (N=12)		Number of events below (<100%) normal range (N-12)		Number of events above (>100%) normal range (N-12)	
		Slightly lower than normal	Slightly higher than normal	Number of events in 150 mg group (N=5)	Number of events in 300 mg group (N=7)	Number of events in 150 mg group (N=5)	Number of events in 300 mg group (N=7)
5-10 k/uL	WBC	0	1	0	0	0	0
12-17 g/dL	Hemoglobin	0	0	0	0	0	0
36-52 %	Hematocrit	0	0	0	0	0	0
145-400 k/uL	Platelet	0	0	0	0	0	0
137-145 mmol/L	Sodium	0	0	0	0	0	0
2.5-4.5 mg/dL	Phosphate	0	0	0	0	0	0
8.4-10.2 mg/dL	Calcium	0	0	0	0	0	0
1.6-2.3 mg/dL	Magnesium	0	0	0	0	0	0
3-34 units/L	AST	0	0	0	0	0	1
15-41 units/L	ALT	3	4	3	0	1	2
0.3-1.9 mg/dL	Total Bilirubin	0	0	0	0	1	0
23-300 units/L	Lipase	0	0	0	0	0	0
30-110 units/L	Amylase	1	1	0	0	0	0
120-200 mg/dL	Total Cholesterol	0	2	0	0	0	0
40-80 mg/dL	HDL	0	1	0	0	0	0
85-125 mg/dL	LDL	1	2	0	0	0	0
50-150 mg/dL	Triglyceride	0	3	0	0	0	0

9.4- Disease related biomarker studies. CSF alpha-Synuclein concentration was significantly reduced at six months compared to baseline in the 150mg group (Table 11, N=4), but alpha-Synuclein did not change in the 300mg group (N=5). CSF HVA also significantly increased in the 300mg group at six-month compared to baseline (Table 11, N=5), but no significant differences were observed in the 150mg group between baseline and six months treatment. CSF total Tau was significantly reduced at six months compared to baseline (Table 11, N=4) in 150mg group and no changes in total Tau were observed in 300mg groups, and these results were confirmed by Invitrogen and Millipore ELISA. CSF pTau181 concentration was increased at six months compared to baseline with 300mg Nilotinib (Table 11, N=5) and the ratio of pTau181:total Tau was increased at six months in 300mg groups (p=0.0225). However, CSF pTau231 was reduced at six months compared to baseline with 150mg (Table, N=4) and 300 mg (Table 11, N=5) Nilotinib and the ratio of pTau23:total Tau did not change.

CSF Aβ40 concentration was reduced at six months compared to baseline with 150mg (Table

Table 11- Concentration of CSF biomarkers at baseline and after 6-months Nilotinib treatment. Data are summarized as Mean±SD with significance at $p<0.05$. A one tailed paired t-test was used for comparisons between baseline and six months treatment with Nilotinib for both 150mg and 300mg treatment groups (* indicates significantly different between baseline and 6 months). Cerebrospinal fluid (CSF), α-synuclein (α-syn), Homovanillic Acid (HVA), β-Amyloid (Aβ), Neuron Specific Enolase (NSE).

CSF						
**Analytes: (pg/ml)**	**0 mths: (N=4)**	**6 mths (150mg Nilo): (N=4)**	**p value :**	**0 mths : (N=5)**	**6 mths (300mg Nilo): (N=5)**	**p value:**
**α-syn**	1831.47±608.08	1633.25±531.72	0.0319*	1263.70±584.80	1464.85±699.64	0.2958
**HVA**	2.15±2.16	3.62±3.07	0.2414	0.8583±0.23	1.69±0.88	0.0012**
**Total tau (Invitrogen)**	302.63±156.29	261±135.49	0.0291*	209.7±91.29	203±51.99	0.3744
**p-tau (181) (Invitrogen)**	22.96±6.34	26±3.12	0.0834	20.87±3.13	25.57±3.30	0.0051**
**p-tau (181)/Total tau (Invitrogen)**	0.076±0.041	0.996±0.024	0.0671	0.0995±0.034	0.126±0.063	0.0225*
**Total tau (millipore)**	1.1475±0.63	0.99±0.60	0.0207*	0.764±.33	0.688±0.14	0.234
**p-tau (231) (millipore)**	2.97±1.74	2.59±1.58	0.0445*	2.30±1.23	2.01±1.00	0.0324*
**p-tau (231)/Total tau (millipore)**	2.59±2.76	2.61±2.63	0.1873	3.01±3.73	2.992±7.14	0.3068
**Aβ42**	860.75±562	726.08±525.51	0.0579	811.96±356.38	808.04±429.68	0.4857
**Aβ40**	2332.6±1228.78	1875.85±1029.95	0.0437*	1817.9±765.70	1951.789±892.16	0.3964
**Aβ42/Aβ40**	0.369±0.457	0.387±0.510	0.1971	0.447±0.465	0.414±0.482	0.2347
**S100B**	1531.84±749.65	968.88±202.18	0.1509	1062.80±291.61	912.21±164.07	0.0294*
**NSE**	96.35±25.48	72.23±36.57	0.0197*	67.20±20.30	74.22±10.82	0.1703

11, N=4), but Aβ40 was stable with 300mg Nilotinib. There was no significant change in CSF Aβ42 (Table 11) and the ratio of CSF Aβ42:Aβ40 was not different. CSF concentration of NSE was reduced in 150mg group (Table 11, N=4) and the glial and neuronal cell death marker, S100B was reduced in 300mg group (Table 11 N=5) at six months compared to baseline.

Plasma concentration of alpha-Synuclein was not significantly different at six months compared to baseline in the 150mg and 300mg group (Table 12). Plasma HVA level trended towards an increase after Nilotinib treatment in 300mg and 150mg groups at six months compared to baseline. The plasma level of Aβ40 did not change after Nilotinib treatment in both 150mg and 300mg groups.

We performed further CSF analysis of oligomeric α-synuclein in the same subjects and

Table 12- Concentrat
Data are summarize
used for comparison
and 300mg treatmen

Analytes	0 mths
α-syn	30463.05±18
HVA	3.73±1.1
Aβ40	38.92±18

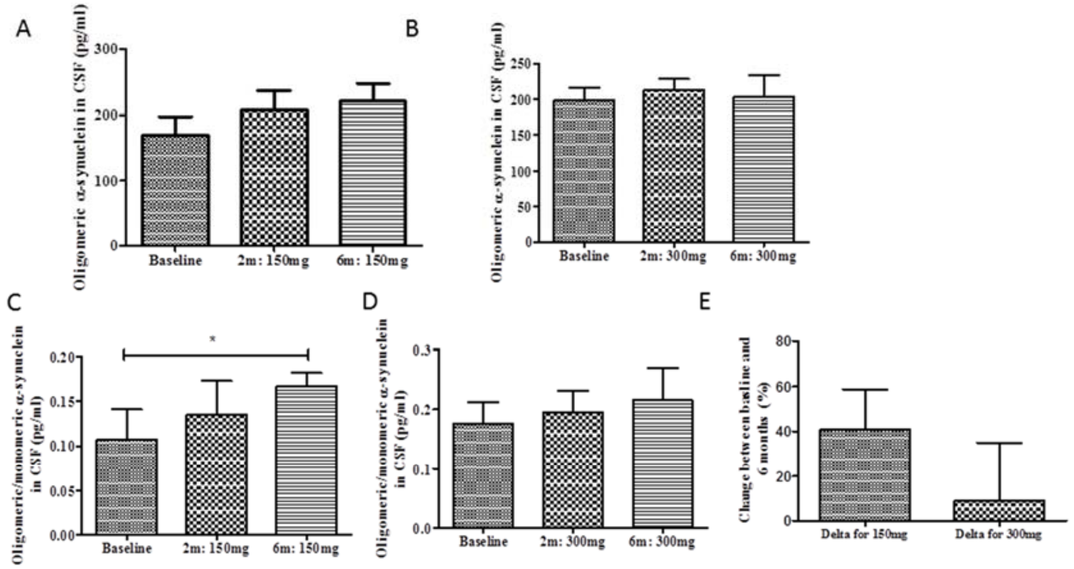

Figure 6- ELISA concentrations of CSF oligomeric alpha-Synuclein in A) 150 mg and B) 300mg Nilotinib groups at baseline, 2 and 6 months treatment. CSF oligomeric alpha-Synuclein normalized to monomeric alpha-Synuclein was significantly increased in C) 150mg group but not the D) 300mg Nilotinib groups between baseline and 6 months treatment. The increase (E) between baseline and 6 months CSF oligomeric alpha-Synuclein is 41% in the 150mg group but this increase was reduced to 8% in the 300mg group. N=5 in 150mg and N=7 in 300mg groups. $p<0.05$.

normalized oligomeric to monomeric α-synuclein. The level of oligomeric α-synuclein was increased in the 150mg group (Fig. 6A) but stable in the 300mg group (Fig. 6B), consistent with the changes in monomeric α-synuclein. However the ratio of oligomeric/monomeric α-synuclein was significantly higher in the 150mg group at 6 months compared to baseline (Fig. 6C, $p<0.05$) but did not change in the 300mg group (Fig. 6D). Additionally, the change in CSF oligomeric/monomeric α-synuclein (Fig. 6E) between baseline and 6 months was 40% in the 150mg group but this increase was attenuated (8%) in the 300mg group, suggesting that Nilotinib reduces CSF oligomeric α-synuclein levels. These results are consistent with our preclinical data (13,26,30) and previous evidence that the ratio of oligomeric/monomeric α-synuclein longitudinally increases in the CSF of PD patients (48,49). These data further suggest that Nilotinib reduces α-synuclein levels in PD but a placebo group is needed to better understand the effects of Nilotinib on α-synuclein species.

9.5- Nilotinib ameliorates motor impairment. Participants' motor symptoms were monitored with UPDRS throughout all visits (Table 13). An average decrease of 3.4 points and 3.6 points in UPDRS-III (motor) was observed at six months compared to baseline with 150mg and 300mg Nilotinib,

Table 13- Detailed UPDRS scores of all participants during every visit. PD: Parkinson's disease; DLB: dementia with Lewy body; MCI: mild cognitive impairment; PDD: Parkinson's disease with dementia; UPDRS: Unified Parkinson's Disease Rating Score.

UPDRS-III 150mg

	Screening	Week-4	Week-8	Week-12	Week-16	Week-20	Week-24	Week-36	Diagnosis	Stage
NIL- 01	38	29	32	32	33	29	23	27	DLB	3
NIL-05	52	55	56	59	59	60	60	64	DLB	5
NIL-08	42	4	39	40	43	42	42	54	DLB	5
NIL-11	33	33	28	30	33	31	29	38	PD-MCI	3
NIL-14	24	26	23	22	22	19	18	26	DLB	3
AVG	37.8	29.4	35.6	36.6	38	36.2	34.4	41.8		

UPDRS-I-IV 150mg

	Screening	Week-4	Week-8	Week-12	Week-16	Week-20	Week-24	Week-36	Diagnosis	Stage
NIL- 01	65	54	57	56	58	53	44	58	LBD	3
NIL-05	100	103	104	107	107	108	108	124	LBD	5
NIL-08	79	40	76	77	80	83	83	95	LBD	5
NIL-11	71	71	63	60	63	57	53	74	PD-MCI	3
NIL-14	48	50	45	47	43	38	40	45	DLB	3
AVG	72.60	63.60	69.00	69.40	70.20	67.80	65.60	79.2		

UPDRS-III 300mg

	Screening	Week-4	Week-8	Week-12	Week-16	Week-20	Week-24	Week-36	Diagnosis	Stage
NIL- 02	16	11	9	9	13	7	9	10	PD-MCI	3
NIL-03	12	14	11	11	16	16	12	21	PD	3
NIL-06	11	7	11	8	6	7	6	16	PDD	3
NIL-12	16	22	18	18	21	21	21	out	PD-MCI	3
NIL-13	77	67	70	67	67	69	70	70	DLB	5
NIL-15	52	47	49	47	44	41	44	42	PDD	5
AVG	30.6	28	28	26.6	27.8	26.8	27	31.8		

UPDRS-I-IV 300mg

	Screening	Week-4	Week-8	Week-12	Week-16	Week-20	Week-24	Week-36	Diagnosis	Stage
NIL- 02	36	31	28	26	33	23	20	30	PD-MCI	3
NIL-03	33	45	41	41	46	41	28	54	PD	3
NIL-06	28	21	25	19	17	16	15	30	PDD	3
NIL-12	43	49	45	46	49	54	45	out	PD-MCI	3
NIL-13	126	116	118	115	113	106	107	109	DLB	5
NIL-15	109	103	105	97	95	92	95	92	PDD	5
AVG	62.5	60.8	60.3	57.3	58.8	55.3	51.6	63		

Table 14- Summary of statistical analyses of Unified Parkinson's Disease Scores (UPDRS), Mini Mental Status Exam (MMSE) and Scales for Outcomes in Parkinson's Disease-Cognition (SCOPA-Cog) at 6 months compared to baseline in 150mg and 300mg Nilotinib. Data are mean±SD. (*) indicates significantly different to baseline

	150 mg, N=5				300 mg, N=7		
	Baseline	Week-24	p-value		Baseline	Week-24	p-value
UPDRS-III	37.8 ± 10.4	34.4 ± 16.9	0.2087		30.7 ± 27.4	27 ± 25.2	0.07
UPDRS I-IV	72.6 ± 19.1	65.6 ± 29.1	0.1456		62.5 ± 43.2	51.7 ± 39.7	0.0098 **
MMSE	15.4 ± 11	19.3 ± 11.8	0.1332		17 ± 13.1	20.5 ± 10.7	0.013 *
SCOPA-Cog (**/43)	9.4 ± 10.4	11.3 ± 11	0.3188		10.3 ± 10.7	12.3 ± 12.1	0.1082

respectively. An average decrease of 7 points and 11.1 points in UPRDS I-IV was observed at six

months in 150mg and 300mg groups, respectively. An average decrease of 7 points and 11.1 points in UPRDS I-IV were observed at 6 months in 150mg and 300mg groups, respectively. In the 300mg Nilotinib group, UPDRSI-IV significantly improved (p=0.0098) at six months compared to baseline (Table 14). The improvement in UPDRS was progressive throughout the six months (24 weeks) trial, and this improvement was reversed in participants (who returned) in the follow up visit off drug at thirty six weeks.

9.6- Nilotinib improves cognitive symptoms. Participants' progression of cognitive decline was monitored with MMSE throughout all visits (Table 15). An average increase of 3.85 (out of 30) and 3.5 points in MMSE was observed at six months compared to baseline with 150mg and 300mg Nilotinib, respectively. In the 300mg group MMSE significantly increased (p=0.013) at six months compared to

Table 15- Detailed MMSE and SCOPA-Cog scores during every visit. PD: Parkinson's disease; DLB: dementia with Lewy body; MCI: mild cognitive impairment; PDD: Parkinson's disease with dementia; MMSE: Mini Mental State Examination. SCOPA-Cog: Scales for Outcomes in Parkinson's Disease-Cognition

MMSE
150 mg

Subjects	Screening	Week-4	Week-8	Week-12	Week-16	Week-20	Week-24	Week-36	Diagnosis	Stage
NIL-01	17	20	22	21	26	26	23	15	DLB	3
NIL-05	0	0	1	1	1	1	2	0	DLB	5
NIL-08	9	11	11	11	8	10	out	4	DLB	5
NIL-11	26	28	29	29	30	30	29	29	PD-MCI	3
NIL-14	25	28	27	28	28	25	23	30	DLB	3
AVG	15.4	17.4	18	18	18.6	18.4	19.25	15.6		

MMSE
300 mg

	Screening	Week-4	Week-8	Week-12	Week-16	Week-20	Week-24	Week-36	Diagnosis	Stage
NIL-02	26	27	28	29	27	26	28	25	PD-MCI	3
NIL-03	30	28	23	26	29	30	30	30	PD	3
NIL-06	22	24	24	25	25	25	25	15	PDD	3
NIL-12	23	28	24	28	14	24	26	out	PD-MCI	3
NIL-13	0	5	2	1	6	2	5	0	DLB	5
NIL-15	1	10	8	6	12	6	9	7	PDD	
AVG	17	20.3	18.1	19.1	18.8	18.8	20.5	15.4		

SCOPA-Cog (**/43)
150 mg

	Screening	Week-8	Week-20	Week-24	Diagnosis	Stage
NIL-01	5	5	3	4	LBD	3
NIL-05	0	0	1	0	LBD	5
NIL-08	1	4	0	out	LBD	5
NIL-11	22	23	26	23	PD-MCI	3
NIL-14	19	18	18	18	LBD	3
AVG	9.4	10	9.6	11.25		

SCOPA-Cog (**/43)
300 mg

	Screening	Week-8	Week-20	Week-24	Diagnosis	Stage
NIL-02	15	22	21	22	PD-MCI	3
NIL-03	28	30	30	31	PD	3
NIL-06	13	11	19	10	PDD	3
NIL-12	6	9	10	10	PD-MCI	3
NIL-13	0	0	0	0	DLB	5
NIL-15	0	1	0	1	PDD	
AVG	10.33	12.17	13.3	12.33		

baseline (Table 14). The increase in MMSE scores was incremental throughout the twenty four-week treatment, and it declined in the thirty weeks-week follow up visit. Improvement in cognitive symptoms was also observed using Scales for Outcomes in Parkinson's Disease-Cognition (SCOPA-Cog) with

an average increase of 1.85 (out of 43) and 2 points at six months compared to baseline with 150mg and 300mg Nilotinib, respectively.

10- TREATMENT ADMINSTERED

10.1- Name of the drug and all active ingredients

One hard white to yellowish powder in red opaque hard gelatin (Tasigna) capsule contains 150 mg Nilotinib (as hydrochloride monohydrate) and 117.08 mg lactose (as monohydrate). The Investigator Brochure (IB) indicating the pharmacological, safety and kinetics of the drug is attached.
In brief, Nilotinib has been clinically prescribed since FDA approval in 2007, and substantial pharmacokinetics data are published (27-29,50). TKIs such as Imatinib (Glevec) are effective in many patients with CML in chronic phase (51,52). Nilotinib (AMN107) is a second generation selective Bcr-Abl inhibitor, which is effective following Imatinib resistance and intolerance (51). Nilotinib was approved by the FDA in 2007 for CML treatment (up to 1200 mg orally daily) (27-29).

10.2- Pharmacological Class of the Drug

Nilotinib belongs to the pharmacotherapeutic group: Antineoplastic agents, protein kinase inhibitors, ATC code: L01XE08
Nilotinib is a potent inhibitor of the Abl tyrosine kinase activity of the BCR-ABL oncoprotein both in cell lines and in primary Philadelphia-chromosome positive leukemia cells. The substance binds with high affinity to the ATP-binding site in such a manner that it is a potent inhibitor of wild-type BCR-ABL and maintains activity against 32/33 imatinib-resistant mutant forms of BCR-ABL. As a consequence of this biochemical activity, Nilotinib selectively inhibits the proliferation and induces apoptosis in cell lines and in primary Philadelphia-chromosome positive leukemia cells from CML patients. In murine models of CML, as a single agent Nilotinib reduces tumor burden and prolongs survival following oral administration.

Nilotinib has little or no effect against the majority of other protein kinases examined, including Src, except for the platelet derived growth factor (PDGF), Kinase tyrosine kinase (KIT) and Ephrin receptor kinases, which it inhibits at concentrations within the range achieved following oral administration at therapeutic doses recommended for the treatment of CML.

10.3- Structural Formula of the Drug

Nilotinib has the molecular formula $C_{28}H_{22}F_3N_7O$ and a molecular weight of 529.52g/mol. The structural formula of Nilotinib is as follows:

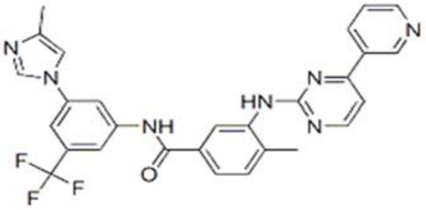

10.4- Formulation of the dosage form to be used

One hard white to yellowish powder in red opaque hard gelatin (Tasigna) capsule contains 150 mg Nilotinib (as hydrochloride monohydrate) and 117.08 mg lactose (as monohydrate). No excipients have any known effects and the full list of excipients in hard capsule includes:
Lactose monohydrate
Crospovidone
Poloxamer 188
Silica, colloidal anhydrous
Magnesium stearate
Hard capsule shell
Gelatin

Titanium dioxide
Red iron oxide
Yellow iron oxide
Printing ink
Shellac
Black iron oxide.

Placebo: Matching placebo that contains all ingredients except Nilotinib will be used. The drug and matching placebo will be provided by Novartis.

10.5- Summary of Previous Human Experience

Several oncology clinical trials data were reported for Tasigna. Exposure to Tasigna in a total of 717 patients from a randomized Phase III study in patients with newly diagnosed Ph+ CML in chronic phase treated at the recommended dose of 300 mg twice daily (n=279) and from an open-label multicenter Phase II study in patients with imatinib-resistant or intolerant CML in chronic phase (n=321) and accelerated phase (n=137) treated at the recommended dose of 400 mg twice daily. No data were reported on CNS clinical trials. The following safety data were reported:

a- ***In patients with newly diagnosed CML in chronic phase***

The median duration of exposure was 48.0 months (range 0.1-58.7 months). The most frequent (≥10%) non-hematological adverse reactions were rash, pruritus, headache, nausea, fatigue, alopecia and myalgia. Most of these adverse reactions were mild to moderate in severity. Upper abdominal pain, constipation, diarrhea, asthenia, dry skin, muscle spasms, arthralgia, vomiting, abdominal pain and peripheral edema were observed less commonly (<10% and ≥5%) were of mild to moderate severity, manageable and generally did not require dose reduction.

Discontinuation due to adverse drug reactions was observed in 9% of patients.

Treatment-emergent hematological toxicities include myelosuppression: thrombocytopenia (18%), neutropenia (15%) and anemia (7%). Pleural and pericardial effusions, regardless of causality, occurred in 1% and <1% of patients, respectively, receiving Tasigna 300mg twice daily. Gastrointestinal hemorrhage, regardless of causality, was reported in 3% of these patients.

The change from baseline in mean time-averaged QTcF interval at steady state was 6 msec. No patient had an absolute QTcF >500 msec while on the study medicinal product. QTcF increase from baseline exceeding 60 msec was observed in <1% of patients while on the study medicinal product. No sudden deaths or episodes of torsade de pointes (transient or sustained) were observed. No decrease from baseline in mean left ventricular ejection fraction (LVEF) was observed at any time during treatment. No patient had a LVEF of <45% during treatment nor an absolute reduction in LVEF of more than 15%.

b- ***In patients with imatinib-resistant or intolerant CML in chronic phase and accelerated phase***

The data described below reflect exposure to Tasigna in 458 patients in an open-label multicenter Phase II study in patients with imatinib-resistant or intolerant CML in chronic phase (n=321) and accelerated phase (n=137) treated at the recommended dose of 400 mg twice daily. The most frequent (≥10%) non hematological drug-related adverse events were rash, pruritus, nausea, fatigue, headache, vomiting, myalgia, constipation and diarrhea. Most of these adverse events were mild to moderate in severity. Alopecia, muscle spasms, decreased appetite, arthralgia, abdominal pain, bone pain, peripheral edema, asthenia, upper abdominal pain, dry skin, erythema and pain in extremity were observed less commonly (<10% and ≥5%) and have been of mild to moderate severity (Grade 1 or 2). Discontinuation due to adverse drug reactions was observed in 16% of chronic phase and 10% of accelerated phase patients.

Treatment-emergent hematological toxicities include myelosuppression: thrombocytopenia (31%), neutropenia (17%) and anemia (14%). Pleural and pericardial effusions as well as complications of fluid retention occurred in <1% of patients receiving Tasigna. Cardiac failure was observed in <1% of patients. Gastrointestinal and CNS hemorrhage were reported in 1% and <1% of patients, respectively. QTcF exceeding 500 msec was observed in <1% of patients. No episodes of torsade de pointes (transient or sustained) were observed.

Most frequently reported adverse reactions in Tasigna clinical studies for cancer:

Non-hematological adverse reactions (excluding laboratory abnormalities) are reported in at least 5% of the patients in Tasigna clinical studies. These are ranked under heading of frequency using one decimal precision for percentages and the following convention: very common (≥1/10) or common (≥1/100 to <1/10). Within each frequency grouping, adverse reactions are presented in order of decreasing seriousness.

10.6- Status of Drug in Other Countries

Tasigna is available in the United States, Europe and other countries of the world and requires a prescription. Nilotinib is subject to restricted medical use and the marketing authorization holder shall submit periodic safety update reports for this product in accordance with the requirements set out in the list of Union reference dates (EURD list) provided for under Article 107c(7) of Directive 2001/83/EC and published on the European medicines web-portal determine compliance.

10.7- Drug Related Risks and Potential Side Effects

Nilotinib has a number of side effects as stated in the IB, including gastrointestinal complications, vomiting and nausea, and in some rare cases dizziness (27). Abl has an essential role in mammalian heart growth and development and adult complications with Abl inhibition, include edema, nausea/vomiting, muscle cramps, neutropenia, thrombocytopenia, fever, liver toxicity, arthralgia, and exanthema/rash (40). Side effects are identified for Abl in homozygous Abl mutant mice, which display dramatically enlarged hearts due to abnormally increased cardiomyocyte proliferation during later stages of embryogenesis (41). Disruption of *Abl* in mice results in neonatal lethality accompanied by pleiotropic developmental defects with variable penetrance, including ranting, splenic and thymus atrophy, B cell lymphopenia, dysfunctional osteoblasts, and foreshortened crania (42-44). However, clinical use of Nilotinib (600-800mg daily) is fairly tolerated in CML patients, but no clinical and longitudinal data exist about the long term side effects of this drug since its approval in 2007. Therefore, clinical use of Abl inhibition may have dose-limiting toxicity but we will use much lower doses in the current studies (see study design). Importantly, Nilotinib is washed out of the brain within several hours (13,23), so lower dose and prolonged period of administration may decrease misfolded protein levels, leading to slowing of motor, non-motor and cognitive decline. Furthermore, to evaluate adverse drug effects, all patients will be regularly (every two week for the first two months) monitored for specific warnings, including prolongation of QT interval. Prior to administration and periodically, patients will be monitored for hypokalemia or hypomagnesaemia. EKGs will also be obtained to monitor the QTc at baseline, 7 days after initiation, and periodically thereafter as detailed in Table 2. Nilotinib will not be administered to patients with hypokalemia, hypomagnesaemia, or long QT syndrome. Concomitant drugs known to prolong the QT interval and strong CYP3A4 inhibitors will also be avoided.

Nilotinib should be taken on an empty stomach. It should be taken one hour before eating, or 2hrs after eating to ensure appropriate absorption.

11-PRIOR AND CONCOMITANT THERAPY

Throughout the study, the subject may be prescribed concomitant medications or treatments deemed necessary to provide adequate supportive care, provided that the medications are licensed in the United States. All concomitant medications and/or treatments received by a subject should be recorded on the appropriate source document and eCRF.

Prohibited and contra-indicated medications in those receiving Nilotinib are listed in the inclusion/exclusion criteria and detailed below.

11.1- Prohibited Medications and Contraindications

Prohibited Medications

Prohibited medications for study subjects are as follows:

- Anti-coagulant medications, including Coumadin, heparin, enoxaparin, fondiparinaux, etc
- CYP3A4 enzyme inhibitors, including grapefruit juice
- St John's Wort

Pregnancy & Nursing Mothers

There are no adequate and well-controlled studies in pregnant women. Subjects or partners of male subjects must not become pregnant during the study or 3 months after stopping study drug. If a female subject becomes pregnant, study treatment must be discontinued immediately. Caution should be exercised; therefore, no subject should nurse their infant while participating in this study.

12-RATIONALE TO USE NILOTINIB TO TREAT NEURODEGENERATIVE DISEASES. Neurodegenerative diseases, including PD, are a group of genetic and sporadic disorders associated with neuronal death and progressive nervous system dysfunction. Cancer is also a collection of related genetic diseases, in which cells begin to divide without stopping and spread into surrounding tissues. Unlike neurodegeneration, in which no regeneration happens when damaged or aging post-mitotic neurons die, damaged cells survive when they should die in cancer, resulting in uncontrolled mitotic cell division to form tumors. Cancerous tumors are malignant as they spread or invade nearby tissues by cellular contiguity or metastasize via blood and/or humoral transport. In neurodegeneration, the spread of disease by contiguity is supported by the hypotheses that toxic or "prion-like" proteins propagate along neuroanatomical pathways (53), leading to progressive spread of disease and cell death. In neurodegeneration, failure of cellular quality control mechanisms leads to inadequate protein degradation via the proteasome or autophagy (54), resulting in intracellular accumulation of neurotoxic proteins. Consequently, these proteins are secreted from a pre-synaptic neuron and can traverse the synaptic cleft and enter a contiguous post-synaptic neuron. Secreted proteins may not penetrate an adjacent cell via the synapse but they may be re-routed into the cell and recycled via the endosomal system to fuse with autophagic vacuoles like the autophagosome or the lysosome (55-57). Microglia, the brain resident immune cells may also phagocytose and destroy toxic proteins (58).

Accumulation of neurotoxic proteins, including alpha-Synuclein (Lewy bodies), beta-amyloid plaques, Tau tangles, Huntington, prions and TDP-43 are major culprits in neurodegeneration. These toxic proteins trigger progressive apoptotic cell death leading to loss of many central nervous system (CNS) functions, including mentation, cognition, language, movement, gastrointestinal motility, sleep and many others. The discoveries of toxic protein propagation from cell to cell (53), leading to progression of neurodegeneration triggered a series of pre-clinical and clinical studies to limit protein propagation via antibodies (active and passive immune therapies) that can capture the protein and destroy it en route to healthy neurons. This approach is fraught with difficulties, including failure to arrest neurocognitive decline and brain edema/inflammation. Manipulation of autophagy is a novel therapeutic approach that focuses on degradation of neurotoxic proteins at the manufacturing site in order to prevent their secretion and propagation. This novel strategy leads to unclogging the cell's disposal machine and degradation of toxic proteins, thus preserving neuronal survival via bulk digestion. Preservation of neuronal survival maintains the level of neurotransmitters that are necessary for cognitive, motor and other CNS functions, leading to alleviation of symptoms as well as arrest of neurodegeneration. As neurons are post-mitotic cells, pulsatile autophagy may promote protein degradation and provide an effective disease-modifying therapy for neurodegenerative diseases.

Autophagy is a double-edged sword in cancer, either preventing accumulation of damaged proteins and organelles to suppress tumors, or promoting cell survival mechanisms that lead to tumor growth and proliferation (59). Leukemia and many other cancer treatments have been revolutionized by manipulation of autophagy, which leads to bulk degradation of unwanted or toxic molecules (60). For example in leukemia, genetic mutations and DNA damage can lead to large numbers of abnormal white blood cells (leukemia cells and leukemic blast cells) to accumulate in the blood and bone marrow, crowding out normal blood cells. Autophagy can lead to the degradation of the products of cancer-causing genes (oncogenes), tumor suppressor genes, damaged DNA and essential components of the cytosol, thereby controlling abnormal mitotic division and limiting tumor growth. Autophagy can also lead to self-cannibalization via promotion of programmed cell death, or apoptosis (61,62). Activation of the tumor suppressor p53 in response to DNA damage leads the cell to arrest proliferation, initiate DNA repair, and promote survival. However, if the DNA damage cannot be resolved by p53, it can trigger

apoptotic death (61,62). Cell division and apoptosis are mediated by signaling mechanisms via the endosomal (early and recycling) system (55). Tyrosine kinases are activated via auto phosphorylation, triggering various signaling mechanisms that mediate cell division and/or apoptosis (63,64). Tyrosine kinase inhibition via de-phosphorylation leads to signaling via the late endosomal-lysosomal pathway, thus increasing autophagic degradation (55-57) and tumor growth.

TKIs have significantly improved the life quality and expectancies in many cancers, including CML (65,66). CML is characterized by the translocation of chromosomes 9 and 22 to form the "Philadelphia" chromosome resulting in the expression of a constitutively active Breakpoint Cluster Region-Abelson (BCR-Abl) tyrosine kinase. This oncogenic protein activates intracellular signaling pathways and induces cell proliferation. Our laboratory investigated TKIs that activate autophagy and are FDA-approved for CML, thus significantly reducing research and development efforts and cost by re-purposing (reviewed in (33)) for new indications. Abl is activated in neurodegeneration (24,32,61,62). A fraction of Nilotinib crosses the blood-brain-barrier (BBB), inhibits Abl and facilitates autophagic amyloid clearance, leading to neuroprotection and improved cognition and motor behavior (25,26,30-34). Mice treated with a much lower dose of these drugs (<25% of the typical CML dose) show significant motor and cognitive improvement and degradation of alpha-Synuclein, beta-amyloid, Tau and TDP-43 without evidence of increased inflammation (25,26,30-34). There was also significant reversal of neurotransmitter alterations, including dopamine and glutamate in several models of neurodegeneration. As a modulator of myeloid cells (67), Nilotinib may also positively regulate neuronal death and produce neuro-restorative effects via increased production of necessary growth factors and proliferation of myeloid-derived glia. Autophagic toxic protein clearance and production of growth factors may restore loss of neurotransmitters, leading to improved motor and cognitive functions. Nilotinib provides a double-edge sword via manipulation of autophagy to inhibit cell division and tumor growth in CML on one hand, and promote toxic protein degradation and neuronal survival in neurodegeneration on the other hand.

13-CLINICAL ASSESSMENTS AND OUTCOME MEASURES

13-1. Clinical Variables

Assessments will be performed at visits as noted above throughout the study for clinical evaluation. In addition to the assessments evaluated below, subjects will provide information on their demographics, past medical history, including PD, as well as concomitant medication usage.

13.2- Vital Signs, Height & Weight

Vital signs, including systolic and diastolic blood pressure, pulse rate (radial artery)/minute, respiratory rate/minute, temperature and weight will be assessed at specified visits. Height will be measured and recorded at the Screening Visit only.

Medical history will be assessed as follows:

- History of PD (inquiring on date of diagnosis and first symptoms)
- PD treatment history
- Smoking history
- Important medical information for inclusion/exclusion
- Significant medical and surgical history (e.g. Allergy/Immunologic, Cardiovascular, Dermatological, ENT, Gastrointestinal, Gynecologic/Urologic, Hepatobiliary, Hermato/Lymphatic, Metabolic/Endocrine, Musculoskeletal, Neurologic, Ophthalmologic, Psychiatric, Pulmonary, renal, and Other)

Questioning of Comorbidities will be done using the Charlston Comorbidity Index and should be done at every visit after Baseline:

Metastatic solid tumor:	no/yes
AIDS:	no/yes
Moderate-to-severe liver disease:	no/yes
Hemiplegia:	no/yes
Moderate-to-severe renal failure:	no/yes
Moderate-Diabetes with endorgan damage:	no/yes
Neoplasia:	no/yes
Leukemia:	no/yes
Lymphoma:	no/yes
Myocardial infarct:	no/yes
Congestive heart failure:	no/yes
Peripheral vascular disease:	no/yes
Cerebrovascular disease:	no/yes
Dementia:	no/yes
Chronic pulmonary disease:	no/yes
Connective tissue disease:	no/yes
Ulcer disease:	no/yes
Mild liver disease:	no/yes
Diabetes:	no/yes

- Concomitant medications will be recorded at every visit after baseline.

13.3- Clinical Laboratory Assessments

The following safety laboratory tests will be performed during the study:

Standard blood chemistry, including Cholesterol, low density lipoprotein (LDL), high density lipoprotein (HDL), albumin, total protein, alkaline phosphatase, total bilirubin, creatinine, calcium, chloride, sodium, potassium, magnesium, inorganic phosphorus, bicarbonate, creatine phosphokinase (CPK), gamma-glutamyl transferase (γ-GT), lactate dehydrogenase (LDH), lipase, α-amylase, alanine aminotransferase (ALT), aspartate aminotransferase (AST), blood urea nitrogen (BUN), thyroid stimulating hormone (TSH), vitamin B12, glycated urea, uric acid. A standard hematology panel including, complete blood count (CBC) with differential counts will be performed. Hemoglobin (Hb), hematocrit, red blood cell count, platelet count, and white blood cell count with differential count will be measured. In addition, prothrombin time-international normalized ratio (PT-INR) will be measured for assessment of coagulation.

- Serum hCG for women of childbearing potential (WOCBP)

Additional testing may be ordered if needed, to further assess an AE, or if there is any suspicion that a subject may be pregnant, throughout the course of the study.

13.4- Physical Examination
A physical examination will be performed and recorded. A full physical examination (including vital signs) will be performed by a trained physician as indicated in the schedule of assessments (Table 2). The Physical examination will include:
- General appearance
- Abdomen
- Cardiovascular systems
- Lungs
- Lymph nodes
- Musculoskeletal systems
- Skin (with special attention to dermatological tolerability)
- Extremities
- Head, ears, eyes, nose, throat, and mouth
- Thyroid gland
- Vital signs:

    Body weight (Kg or pound)
    Height (cm or inch) (at the Screening Visit only)
    Blood pressure systolic and diastolic (mmHg)
    Heart rate (bpm)

13.5- Neurological Examination
A neurological examination will be performed and recorded as detailed in Table 2. Neurological examination will include:
- Cranial nerves
- Motor functions
- Sensation
- Coordination
- Speech

13.6- Adverse Events

AEs will be documented at each study visit, including the Screening Visit once the ICF has been signed by the subject. Information on AEs of study medication and on inter-current events will be determined at each visit by direct questioning of the subjects review of concomitant medications, and vital sign results. AEs are incidents or complaints that do not require hospitalization, SAEs require hospitalization (see section 13).

13.7- MDS-UPDRS (includes Hoehn & Yahn staging) is used to follow the longitudinal course of PD and it is the most commonly used scale in the clinical study of PD. UPDRS motor section in particular is used to follow the progression of a person's PD. The UPDRS is made up of these sections: Part I: evaluation of mentation, behavior, and mood. Part II: self-evaluation of the activities of daily life (ADLs) including speech, swallowing, handwriting, dressing, hygiene, falling, salivating, turning in bed, walking, and cutting food. Part III: clinician-scored monitored motor evaluation. Part IV: complications of therapy. Part V: *Hoehn and Yahr* staging of severity of Parkinson's disease. Part VI: Schwab and England ADL scale

13.8- PDQ-39. The 39-item Parkinson's Disease Questionnaire (PDQ) is widely used in patient-reported clinical trial endpoint. The PDQ-39 is a 39-item self-report questionnaire, which assesses PD-specific health related quality. It assesses how often patients experience difficulties across the 8 quality of life (QoL) dimensions. Statistical criteria 39-multiple-choice items covering 8 dimensions (number of items in each dimension), including, Mobility (10, #1-10), Activities of daily living (ADL) (6, #11-16), Emotional well-being (6, #17-22), Stigma (4, #23-26) • Social support (3, #27-29), Cognition (4, #30-33), Communication (3, #34-36) and Bodily discomfort (3, #37-39). Five-point ordinal scoring system: 0 = never, 1 = occasionally, 2 = sometimes, 3 = often, 4 = always. Each dimension total score range from 0 (never have difficulty) to 100 (always have difficulty). Lower scores reflect better QoL. Dimension score = sum of scores of each item in the dimension divided by the maximum possible score of all the items in the dimension, multiplied by 100. Overall score can be summarized in the PD Summary Index (PDSI) or PDQ-39 Summary Index (PDQ-39 SI). PDSI or PDQ-39 SI = sum of dimension total scores divided by 8.

13.9- MOCA is designed as a rapid screening instrument for mild cognitive dysfunction. It assesses different cognitive domains, including attention and concentration, executive functions, memory, language, visuo-constructional skills, conceptual thinking, calculations and orientation. It has excellent psychometric properties and has become a widely used screening instrument for mild cognitive impairment (Smith, Gildeh & Holmes, 2007). It is administered by a nurse or clinician and takes 10 - 20 minutes.

13.10- C-SSRS

The US FDA recommends the use of a suicidality assessment instrument that maps to the Columbia Classification Algorithm for Suicide Assessment (C-CASA). The C-CASA was developed to assist the FDA in coding suicidality data accumulated during the conduct of clinical trials of antidepressant drugs. One such assessment instrument is the Columbia Suicide Severity Rating Scale (C-SSRS). The C-SSRS involves a series of probing questions to inquire about possible suicidal thinking and behavior. The US FDA also recommends assessment of depression during every visit, so the Geriatric Depression Scale (GDS) will also be performed.

Only investigators who have been fully trained in the administration of the C-SSRS and GDS will assess subject suicidality and depression. As part of training, investigators are prepared to respond to and manage instances in which patients express suicidal ideation or exhibit suicidal behavior.

At the Baseline Visit, the C-SSRS *Baseline* version will be administered. This version is used to assess suicidality over the subject's lifetime and specifically for the previous 6-month time period.
At each visit and the Final Safety visit, as applicable, the *Since Last Visit* version of the C-SSRS will be administered. This version of the scale assesses suicidality since the subject's last visit.
Information obtained from: *http://www.cssrs.columbia.edu/*

13.11- GDS. The FDA recommends that clinical trial participants should be tested for depression during each visit. The GDS tests geriatric depression, which is a mental and emotional disorder affecting senior citizens. Lasting depression is not only a typical part of aging but may also be a result of neurodegenerative diseases or use of medication. Subsyndromal depression doesn't meet the full criteria for major depression. The National Institute for Mental Health estimates that as many as five million elderly people in the U.S. suffer from subsyndromal depression, which can lead to major depression if left untreated and may increase the risk for suicide. The scale is a 15 item test that will examine participants' depression.

13.12- GSRS evaluates gastrointestinal symptoms, including diarrhea and other possible AEs associated with Nilotinib administration. GSRS is an interview-based rating scale consisting of 15 items for assessment of gastrointestinal symptoms, including dispepsia, ulcer, diarrhea, irritable bowel syndrome, constipation, nausea, etc.,, that may been developed during the course of Nilotinib trials. The scale is easy to apply and proved to be useful in comparing the effectiveness of different modes of treatment in clinical trials.

13.13- CSF and Blood Biomarkers
Subjects will also be asked to provide blood samples for biomarker analysis per Schedule of Activities. Blood samples will be stored in a sample repository at Georgetown University Medical Center (GUMC) laboratory for Dementia and Parkinsonism, where bio fluids will be indefinitely stored and may be used for further biomarker validation studies. All samples will be labeled with a code. The code will not include any identifiable information. Any analysis performed on these samples is for research purposes only. Unused samples will remain in the biorepository for future PD-related research. There is no scheduled date on which the samples will be destroyed. Samples may be stored for research until they are used, damaged, decayed or otherwise unfit for analysis. Subjects have the option of declining participation in this portion of the study at any time by withdrawing their consent to have their sample used. However, it will not be possible to destroy samples that may have already been used.

14- BIOMARKERS

14-1- Rationale for CSF and plasma biomarkers. The biomarkers proposed include CSF levels of alpha-Synuclein, total Tau and p-$Tau_{181/231}$, which yielded useful results in our open label pilot trial and are identified as potential CSF biomarkers for PD pathology.
Several studies indicate that reduction of CSF alpha-Synuclein is associated with alpha-Synuclein pathology in the CNS (68-70). CSF alpha-Synuclein, which probably comes from a neuronal source, has a lower level than the highly abundant presence of peripheral alpha-Synuclein (71). However, only a few studies have investigated CSF alpha-Synuclein levels in patients with neuropathologically confirmed alpha-Synucleinopathies (37,72). CSF alpha-Synuclein is reduced in de novo PD patients compared with healthy individuals (73), and it is lower in patients with advanced alpha-Synucleinopathies compared to other neurological disorders (72). Our pilot study showed that alpha-Synuclein is significantly decreased with 150mg Nilotinib between baseline and 6 months, perhaps

reflecting the natural progression of the disease. However, 300mg Nilotinib attenuates the loss of CSF alpha-Synuclein between baseline and 6 months treatment. Therefore, we expect Nilotinib to reduce the decrease in CSF alpha-Synuclein level indicating reduced cell death, which will also be compared with direct markers of cell death, including NSE and S100B.

Tau pathology is also frequently found in the CNS of PD patients and have been associated with the development of cognitive impairment and PD dementia (74). Several studies also demonstrate increased levels of CSF total Tau and p-Tau181 levels (37-39) in PD patients, but another study report slightly decreased or normal levels of CSF total Tau and p-Tau (75). The CSF variably as reported in the literature in CSF Tau levels may be due to disease stage and/or differences in diagnostic criteria. Therefore, based on the preliminary data we obtained from our pilot trial showing changes in CSF Tau with Nilotinib treatment, we will measure CSF Tau in this study as another marker of cell death and compare Tau with NSE and S100B at baseline and 6 months in a placebo-controlled study.

Other candidate biomarkers for PD include catecholamines, such as dopamine and noradrenaline, and their metabolites. Levels of CSF HVA, which is the end byproduct of the neuronal metabolite of dopamine is reduced in PD (76,77), reflecting diminution of stores of central dopamine. We will measure CSF HVA and quantify DaTscan to measure dopamine transporters activity in collaboration with GE Healthcare at baseline, 3 months (interim) and 6-months to correlate imaging and CSF biomarkers of dopamine metabolism. DaTscan is clinically useful in early stages of PD and since we observe an increase in HVA level with Nilotinib treatment, we expect Nilotinib to increase HVA levels, reflecting an increase in central dopamine stores. We also expect Nilotinib to increase dopamine transporters activity, which will be quantified at baseline and compared to 3 and 6 months Nilotinib treatment. We will also measure changes in exploratory biomarkers of pathophysiology, which may show Nilotinib effects on biomarker levels that will help us to build a better clinical development program going forward.

CSF, plasma PD biomarkers: Blood draw (15ml) and lumbar puncture (LPs) to obtain CSF (~15ml) will be performed on all patients at baseline and 12-months after treatment. Blood will be drawn 0.5hr before LP s outlined above. Plasma will be isolated immediately after blood draw and will be aliquoted and stored at -80ºC. CSF will be aliquoted and stored at -80ºC. Freeze and thaw cycles will be avoided. To avoid CSF contamination with blood, the first 1 mL of CSF collection will be discarded and all samples will be centrifuged at 1000g for 15 minutes. Samples that contain a detectable level of Hemoglobin will be eliminated from alpha-Synuclein and HVA evaluation.

Plasma and CSF sample preparation for Mass Spec to determine Nilotinib pharmacokinetics

Plasma and CSF samples (20 µl) will be thawed initially on ice at room temperature and transfused to Eppendorf tubes containing 100µl of water. 500µl extraction solvent, Acetonitrile/Methanol (50:50) containing the internal standard (5ng/mL of Nilotinib_13C_2H3) will be added to the sample. The mixture will be vortexed and incubated for 20min on ice to accelerate protein precipitation. After incubation, the samples will be vortexed and centrifuged at 13,000 rpm for 20 min at 4ºC. The supernatant is freeze- dried using speed vacuum and reconstituted in 200µL of Methanol: Water (50:50) and processed by Mass Spectrometry.

14.2- Pharmacokinetics. Plasma and CSF will be collected 2hrs (T_{max} Figure 5) after oral administration of Nilotinib to determine the pharmacokinetic parameters of Nilotinib after dosing with 150 mg and 300 mg. Note that no Nilotinib was detected in CSF after 4 hrs. in our prior study (Figure 5 A). Quantitation of Nilotinib will be performed using multiple reactions monitoring mass spectrometry. The samples will be resolved on an Acquity UPLC BEH C18 1.7µm, 2.1 x 50 mm column online with a triple quadrupole mass spectrometer (Xevo-TQ-S, Waters Corporation, USA) operating in the multiple

reaction monitoring (MRM) mode (The sample cone voltage and collision energies will optimized for both analytes to obtain maximum ion intensity for parent and daughter ions using "IntelliStart" feature of MassLynx software (Waters Corporation, USA). The instrument parameters will be optimized to gain maximum specificity and sensitivity of ionization for the parent [m/z = 530.27 (Nilotinib), 438.25 and daughter ions [m/z = 289.01 (Nilotinib). Signal intensities from all MRM Q1/Q3 ion pairs for both analytes are ranked to ensure selection of the most intense precursor and fragment ion pair for MRM-based quantitation. The metabolite ratios are calculated by normalizing the peak area of endogenous metabolites within tissue samples normalized to the internal standard Nilotinib_13C_2H3.

14.3- Phospho Abl (Pan-tyrosine) ELISA. PathScan® Phospho-c-Abl (panTyr) solid phase sandwich ELISA will be performed on human CSF and plasma. A c-Abl rabbit antibody will be coated on the microwells. 100 ul of CSF or plasma will be added to designated wells. After sample incubation for 2hrs at 37^0C, Bcr-Abl and c-Abl protein (phospho and nonphospho) will be captured by the coated antibody. Following extensive washing, a phospho-tyrosine (pan-tyrosine) detection antibody will be added to each well to detect captured tyrosine-phosphorylated Bcr-Abl and c-Abl protein. Samples will be incubated with detection antibody for 1hr at 37^0C. Following extensive washing, anti-mouse IgG, HRP-linked antibody will be added and incubated for 10 minutes at 37^0C to recognize the bound detection antibody. HRP substrate, TMB will be added to develop color. The magnitude of the absorbance for this developed color is proportional to the quantity of tyrosine-phosphorylated Bcr-Abl and c-Abl protein in the samples.

14.4- Cell death and exploratory biomarkers. NSE and S100B (glia and neurons), total Tau and phosphorylated Tau will be measured using Millipore ELISA at baseline and after 12 months Nilotinib treatment. Nilotinib may have a modulatory effect on myeloid cells (67), which can either proliferate and differentiate into peripheral macrophages or become myeloid-derived glia that cross the BBB and produce neurotrophic and/or inflammatory markers. Nilotinib may affect CNS glial progenitor cells. Our preliminary preclinical and clinical data show Nilotinib-induced alterations of peripheral and CNS inflammatory makers. Therefore, we will perform unbiased multiplex ELISA (Millipore) to profile a panel of 44 plasma and CSF markers, including interleukins (IL)- 1α&β, 2, 3, 4, 5, 6, 7, 8 (CXCL8), 9, 10, 12, 13, 15, 17 α, and chemokines (C-C) including, CXCL10, CCL2, CL7, CCL22, CCL3, CCL4, platelet-derived growth factor (PDGF)-AA, PDGF-AB/BB, CCL5, CX3CL1 (fractalkine), Tumor necrosis growth factor (TNF)-α, transforming growth factor (TGF)-α, and vascular endothelial growth factor (VEGF), GFAP, neurofilaments, glial fibrillary acidic protein (GFAP) and TDP-43.

14.5- Alpha-Synuclein ELISA. Solid phase alpha-Synuclein sandwich ELISA (Cat#SIG38974, Biolegend) will be performed on CSF and plasma. To avoid repetitive freeze and thaw cycles, immediately after LP and blood draws, 15 mL CSF and 5mL plasma will be aliquoted on ice into 0.5mL tubes and stored at -80°C. Fresh aliquots were used to perform or repeat ELISA. Total alpha- Synuclein rabbit monoclonal antibody (amino acids 118-123) will be coated on the microwells and 200µl CSF or plasma will be added to designated wells. CSF samples will be diluted 1:10 while plasma samples will be diluted 1:50. After overnight sample incubation at 2-8°C, alpha-Synuclein will be captured by the coated antibody. After washing, a biotinylated mouse monoclonal alpha-Synuclein (amino acids 103-107) detection antibody will be added to each well to detect the captured alpha-Synuclein (amino acids 118-123). Samples will be incubated with 50µl of detection antibody for 2hrs at room temperature. After washing, 200µl of streptavidin HRP will be added and incubated for 1hr at room temperature to recognize the bound biotinylated detection antibody. Samples will then washed and incubated with 100µl of chemiluminescent substrates. Plates will be shaken for 10-15 seconds and read immediately

by a luminometer. The magnitude of the luminescence is proportional to the quantity of alpha-Synuclein in the samples.

14.6- Homovanillic Acid ELISA. A 100µl CSF or plasma samples will be incubated with 100µl HRP-conjugate reagent and incubated for 1hr at 37°C using solid phase sandwich ELISA (MyBioSource, Cat# MBS064661). All samples at baseline and 6 months will be analyzed side-by-side using same reagents. After washing, 50µl of chromogen solution A and 50µl of chromogen solution B will be added to the solution and incubated for 15min at 37°C. The reaction will be stopped with 50µl stop solution and the optical density will be read at 450nM. The magnitude of the absorbance is proportional to the quantity of CSF and plasma HVA.

14.7- Total Tau and p-Tau181 measurement. Solid phase human total Tau sandwich ELISA (Invitrogen, Cat# KHB0042) and p-Tau181 (Invitrogen, Cat# KH0061) will be performed on CSF samples. All samples at baseline and 6 months will be analyzed side-by-side using same reagents. A monoclonal Tau or p-Tau181 capture antibodies will be coated onto micro-wells. 50µl of CSF will be added to each well, allowing human Tau or p-Tau181 antigen to bind to the immobilized capture antibody, and incubated for 2hrs at room temperature. After 2hr incubation, samples will be washed and incubated with 100µl of total Tau or p-Tau181 detection antibody and incubated for 1hr at room temperature. After washing, 100µl of HRP labeled anti-rabbit IgG will be added to each well and incubated for 30min at room temperature. Samples will be washed to remove all unbound enzyme and100µl TMB, a HRP substrate, will be added to develop color. The magnitude of the absorbance for this developed color is proportional to the quantity of CSF total Tau or p-Tau181.

14.8- Human Neurodegenerative Disease Panels. We will use a multiplex Xmap technology that uses magnetic microspheres internally coded with two fluorescent dyes to measure markers of neurodegeneration. All samples at baseline and 6 months will be analyzed side-by-side using same reagents. Through precise combinations of these two dyes, multiple proteins are measured within the sample. Each of these spheres is coated with a specific capture antibody. The capture antibody binds to the detection antibody and a reporter molecule, completing the reaction on the surface of the bead. 25µL of CSF or plasma will be incubated overnight at 4°C with 25µL of a mixed bead solution, containing human total Tau, pTau231 and NSE (Millipore, Cat#: HND1MAG-39K) or S100B, Aβ42, and Aβ40 (CSF Aβ40 is diluted 1:10). After washing, samples will be incubated with 25µL detection antibody solution for 1.5hrs at room temperature (Millipore, CAT#: HND4MAG-36K), 25µL of Streptavidin-Phycoerythrin will be added to each well containing the 25µl of detection antibody solution. Samples were then washed and suspended in 100µl of sheath fluid. Samples will be then run on MAGPIX with Xponent software. The Median Fluorescent Intensity (MFI) data will be analyzed using a 5-parameter logistic or spline curve-fitting method for calculating analyte concentrations in samples.

15- DATA COLLECTION

All data collected as part of this study will be entered into a secure data management site maintained by Georgetown University Information Service (UIS). All data will be stored on a UIS and IRB approved databases, including long-term storage on Amazon Web Service (WAS) and will be deposited on MEDUSA for an external and independent data analysis and monitoring. Data will be stored in EDC maintained by GUMC. This platform facilitates:

1. Capture of clinical and research data from neurologic patients for individual projects in a structured and secure system;
2. Aggregating and sharing uniform, de-identified and/or anonymized datasets for secondary analyses.

15.1- Role of Data Management

Data Management (DM) is responsible for the development, execution and supervision of plans, policies, programs, and practices that control, protect, deliver, and enhance the value of data and information assets.

All data will be managed in compliance with applicable Sponsor and regulatory requirements. Site personnel will collect, transcribe, correct, and transmit the data onto source documents, Case Report Forms (CRFs), and/or other forms used to report, track and record clinical research data. DM is responsible for developing, testing, and managing clinical data management activities.

15.2- Data Entry and Checks

The site personnel are instructed to enter information into the EDC. Data capture is the responsibility of the staff at the site under the supervision of the PIs. During the study, the PIs must maintain complete and accurate documentation for the study. The EDC provides password protection. An edit checking and data clarification process will be put in place to ensure accuracy of the data. Logic and range checks as well as more sophisticated rules may be built into the eCRFs to provide immediate error checking of the data entered. The system has the capability to automatically create electronic queries for forms that contain data that are out of range, out of window, missing or not calculated correctly.

15.3- Data Lock Process

The platform will have the ability to lock the project-specific visits to prevent any modification of data once the project is closed. Once this option is activated, every user will have Read-Only access to the data.

15.4- Data handling and record keeping

The PIs are responsible to ensure the accuracy, completeness, legibility, and timeliness of the data reported. Data reported in the eCRF derived from source documents should be consistent with the source documents and discrepancies should be explained.

15.5- Confidentiality

The EDC software and MEDUA databases and patient data reside on Georgetown University IRB-approved servers.. Physical and software access to the servers and security is provided by GUMC investigators.

15.6- Retention of Records

Research records will be retained in accordance with site IRB policies.

15.7- Publications

The PIs will be responsible for publications of results from this trial. Responsibilities will include the following:

- Analyze and interpret data gathered in this study, and write publications from these data.
- Submit manuscripts to selected journals and address peer reviewers' comments.
- Submit abstracts to selected meetings and present data at the meetings.
- Determine authorship on the basis of the Uniform Requirements for Manuscripts.

16 LITERATURE REFERENCES

1. Spillantini, M. G., Schmidt, M. L., Lee, V. M., Trojanowski, J. Q., Jakes, R., and Goedert, M. (1997) Alpha-synuclein in Lewy bodies. *Nature* **388**, 839-840
2. Spillantini, M. G., Crowther, R. A., Jakes, R., Hasegawa, M., and Goedert, M. (1998) alpha-Synuclein in filamentous inclusions of Lewy bodies from Parkinson's disease and dementia with lewy bodies. *Proceedings of the National Academy of Sciences of the United States of America* **95**, 6469-6473
3. Goedert, M. (2001) Alpha-synuclein and neurodegenerative diseases. *Nature reviews. Neuroscience* **2**, 492-501
4. Martin, E. R., Scott, W. K., Nance, M. A., Watts, R. L., Hubble, J. P., Koller, W. C., Lyons, K., Pahwa, R., Stern, M. B., Colcher, A., Hiner, B. C., Jankovic, J., Ondo, W. G., Allen, F. H., Jr., Goetz, C. G., Small, G. W., Masterman, D., Mastaglia, F., Laing, N. G., Stajich, J. M., Ribble, R. C., Booze, M. W., Rogala, A., Hauser, M. A., Zhang, F., Gibson, R. A., Middleton, L. T., Roses, A. D., Haines, J. L., Scott, B. L., Pericak-Vance, M. A., and Vance, J. M. (2001) Association of single-nucleotide polymorphisms of the tau gene with late-onset Parkinson disease. *JAMA* **286**, 2245-2250
5. Zabetian, C. P., Hutter, C. M., Factor, S. A., Nutt, J. G., Higgins, D. S., Griffith, A., Roberts, J. W., Leis, B. C., Kay, D. M., Yearout, D., Montimurro, J. S., Edwards, K. L., Samii, A., and Payami, H. (2007) Association analysis of MAPT H1 haplotype and subhaplotypes in Parkinson's disease. *Ann Neurol* **62**, 137-144
6. Healy, D. G., Abou-Sleiman, P. M., Lees, A. J., Casas, J. P., Quinn, N., Bhatia, K., Hingorani, A. D., and Wood, N. W. (2004) Tau gene and Parkinson's disease: a case-control study and meta-analysis. *J Neurol Neurosurg Psychiatry* **75**, 962-965
7. Maraganore, D. M., Hernandez, D. G., Singleton, A. B., Farrer, M. J., McDonnell, S. K., Hutton, M. L., Hardy, J. A., and Rocca, W. A. (2001) Case-Control study of the extended tau gene haplotype in Parkinson's disease. *Ann Neurol* **50**, 658-661
8. Wood, N. W. (1998) Genetic risk factors in Parkinson's disease. *Ann Neurol* **44**, S58-62
9. Gan-Or, Z., Dion, P. A., and Rouleau, G. A. (2015) Genetic perspective on the role of the Autophagy-Lysosome Pathway in Parkinson disease. *Autophagy*, 0
10. Winslow, A. R., and Rubinsztein, D. C. (2011) The Parkinson disease protein alpha-synuclein inhibits autophagy. *Autophagy* **7**, 429-431
11. Winslow, A. R., Chen, C. W., Corrochano, S., Acevedo-Arozena, A., Gordon, D. E., Peden, A. A., Lichtenberg, M., Menzies, F. M., Ravikumar, B., Imarisio, S., Brown, S., O'Kane, C. J., and Rubinsztein, D. C. (2010) alpha-Synuclein impairs macroautophagy: implications for Parkinson's disease. *J Cell Biol* **190**, 1023-1037
12. Lonskaya, I., Hebron, M. L., Algarzae, N. K., Desforges, N., and Moussa, C. E. (2012) Decreased parkin solubility is associated with impairment of autophagy in the nigrostriatum of sporadic Parkinson's disease. *Neuroscience*
13. Hebron, M. L., Lonskaya, I., and Moussa, C. E. (2013) Nilotinib reverses loss of dopamine neurons and improves motor behavior via autophagic degradation of alpha-synuclein in Parkinson's disease models. *Human molecular genetics* **22**, 3315-3328
14. Boland, B., Kumar, A., Lee, S., Platt, F. M., Wegiel, J., Yu, W. H., and Nixon, R. A. (2008) Autophagy induction and autophagosome clearance in neurons: relationship to autophagic pathology in Alzheimer's disease. *J Neurosci* **28**, 6926-6937
15. Kegel, K. B., Kim, M., Sapp, E., McIntyre, C., Castano, J. G., Aronin, N., and DiFiglia, M. (2000) Huntingtin expression stimulates endosomal-lysosomal activity, endosome tubulation, and autophagy. *J Neurosci* **20**, 7268-7278

16. Nixon, R. A., Wegiel, J., Kumar, A., Yu, W. H., Peterhoff, C., Cataldo, A., and Cuervo, A. M. (2005) Extensive involvement of autophagy in Alzheimer disease: an immuno-electron microscopy study. *J Neuropathol Exp Neurol* **64**, 113-122
17. Ravikumar, B., Duden, R., and Rubinsztein, D. C. (2002) Aggregate-prone proteins with polyglutamine and polyalanine expansions are degraded by autophagy. *Hum Mol Genet* **11**, 1107-1117
18. Sabatini, D. M. (2006) mTOR and cancer: insights into a complex relationship. *Nat Rev Cancer* **6**, 729-734
19. Stefanis, L., Larsen, K. E., Rideout, H. J., Sulzer, D., and Greene, L. A. (2001) Expression of A53T mutant but not wild-type alpha-synuclein in PC12 cells induces alterations of the ubiquitin-dependent degradation system, loss of dopamine release, and autophagic cell death. *J Neurosci* **21**, 9549-9560
20. Webb, J. L., Ravikumar, B., Atkins, J., Skepper, J. N., and Rubinsztein, D. C. (2003) Alpha-Synuclein is degraded by both autophagy and the proteasome. *J Biol Chem* **278**, 25009-25013
21. Nixon, R. A. (2013) The role of autophagy in neurodegenerative disease. *Nature medicine* **19**, 983-997
22. Salomoni, P., and Calabretta, B. (2009) Targeted therapies and autophagy: new insights from chronic myeloid leukemia. *Autophagy* **5**, 1050-1051
23. Lonskaya, I., Hebron, M. L., Desforges, N. M., Franjie, A., and Moussa, C. E. (2013) Tyrosine kinase inhibition increases functional parkin-Beclin-1 interaction and enhances amyloid clearance and cognitive performance. *EMBO Mol Med* **5**, 1247-1262
24. Lonskaya, I., Hebron, M. L., Desforges, N. M., Schachter, J. B., and Moussa, C. E. (2014) Nilotinib-induced autophagic changes increase endogenous parkin level and ubiquitination, leading to amyloid clearance. *Journal of molecular medicine* **92**, 373-386
25. Lonskaya, I., Hebron, M., Chen, W., Schachter, J., and Moussa, C. (2014) Tau deletion impairs intracellular beta-amyloid-42 clearance and leads to more extracellular plaque deposition in gene transfer models. *Molecular neurodegeneration* **9**, 46
26. Lonskaya, I., Desforges, N. M., Hebron, M. L., and Moussa, C. E. (2013) Ubiquitination increases parkin activity to promote autophagic alpha-synuclein clearance. *PloS one* **8**, e83914
27. Deremer, D. L., Ustun, C., and Natarajan, K. (2008) Nilotinib: a second-generation tyrosine kinase inhibitor for the treatment of chronic myelogenous leukemia. *Clin Ther* **30**, 1956-1975
28. Skorski, T. (2011) BCR-ABL1 kinase: hunting an elusive target with new weapons. *Chem Biol* **18**, 1352-1353
29. Mahon, F. X., Hayette, S., Lagarde, V., Belloc, F., Turcq, B., Nicolini, F., Belanger, C., Manley, P. W., Leroy, C., Etienne, G., Roche, S., and Pasquet, J. M. (2008) Evidence that resistance to nilotinib may be due to BCR-ABL, Pgp, or Src kinase overexpression. *Cancer Res* **68**, 9809-9816
30. Hebron, M. L., Lonskaya, I., Olopade, P., Selby, S. T., Pagan, F., and Moussa, C. E. (2014) Tyrosine Kinase Inhibition Regulates Early Systemic Immune Changes and Modulates the Neuroimmune Response in alpha-Synucleinopathy. *J Clin Cell Immunol* **5**, 259
31. Wenqiang, C., Lonskaya, I., Hebron, M. L., Ibrahim, Z., Olszewski, R. T., Neale, J. H., and Moussa, C. E. (2014) Parkin-mediated reduction of nuclear and soluble TDP-43 reverses behavioral decline in symptomatic mice. *Human molecular genetics* **23**, 4960-4969
32. Hebron, M., Lonskaya, I., and Moussa, C. E. (2013) Nilotinib reverses loss of dopamine neurons and improves motor behavior via autophagic degradation of α-synuclein in Parkinson's disease models. *Hum. Mol. Genet.* **22**, 3315-3328

33. Moussa, C. E. (2015) Parkin Is Dispensable for Mitochondrial Function, but Its Ubiquitin Ligase Activity Is Critical for Macroautophagy and Neurotransmitters: Therapeutic Potential beyond Parkinson's Disease. *Neuro-degenerative diseases*
34. Lonskaya, I., Hebron, M. L., Selby, S. T., Turner, R. S., and Moussa, C. E. (2015) Nilotinib and bosutinib modulate pre-plaque alterations of blood immune markers and neuro-inflammation in Alzheimer's disease models. *Neuroscience* **304**, 316-327
35. Karuppagounder, S. S., Brahmachari, S., Lee, Y., Dawson, V. L., Dawson, T. M., and Ko, H. S. (2014) The c-Abl inhibitor, nilotinib, protects dopaminergic neurons in a preclinical animal model of Parkinson's disease. *Scientific reports* **4**, 4874
36. Mahul-Mellier, A. L., Fauvet, B., Gysbers, A., Dikiy, I., Oueslati, A., Georgeon, S., Lamontanara, A. J., Bisquertt, A., Eliezer, D., Masliah, E., Halliday, G., Hantschel, O., and Lashuel, H. A. (2014) c-Abl phosphorylates alpha-synuclein and regulates its degradation: implication for alpha-synuclein clearance and contribution to the pathogenesis of Parkinson's disease. *Human molecular genetics* **23**, 2858-2879
37. Mollenhauer, B., Locascio, J. J., Schulz-Schaeffer, W., Sixel-Doring, F., Trenkwalder, C., and Schlossmacher, M. G. (2011) alpha-Synuclein and tau concentrations in cerebrospinal fluid of patients presenting with parkinsonism: a cohort study. *Lancet Neurol* **10**, 230-240
38. Parnetti, L., Chiasserini, D., Bellomo, G., Giannandrea, D., De Carlo, C., Qureshi, M. M., Ardah, M. T., Varghese, S., Bonanni, L., Borroni, B., Tambasco, N., Eusebi, P., Rossi, A., Onofrj, M., Padovani, A., Calabresi, P., and El-Agnaf, O. (2011) Cerebrospinal fluid Tau/alpha-synuclein ratio in Parkinson's disease and degenerative dementias. *Mov Disord* **26**, 1428-1435
39. Tateno, F., Sakakibara, R., Kawai, T., Kishi, M., and Murano, T. (2012) Alpha-synuclein in the cerebrospinal fluid differentiates synucleinopathies (Parkinson Disease, dementia with Lewy bodies, multiple system atrophy) from Alzheimer disease. *Alzheimer Dis Assoc Disord* **26**, 213-216
40. Kantarjian, H., Sawyers, C., Hochhaus, A., Guilhot, F., Schiffer, C., Gambacorti-Passerini, C., Niederwieser, D., Resta, D., Capdeville, R., Zoellner, U., Talpaz, M., Druker, B., Goldman, J., O'Brien, S. G., Russell, N., Fischer, T., Ottmann, O., Cony-Makhoul, P., Facon, T., Stone, R., Miller, C., Tallman, M., Brown, R., Schuster, M., Loughran, T., Gratwohl, A., Mandelli, F., Saglio, G., Lazzarino, M., Russo, D., Baccarani, M., and Morra, E. (2002) Hematologic and cytogenetic responses to imatinib mesylate in chronic myelogenous leukemia. *N Engl J Med* **346**, 645-652
41. Qiu, Z., Cang, Y., and Goff, S. P. (2010) c-Abl tyrosine kinase regulates cardiac growth and development. *Proc Natl Acad Sci U S A* **107**, 1136-1141
42. Li, B., Boast, S., de los Santos, K., Schieren, I., Quiroz, M., Teitelbaum, S. L., Tondravi, M. M., and Goff, S. P. (2000) Mice deficient in Abl are osteoporotic and have defects in osteoblast maturation. *Nat Genet* **24**, 304-308
43. Schwartzberg, P. L., Stall, A. M., Hardin, J. D., Bowdish, K. S., Humaran, T., Boast, S., Harbison, M. L., Robertson, E. J., and Goff, S. P. (1991) Mice homozygous for the ablm1 mutation show poor viability and depletion of selected B and T cell populations. *Cell* **65**, 1165-1175
44. Tybulewicz, V. L., Crawford, C. E., Jackson, P. K., Bronson, R. T., and Mulligan, R. C. (1991) Neonatal lethality and lymphopenia in mice with a homozygous disruption of the c-abl proto-oncogene. *Cell* **65**, 1153-1163
45. Bakhsheshian, J., Wei, B. R., Chang, K. E., Shukla, S., Ambudkar, S. V., Simpson, R. M., Gottesman, M. M., and Hall, M. D. (2013) Bioluminescent imaging of drug efflux at the blood-brain barrier mediated by the transporter ABCG2. *Proceedings of the National Academy of Sciences of the United States of America* **110**, 20801-20806

46. Giasson, B. I., Duda, J. E., Quinn, S. M., Zhang, B., Trojanowski, J. Q., and Lee, V. M. (2002) Neuronal alpha-synucleinopathy with severe movement disorder in mice expressing A53T human alpha-synuclein. *Neuron* **34**, 521-533
47. Lonskaya, I., Shekoyan, A. R., Hebron, M. L., Desforges, N., Algarzae, N. K., and Moussa, C. E. (2012) Diminished Parkin Solubility and Co-Localization with Intraneuronal Amyloid-beta are Associated with Autophagic Defects in Alzheimer's Disease. *J Alzheimers Dis*
48. Majbour, N. K., Vaikath, N. N., Eusebi, P., Chiasserini, D., Ardah, M., Varghese, S., Haque, M. E., Tokuda, T., Auinger, P., Calabresi, P., Parnetti, L., and El-Agnaf, O. M. (2016) Longitudinal changes in CSF alpha-synuclein species reflect Parkinson's disease progression. *Mov Disord*
49. Majbour, N. K., Vaikath, N. N., van Dijk, K. D., Ardah, M. T., Varghese, S., Vesterager, L. B., Montezinho, L. P., Poole, S., Safieh-Garabedian, B., Tokuda, T., Teunissen, C. E., Berendse, H. W., van de Berg, W. D., and El-Agnaf, O. M. (2016) Oligomeric and phosphorylated alpha-synuclein as potential CSF biomarkers for Parkinson's disease. *Molecular neurodegeneration* **11**, 7
50. van Erp, N. P., Gelderblom, H., and Guchelaar, H. J. (2009) Clinical pharmacokinetics of tyrosine kinase inhibitors. *Cancer Treat Rev* **35**, 692-706
51. Kantarjian, H. M., Giles, F., Gattermann, N., Bhalla, K., Alimena, G., Palandri, F., Ossenkoppele, G. J., Nicolini, F. E., O'Brien, S. G., Litzow, M., Bhatia, R., Cervantes, F., Haque, A., Shou, Y., Resta, D. J., Weitzman, A., Hochhaus, A., and le Coutre, P. (2007) Nilotinib (formerly AMN107), a highly selective BCR-ABL tyrosine kinase inhibitor, is effective in patients with Philadelphia chromosome-positive chronic myelogenous leukemia in chronic phase following imatinib resistance and intolerance. *Blood* **110**, 3540-3546
52. de Lavallade, H., Apperley, J. F., Khorashad, J. S., Milojkovic, D., Reid, A. G., Bua, M., Szydlo, R., Olavarria, E., Kaeda, J., Goldman, J. M., and Marin, D. (2008) Imatinib for newly diagnosed patients with chronic myeloid leukemia: incidence of sustained responses in an intention-to-treat analysis. *J Clin Oncol* **26**, 3358-3363
53. Polymenidou, M., and Cleveland, D. W. (2012) Prion-like spread of protein aggregates in neurodegeneration. *The Journal of experimental medicine* **209**, 889-893
54. Ciechanover, A., and Kwon, Y. T. (2015) Degradation of misfolded proteins in neurodegenerative diseases: therapeutic targets and strategies. *Experimental & molecular medicine* **47**, e147
55. Mellman, I. (1996) Endocytosis and molecular sorting. *Annual review of cell and developmental biology* **12**, 575-625
56. Jerram, A. H., Smith, P. F., and Darlington, C. L. (1996) A dose-response analysis of the behavioral effects of (+)MK-801 in guinea pig: comparison with CPP. *Pharmacology, biochemistry, and behavior* **53**, 799-807
57. Luzio, J. P., Rous, B. A., Bright, N. A., Pryor, P. R., Mullock, B. M., and Piper, R. C. (2000) Lysosome-endosome fusion and lysosome biogenesis. *Journal of cell science* **113 (Pt 9)**, 1515-1524
58. Kettenmann, H., Hanisch, U. K., Noda, M., and Verkhratsky, A. (2011) Physiology of microglia. *Physiol Rev* **91**, 461-553
59. Yang, Z. J., Chee, C. E., Huang, S., and Sinicrope, F. A. (2011) The role of autophagy in cancer: therapeutic implications. *Mol Cancer Ther* **10**, 1533-1541
60. Macintosh, R. L., and Ryan, K. M. (2013) Autophagy in tumour cell death. *Seminars in cancer biology* **23**, 344-351
61. Crighton, D., Wilkinson, S., O'Prey, J., Syed, N., Smith, P., Harrison, P. R., Gasco, M., Garrone, O., Crook, T., and Ryan, K. M. (2006) DRAM, a p53-induced modulator of autophagy, is critical for apoptosis. *Cell* **126**, 121-134

62. Yee, K. S., Wilkinson, S., James, J., Ryan, K. M., and Vousden, K. H. (2009) PUMA- and Bax-induced autophagy contributes to apoptosis. *Cell death and differentiation* **16**, 1135-1145
63. Nakada, M., Kita, D., Teng, L., Pyko, I. V., Watanabe, T., Hayashi, Y., and Hamada, J. (2013) Receptor tyrosine kinases: principles and functions in glioma invasion. *Advances in experimental medicine and biology* **986**, 143-170
64. Cadena, D. L., and Gill, G. N. (1992) Receptor tyrosine kinases. *FASEB J* **6**, 2332-2337
65. Jabbour, E., Kantarjian, H., and Cortes, J. (2015) Use of second- and third-generation tyrosine kinase inhibitors in the treatment of chronic myeloid leukemia: an evolving treatment paradigm. *Clin Lymphoma Myeloma Leuk* **15**, 323-334
66. Holyoake, T. L., and Helgason, G. V. (2015) Do we need more drugs for chronic myeloid leukemia? *Immunol Rev* **263**, 106-123
67. Zhang, H., and Li, S. (2013) Molecular mechanisms for survival regulation of chronic myeloid leukemia stem cells. *Protein Cell* **4**, 186-196
68. Hong, Z., Shi, M., Chung, K. A., Quinn, J. F., Peskind, E. R., Galasko, D., Jankovic, J., Zabetian, C. P., Leverenz, J. B., Baird, G., Montine, T. J., Hancock, A. M., Hwang, H., Pan, C., Bradner, J., Kang, U. J., Jensen, P. H., and Zhang, J. (2010) DJ-1 and alpha-synuclein in human cerebrospinal fluid as biomarkers of Parkinson's disease. *Brain* **133**, 713-726
69. Ohrfelt, A., Grognet, P., Andreasen, N., Wallin, A., Vanmechelen, E., Blennow, K., and Zetterberg, H. (2009) Cerebrospinal fluid alpha-synuclein in neurodegenerative disorders-a marker of synapse loss? *Neurosci Lett* **450**, 332-335
70. Kasuga, K., Tokutake, T., Ishikawa, A., Uchiyama, T., Tokuda, T., Onodera, O., Nishizawa, M., and Ikeuchi, T. (2010) Differential levels of alpha-synuclein, beta-amyloid42 and tau in CSF between patients with dementia with Lewy bodies and Alzheimer's disease. *J Neurol Neurosurg Psychiatry* **81**, 608-610
71. Mollenhauer, B., Trautmann, E., Otte, B., Ng, J., Spreer, A., Lange, P., Sixel-Doring, F., Hakimi, M., Vonsattel, J. P., Nussbaum, R., Trenkwalder, C., and Schlossmacher, M. G. (2012) alpha-Synuclein in human cerebrospinal fluid is principally derived from neurons of the central nervous system. *Journal of neural transmission* **119**, 739-746
72. Mollenhauer, B., Cullen, V., Kahn, I., Krastins, B., Outeiro, T. F., Pepivani, I., Ng, J., Schulz-Schaeffer, W., Kretzschmar, H. A., McLean, P. J., Trenkwalder, C., Sarracino, D. A., Vonsattel, J. P., Locascio, J. J., El-Agnaf, O. M., and Schlossmacher, M. G. (2008) Direct quantification of CSF alpha-synuclein by ELISA and first cross-sectional study in patients with neurodegeneration. *Experimental neurology* **213**, 315-325
73. Mollenhauer, B., Trautmann, E., Taylor, P., Manninger, P., Sixel-Doring, F., Ebentheuer, J., Trenkwalder, C., and Schlossmacher, M. G. (2013) Total CSF alpha-synuclein is lower in de novo Parkinson patients than in healthy subjects. *Neurosci Lett* **532**, 44-48
74. Lleo, A., Cavedo, E., Parnetti, L., Vanderstichele, H., Herukka, S. K., Andreasen, N., Ghidoni, R., Lewczuk, P., Jeromin, A., Winblad, B., Tsolaki, M., Mroczko, B., Visser, P. J., Santana, I., Svenningsson, P., Blennow, K., Aarsland, D., Molinuevo, J. L., Zetterberg, H., and Mollenhauer, B. (2015) Cerebrospinal fluid biomarkers in trials for Alzheimer and Parkinson diseases. *Nature reviews. Neurology* **11**, 41-55
75. Kang, J. H., Irwin, D. J., Chen-Plotkin, A. S., Siderowf, A., Caspell, C., Coffey, C. S., Waligorska, T., Taylor, P., Pan, S., Frasier, M., Marek, K., Kieburtz, K., Jennings, D., Simuni, T., Tanner, C. M., Singleton, A., Toga, A. W., Chowdhury, S., Mollenhauer, B., Trojanowski, J. Q., Shaw, L. M., and Parkinson's Progression Markers, I. (2013) Association of cerebrospinal fluid beta-amyloid 1-42, T-tau, P-tau181, and alpha-synuclein levels with clinical features of drug-naive patients with early Parkinson disease. *JAMA Neurol* **70**, 1277-1287

76. Zubenko, G. S., Marquis, J. K., Volicer, L., Direnfeld, L. K., Langlais, P. J., and Nixon, R. A. (1986) Cerebrospinal fluid levels of angiotensin-converting enzyme, acetylcholinesterase, and dopamine metabolites in dementia associated with Alzheimer's disease and Parkinson's disease: a correlative study. *Biol Psychiatry* **21**, 1365-1381
77. Goldstein, D. S., Holmes, C., and Sharabi, Y. (2012) Cerebrospinal fluid biomarkers of central catecholamine deficiency in Parkinson's disease and other synucleinopathies. *Brain* **135**, 1900-1913

17- APPENDIX

- PDQ-39
- MoCA
- C-SSRS baseline
- C-SSRS since last visit
- GSRS
- GDS
- MDS-UPDRS

PDQ-39 QUESTIONNAIRE

Please complete the following

Please tick one box for each question

Due to having Parkinson's disease, how often during the last month have you....		Never	Occasionally	Sometimes	Often	Always or cannot do at all
1	Had difficulty doing the leisure activities which you would like to do?	☐	☐	☐	☐	☐
2	Had difficulty looking after your home, e.g. DIY, housework, cooking?	☐	☐	☐	☐	☐
3	Had difficulty carrying bags of shopping?	☐	☐	☐	☐	☐
4	Had problems walking half a mile?	☐	☐	☐	☐	☐
5	Had problems walking 100 yards?	☐	☐	☐	☐	☐
6	Had problems getting around the house as easily as you would like?	☐	☐	☐	☐	☐
7	Had difficulty getting around in public?	☐	☐	☐	☐	☐
8	Needed someone else to accompany you when you went out?	☐	☐	☐	☐	☐
9	Felt frightened or worried about falling over in public?	☐	☐	☐	☐	☐
10	Been confined to the house more than you would like?	☐	☐	☐	☐	☐
11	Had difficulty washing yourself?	☐	☐	☐	☐	☐
12	Had difficulty dressing yourself?	☐	☐	☐	☐	☐
13	Had problems doing up your shoe laces?	☐	☐	☐	☐	☐

*Please check that you have ticked **one box for each question** before going on to the next page*

Due to having Parkinson's disease, how often during the last month have you....

Please tick one box for each question

		Never	Occasionally	Sometimes	Often	Always or cannot do at all
14	Had problems writing clearly?	☐	☐	☐	☐	☐
15	Had difficulty cutting up your food?	☐	☐	☐	☐	☐
16	Had difficulty holding a drink without spilling it?	☐	☐	☐	☐	☐
17	Felt depressed?	☐	☐	☐	☐	☐
18	Felt isolated and lonely?	☐	☐	☐	☐	☐
19	Felt weepy or tearful?	☐	☐	☐	☐	☐
20	Felt angry or bitter?	☐	☐	☐	☐	☐
21	Felt anxious?	☐	☐	☐	☐	☐
22	Felt worried about your future?	☐	☐	☐	☐	☐
23	Felt you had to conceal your Parkinson's from people?	☐	☐	☐	☐	☐
24	Avoided situations which involve eating or drinking in public?	☐	☐	☐	☐	☐
25	Felt embarrassed in public due to having Parkinson's disease?	☐	☐	☐	☐	☐
26	Felt worried by other people's reaction to you?	☐	☐	☐	☐	☐
27	Had problems with your close personal relationships?	☐	☐	☐	☐	☐
28	Lacked support in the ways you need from your spouse or partner?	☐	☐	☐	☐	☐
	If you do not have a spouse or partner tick here		☐			
29	Lacked support in the ways you need from your family or close friends?	☐	☐	☐	☐	☐

*Please check that you have ticked **one box for each question** before going on to the next page*

Due to having Parkinson's disease, how often during the last month have you....	***Please tick one box for each question***				
	Never	Occasionally	Sometimes	Often	Always
30 Unexpectedly fallen asleep during the day?	☐	☐	☐	☐	☐
31 Had problems with your concentration, e.g. when reading or watching TV?	☐	☐	☐	☐	☐
32 Felt your memory was bad?	☐	☐	☐	☐	☐
33 Had distressing dreams or hallucinations?	☐	☐	☐	☐	☐
34 Had difficulty with your speech?	☐	☐	☐	☐	☐
35 Felt unable to communicate with people properly?	☐	☐	☐	☐	☐
36 Felt ignored by people?	☐	☐	☐	☐	☐
37 Had painful muscle cramps or spasms?	☐	☐	☐	☐	☐
38 Had aches and pains in your joints or body?	☐	☐	☐	☐	☐
39 Felt unpleasantly hot or cold?	☐	☐	☐	☐	☐

Please check that you have ticked ***one box for each question*** *before going on to the next page*

Thank you for completing the PDQ 39 questionnaire

NAME :
Education : Date of birth :
Sex : DATE :

MONTREAL COGNITIVE ASSESSMENT (MOCA)

Version 7.1 Original Version

			POINTS
**VISUOSPATIAL / EXECUTIVE**	Copy cube	Draw CLOCK (Ten past eleven) (3 points)	

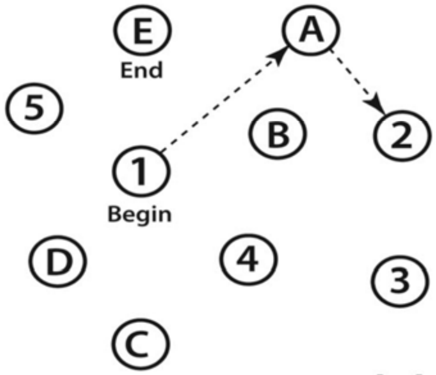

[ ]

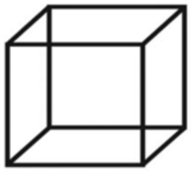

[ ]

[ ] Contour [ ] Numbers [ ] Hands ___/5

NAMING

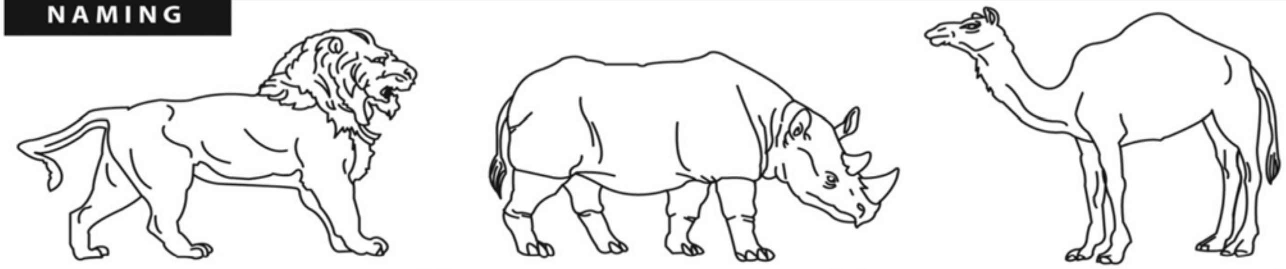

[ ] [ ] [ ] ___/3

MEMORY Read list of words, subject must repeat them. Do 2 trials, even if 1st trial is successful. Do a recall after 5 minutes.

	FACE	VELVET	CHURCH	DAISY	RED
1st trial					
2nd trial					

No points

ATTENTION Read list of digits (1 digit/ sec.). Subject has to repeat them in the forward order [ ] 2 1 8 5 4
Subject has to repeat them in the backward order [ ] 7 4 2 ___/2

Read list of letters. The subject must tap with his hand at each letter A. No points if ≥ 2 errors
[ ] F B A C M N A A J K L B A F A K D E A A A J A M O F A A B ___/1

Serial 7 subtraction starting at 100 [ ] 93 [ ] 86 [ ] 79 [ ] 72 [ ] 65
4 or 5 correct subtractions: **3 pts**, 2 or 3 correct: **2 pts**, 1 correct: **1 pt**, 0 correct: **0 pt** ___/3

LANGUAGE Repeat : I only know that John is the one to help today. [ ]
The cat always hid under the couch when dogs were in the room. [ ] ___/2

Fluency / Name maximum number of words in one minute that begin with the letter F [ ] _____ (N ≥ 11 words) ___/1

ABSTRACTION Similarity between e.g. banana - orange = fruit [ ] train – bicycle [ ] watch - ruler ___/2

**DELAYED RECALL**	Has to recall words **WITH NO CUE**	FACE [ ]	VELVET [ ]	CHURCH [ ]	DAISY [ ]	RED [ ]	Points for UNCUED recall only	___/5
**Optional**	Category cue							
	Multiple choice cue							

ORIENTATION [ ] Date [ ] Month [ ] Year [ ] Day [ ] Place [ ] City ___/6

© Z.Nasreddine MD www.mocatest.org Normal ≥26 / 30 TOTAL ___/30

Add 1 point if ≤ 12 yr edu

Administered by: ______________________________

COLUMBIA-SUICIDE SEVERITY RATING SCALE - *BASELINE*

COLUMBIA-SUICIDE SEVERITY
RATING SCALE
(C-SSRS)
Baseline
Version 1/14/09

Posner, K.; Brent, D.; Lucas, C.; Gould, M.; Stanley, B.; Brown, G.; Fisher, P.; Zelazny, J.; Burke, A.; Oquendo, M.; Mann, J.

Disclaimer:
This scale is intended to be used by individuals who have received training in its administration. The questions contained in the Columbia-Suicide Severity Rating Scale are suggested probes. Ultimately, the determination of the presence of suicidal ideation or behavior depends on the judgment of the individual administering the scale.
Definitions of behavioral suicidal events in this scale are based on those used in ***The Columbia Suicide History Form****, developed by John Mann, MD and Maria Oquendo, MD, Conte Center for the Neuroscience of Mental Disorders (CCNMD), New York State Psychiatric Institute, 1051 Riverside Drive, New York, NY, 10032. (Oquendo M. A., Halberstam B. & Mann J. J., Risk factors for suicidal behavior: utility and limitations of research instruments. In M.B. First [Ed.]* Standardized Evaluation in Clinical Practice*, pp. 103 -130, 2003.)*
For reprints of the C-SSRS contact Kelly Posner, Ph.D., New York State Psychiatric Institute, 1051 Riverside Drive, New York, New York, 10032; inquiries and training requirements contact posnerk@nyspi.columbia.edu

© 2008 The Research Foundation for Mental Hygiene, Inc.

SUICIDAL IDEATION	
Ask questions 1 and 2. If both are negative, proceed to "Suicidal Behavior" section. If the answer to question 2 is "yes", ask questions 3, 4 and 5. If the answer to question 1 and/or 2 is "yes", complete "Intensity of Ideation" section below.	**Lifetime: Time He/She Felt Most Suicidal**
**1. Wish to be Dead**<br>Subject endorses thoughts about a wish to be dead or not alive anymore, or wish to fall asleep and not wake up.<br>***Have you wished you were dead or wished you could go to sleep and not wake up?***<br>If yes, describe:	**Yes No**<br>□ □
**2. Non-Specific Active Suicidal Thoughts**<br>General, non-specific thoughts of wanting to end one's life/commit suicide (e.g., *"I've thought about killing myself"*) without thoughts of ways to kill oneself/associated methods, intent, or plan.<br>***Have you actually had any thoughts of killing yourself?***<br>If yes, describe:	**Yes No**<br>□ □
**3. Active Suicidal Ideation with Any Methods (Not Plan) without Intent to Act**<br>Subject endorses thoughts of suicide and has thought of at least one method during the assessment period. This is different than a specific plan with time, place or method details worked out (e.g., thought of method to kill self but not a specific plan). Includes person who would say, *"I thought about taking an overdose but I never made a specific plan as to when, where or how I would actually do it…and I would never go through with it."*<br>***Have you been thinking about how you might do this?***<br>If yes, describe:	**Yes No**<br>□ □
**4. Active Suicidal Ideation with Some Intent to Act, without Specific Plan**<br>Active suicidal thoughts of killing oneself and subject reports having some intent to act on such thoughts, as opposed to *"I have the thoughts but I definitely will not do anything about them."*<br>***Have you had these thoughts and had some intention of acting on them?***<br>If yes, describe:	**Yes No**<br>□ □
**5. Active Suicidal Ideation with Specific Plan and Intent**<br>Thoughts of killing oneself with details of plan fully or partially worked out and subject has some intent to carry it out.<br>***Have you started to work out or worked out the details of how to kill yourself? Do you intend to carry out this plan?***<br>If yes, describe:	**Yes No**<br>□ □
INTENSITY OF IDEATION	

The following features should be rated with respect to the most severe type of ideation (i.e., 1-5 from above, with 1 being the least severe and 5 being the most severe). Ask about time he/she was feeling the most suicidal.<br><br>***Most Severe Ideation:*** _____<br>____<br>***Type # (1-5)***<br>***Description of Ideation***	Most Severe
**Frequency**<br>***How many times have you had these thoughts?***<br>(1) Less than once a week (2) Once a week (3) 2-5 times in week (4) Daily or almost daily (5) Many times each day	____
**Duration**<br>***When you have the thoughts, how long do they last?***<br>(1) Fleeting - few seconds or minutes (4) 4-8 hours/most of day<br>(2) Less than 1 hour/some of the time (5) More than 8 hours/persistent or continuous<br>(3) 1-4 hours/a lot of time	____
**Controllability**<br>***Could/can you stop thinking about killing yourself or wanting to die if you want to?***<br>(1) Easily able to control thoughts (4) Can control thoughts with a lot of difficulty<br>(2) Can control thoughts with little difficulty (5) Unable to control thoughts<br>(3) Can control thoughts with some difficulty (0) Does not attempt to control thoughts	____
**Deterrents**<br>***Are there things - anyone or anything (e.g., family, religion, pain of death) - that stopped you from wanting to die or acting on thoughts of committing suicide?***<br>(1) Deterrents definitely stopped you from attempting suicide (4) Deterrents most likely did not stop you<br>(2) Deterrents probably stopped you (5) Deterrents definitely did not stop you<br>(3) Uncertain that deterrents stopped you (0) Does not apply	____
**Reasons for Ideation**<br>***What sort of reasons did you have for thinking about wanting to die or killing yourself? Was it to end the pain or stop the way you were feeling (in other words you couldn't go on living with this pain or how you were feeling) or was it to get attention, revenge or a reaction from others? Or both?***<br>(1) Completely to get attention, revenge or a reaction from others (4) Mostly to end or stop the pain (you couldn't go on<br>(2) Mostly to get attention, revenge or a reaction from others living with the pain or how you were feeling)<br>(3) Equally to get attention, revenge or a reaction from others (5) Completely to end or stop the pain (you couldn't go on	____

and to end/stop the pain. pain or how you were feeling)	living with the<br>(0) Does not apply	

© 2008 Research Foundation for Mental Hygiene, Inc. C-SSRS—Baseline (Version 1/14/09)

SUICIDAL BEHAVIOR<br>*(Check all that apply, so long as these are separate events; must ask about all types)*	**Lifetime**
**Actual Attempt:**<br>A potentially self-injurious act committed with at least some wish to die, *as a result of act.* Behavior was in part thought of as method to kill oneself. Intent does not have to be 100%. If there is ***any*** intent/desire to die associated with the act, then it can be considered an actual suicide attempt. ***There does not have to be any injury or harm***, just the potential for injury or harm. If person pulls trigger while gun is in mouth but gun is broken so no injury results, this is considered an attempt.<br>Inferring Intent: Even if an individual denies intent/wish to die, it may be inferred clinically from the behavior or circumstances. For example, a highly lethal act that is clearly not an accident so no other intent but suicide can be inferred (e.g., gunshot to head, jumping from window of a high floor/story). Also, if someone denies intent to die, but they thought that what they did could be lethal, intent may be inferred.<br>***Have you made a suicide attempt?***<br>***Have you done anything to harm yourself?***<br>***Have you done anything dangerous where you could have died?***<br>***What did you do?***<br>***Did you______ as a way to end your life?***<br>***Did you want to die (even a little) when you_____?***<br>***Were you trying to end your life when you _____?***<br>***Or did you think it was possible you could have died from_____?***<br>***Or did you do it purely for other reasons / without ANY intention of killing yourself (like to relieve stress, feel better, get sympathy, or get something else to happen)?*** (Self-Injurious Behavior without suicidal intent)<br>If yes, describe:<br><br>**Has subject engaged in Non-Suicidal Self-Injurious Behavior?**	**Yes No**<br>□ □<br><br>Total # of Attempts<br>_______<br><br>**Yes No**<br>□ □
**Interrupted Attempt:**<br>When the person is interrupted (by an outside circumstance) from starting the potentially self-injurious act *(if not for that, actual attempt would have occurred).*<br>Overdose: Person has pills in hand but is stopped from ingesting. Once they ingest any pills, this becomes an attempt rather than an interrupted attempt. Shooting: Person has gun pointed toward self, gun is taken away by someone else, or is somehow prevented from pulling trigger. Once they pull the trigger, even if the gun fails to fire, it is an attempt. Jumping: Person is poised to jump, is grabbed and taken down from ledge. Hanging: Person has noose around neck but has not yet started to hang - is stopped from doing so.<br>***Has there been a time when you started to do something to end your life but someone or something stopped you before you actually did anything?***	**Yes No**<br>□ □<br><br>Total # of interrupted<br>_______

<table>
<tr><td colspan="4">If yes, describe:</td><td></td></tr>
<tr><td colspan="4">Aborted Attempt:
When person begins to take steps toward making a suicide attempt, but stops themselves before they actually have engaged in any self-destructive behavior. Examples are similar to interrupted attempts, except that the individual stops him/herself, instead of being stopped by something else.
Has there been a time when you started to do something to try to end your life but you stopped yourself before you actually did anything?
If yes, describe:</td><td>Yes No
□ □

Total # of aborted
______</td></tr>
<tr><td colspan="4">Preparatory Acts or Behavior:
Acts or preparation towards imminently making a suicide attempt. This can include anything beyond a verbalization or thought, such as assembling a specific method (e.g., buying pills, purchasing a gun) or preparing for one's death by suicide (e.g., giving things away, writing a suicide note).
Have you taken any steps towards making a suicide attempt or preparing to kill yourself (such as collecting pills, getting a gun, giving valuables away or writing a suicide note)?
If yes, describe:</td><td>Yes No
□ □</td></tr>
<tr><td colspan="4">Suicidal Behavior:
Suicidal behavior was present during the assessment period?</td><td>Yes No
□ □</td></tr>
<tr><td>Answer for Actual Attempts Only</td><td>Most Recent Attempt Date:</td><td>Most Lethal Attempt Date:</td><td colspan="2">Initial/First Attempt Date:</td></tr>
<tr><td>Actual Lethality/Medical Damage:
0. No physical damage or very minor physical damage (e.g., surface scratches).
1. Minor physical damage (e.g., lethargic speech; first-degree burns; mild bleeding; sprains).
2. Moderate physical damage; medical attention needed (e.g., conscious but sleepy, somewhat responsive; second-degree burns; bleeding of major vessel).
3. Moderately severe physical damage; medical hospitalization and likely intensive care required (e.g., comatose with reflexes intact; third-degree burns less than 20% of body; extensive blood loss but can recover; major fractures).</td><td>Enter Code

______</td><td>Enter Code

______</td><td colspan="2">Enter Code

______</td></tr>
</table>

4. Severe physical damage; *medical* hospitalization with intensive care required (e.g., comatose without reflexes; third-degree burns over 20% of body; extensive blood loss with unstable vital signs; major damage to a vital area).<br>5. Death			
**Potential Lethality: Only Answer if Actual Lethality=0**<br>Likely lethality of actual attempt if no medical damage (the following examples, while having no actual medical damage, had potential for very serious lethality: put gun in mouth and pulled the trigger but gun fails to fire so no medical damage; laying on train tracks with oncoming train but pulled away before run over).<br><br>0 = Behavior not likely to result in injury<br>1 = Behavior likely to result in injury but not likely to cause death<br>2 = Behavior likely to result in death despite available medical care	*Enter Code*<br><br>______	*Enter Code*<br><br>______	*Enter Code*<br><br>______

© 2008 Research Foundation for Mental Hygiene, Inc. C-SSRS—Baseline (Version 1/14/09)

COLUMBIA-SUICIDE SEVERITY RATING SCALE – *SINCE LAST VISIT*

COLUMBIA-SUICIDE SEVERITY RATING SCALE (C-SSRS)

Since Last Visit

Version 1/14/09

Posner, K.; Brent, D.; Lucas, C.; Gould, M.; Stanley, B.; Brown, G.; Fisher, P.; Zelazny, J.; Burke, A.; Oquendo, M.; Mann, J.

Disclaimer:

This scale is intended to be used by individuals who have received training in its administration. The questions contained in the Columbia-Suicide Severity Rating Scale are suggested probes. Ultimately, the determination of the presence of suicidal ideation or behavior depends on the judgment of the individual administering the scale.

*Definitions of behavioral suicidal events in this scale are based on those used in **The Columbia Suicide History Form**, developed by John Mann, MD and Maria Oquendo, MD, Conte Center for the Neuroscience of Mental Disorders (CCNMD), New York State Psychiatric Institute, 1051 Riverside Drive, New York, NY, 10032. (Oquendo M. A., Halberstam B. & Mann J. J., Risk factors for suicidal behavior: utility and limitations of research instruments. In M.B. First [Ed.]* Standardized Evaluation in Clinical Practice*, pp. 103 -130, 2003.)*

For reprints of the C-SSRS contact Kelly Posner, Ph.D., New York State Psychiatric Institute, 1051 Riverside Drive, New York, New York, 10032; inquiries and training requirements contact posnerk@nyspi.columbia.edu

© 2008 The Research Foundation for Mental Hygiene, Inc.

SUICIDAL IDEATION	
Ask questions 1 and 2. If both are negative, proceed to "Suicidal Behavior" section. If the answer to question 2 is "yes", ask questions 3, 4 and 5. If the answer to question 1 and/or 2 is "yes", complete "Intensity of Ideation" section below.	**Since Last Visit**
**1. Wish to be Dead**<br>Subject endorses thoughts about a wish to be dead or not alive anymore, or wish to fall asleep and not wake up.<br>***Have you wished you were dead or wished you could go to sleep and not wake up?***<br>If yes, describe:	**Yes** ☐ **No** ☐
**2. Non-Specific Active Suicidal Thoughts**<br>General, non-specific thoughts of wanting to end one's life/commit suicide (e.g., *"I've thought about killing myself"*) without thoughts of ways to kill oneself/associated methods, intent, or plan during the assessment period.<br>***Have you actually had any thoughts of killing yourself?***<br>If yes, describe:	**Yes** ☐ **No** ☐
**3. Active Suicidal Ideation with Any Methods (Not Plan) without Intent to Act**<br>Subject endorses thoughts of suicide and has thought of at least one method during the assessment period. This is different than a specific plan with time, place or method details worked out (e.g., thought of method to kill self but not a specific plan). Includes person who would say, *"I thought about taking an overdose but I never made a specific plan as to when, where or how I would actually do it…and I would never go through with it."*<br>***Have you been thinking about how you might do this?***<br>If yes, describe:	**Yes** ☐ **No** ☐
**4. Active Suicidal Ideation with Some Intent to Act, without Specific Plan**<br>Active suicidal thoughts of killing oneself and subject reports having <u>some intent to act on such thoughts</u>, as opposed to *"I have the thoughts but I definitely will not do anything about them."*<br>***Have you had these thoughts and had some intention of acting on them?***<br>If yes, describe:	**Yes** ☐ **No** ☐
**5. Active Suicidal Ideation with Specific Plan and Intent**<br>Thoughts of killing oneself with details of plan fully or partially worked out and subject has some intent to carry it out.<br>***Have you started to work out or worked out the details of how to kill yourself? Do you intend to carry out this plan?***<br>If yes, describe:	**Yes** ☐ **No** ☐
INTENSITY OF IDEATION	
The following features should be rated with respect to the most severe type of ideation (i.e., 1-5 from above, with 1 being the least severe and 5 being the most severe).<br>***Most Severe Ideation:*** ____ ________________<br>***Type # (1-5)*** ***Description of Ideation***	Most Severe
**Frequency**<br>***How many times have you had these thoughts?***<br>(1) Less than once a week (2) Once a week (3) 2-5 times in week (4) Daily or almost daily (5) Many times each day	____
**Duration**<br>***When you have the thoughts, how long do they last?***<br>(1) Fleeting - few seconds or minutes<br>(2) Less than 1 hour/some of the time<br>(3) 1-4 hours/a lot of time<br>(4) 4-8 hours/most of day<br>(5) More than 8 hours/persistent or continuous	____
**Controllability**<br>***Could/can you stop thinking about killing yourself or wanting to die if you want to?***<br>(1) Easily able to control thoughts<br>(2) Can control thoughts with little difficulty<br>(3) Can control thoughts with some difficulty<br>(4) Can control thoughts with a lot of difficulty<br>(5) Unable to control thoughts<br>(0) Does not attempt to control thoughts	____

**Deterrents**<br>***Are there things - anyone or anything (e.g., family, religion, pain of death) - that stopped you from wanting to die or acting on thoughts of committing suicide?***<br>(1) Deterrents definitely stopped you from attempting suicide<br>(2) Deterrents probably stopped you<br>(3) Uncertain that deterrents stopped you<br>(4) Deterrents most likely did not stop you<br>(5) Deterrents definitely did not stop you<br>(0) Does not apply	____
**Reasons for Ideation**<br>***What sort of reasons did you have for thinking about wanting to die or killing yourself? Was it to end the pain or stop the way you were feeling (in other words you couldn't go on living with this pain or how you were feeling) or was it to get attention, revenge or a reaction from others? Or both?***<br>(1) Completely to get attention, revenge or a reaction from others<br>(2) Mostly to get attention, revenge or a reaction from others<br>(3) Equally to get attention, revenge or a reaction from others and to end/stop the pain<br>(4) Mostly to end or stop the pain (you couldn't go on living with the pain or how you were feeling)<br>(5) Completely to end or stop the pain (you couldn't go on living with the pain or how you were feeling)<br>(0) Does not apply	____

© 2008 Research Foundation for Mental Hygiene, Inc. C-SSRS—Since Last Visit (Version 1/14/09)

SUICIDAL BEHAVIOR<br>*(Check all that apply, so long as these are separate events; must ask about all types)*	**Since Last Visit**
**Actual Attempt:**<br>A potentially self-injurious act committed with at least some wish to die, *as a result of act.* Behavior was in part thought of as method to kill oneself. Intent does not have to be 100%. If there is ***any*** intent/desire to die associated with the act, then it can be considered an actual suicide attempt. ***There does not have to be any injury or harm***, just the potential for injury or harm. If person pulls trigger while gun is in mouth but gun is broken so no injury results, this is considered an attempt.<br>Inferring Intent: Even if an individual denies intent/wish to die, it may be inferred clinically from the behavior or circumstances. For example, a highly lethal act that is clearly not an accident so no other intent but suicide can be inferred (e.g., gunshot to head, jumping from window of a high floor/story). Also, if someone denies intent to die, but they thought that what they did could be lethal, intent may be inferred.<br>***Have you made a suicide attempt?***<br>***Have you done anything to harm yourself?***<br>***Have you done anything dangerous where you could have died?***<br>***What did you do?***<br>***Did you______ as a way to end your life?***<br>***Did you want to die (even a little) when you_____?***<br>***Were you trying to end your life when you _____?***<br>***Or did you think it was possible you could have died from_____?***<br>***Or did you do it purely for other reasons / without ANY intention of killing yourself (like to relieve stress, feel better, get sympathy, or get something else to happen)?*** (Self-Injurious Behavior without suicidal intent)<br>If yes, describe:	**Yes** **No**<br>☐ ☐<br><br>Total # of Attempts<br>______
**Has subject engaged in Non-Suicidal Self-Injurious Behavior?**	**Yes** **No**<br>☐ ☐
**Interrupted Attempt:**<br>When the person is interrupted (by an outside circumstance) from starting the potentially self-injurious act *(if not for that, actual attempt would have occurred).*<br>Overdose: Person has pills in hand but is stopped from ingesting. Once they ingest any pills, this becomes an attempt rather than an interrupted attempt. Shooting: Person has gun pointed toward self, gun is taken away by someone else, or is somehow prevented from pulling trigger. Once they pull the trigger, even if the gun fails to fire, it is an attempt. Jumping: Person is poised to jump, is grabbed and taken down from ledge. Hanging: Person has noose around neck but has not yet started to hang - is stopped from doing so.<br>***Has there been a time when you started to do something to end your life but someone or something stopped you before you actually did anything?***<br>If yes, describe:	**Yes** **No**<br>☐ ☐<br><br>Total # of interrupted<br>______
**Aborted Attempt:**<br>When person begins to take steps toward making a suicide attempt, but stops themselves before they actually have engaged in any self-destructive behavior. Examples are similar to interrupted attempts, except that the individual stops him/herself, instead of being stopped by something else.<br>***Has there been a time when you started to do something to try to end your life but you stopped yourself before you actually did anything?***<br>If yes, describe:	**Yes** **No**<br>☐ ☐<br><br>Total # of aborted

	______
**Preparatory Acts or Behavior:**<br>Acts or preparation towards imminently making a suicide attempt. This can include anything beyond a verbalization or thought, such as assembling a specific method (e.g., buying pills, purchasing a gun) or preparing for one's death by suicide (e.g., giving things away, writing a suicide note).<br>***Have you taken any steps towards making a suicide attempt or preparing to kill yourself (such as collecting pills, getting a gun, giving valuables away or writing a suicide note)?***<br>If yes, describe:	**Yes** **No**<br>☐ ☐
**Suicidal Behavior:**<br>Suicidal behavior was present during the assessment period?	**Yes** **No**<br>☐ ☐
**Suicide:**	**Yes** **No**<br>☐ ☐
Answer for Actual Attempts Only	Most Lethal Attempt Date:
**Actual Lethality/Medical Damage:**<br>0. No physical damage or very minor physical damage (e.g., surface scratches).<br>1. Minor physical damage (e.g., lethargic speech; first-degree burns; mild bleeding; sprains).<br>2. Moderate physical damage; medical attention needed (e.g., conscious but sleepy, somewhat responsive; second-degree burns; bleeding of major vessel).<br>3. Moderately severe physical damage; *medical* hospitalization and likely intensive care required (e.g., comatose with reflexes intact; third-degree burns less than 20% of body; extensive blood loss but can recover; major fractures).<br>4. Severe physical damage; *medical* hospitalization with intensive care required (e.g., comatose without reflexes; third-degree burns over 20% of body; extensive blood loss with unstable vital signs; major damage to a vital area).<br>5. Death	*Enter Code*<br>______
**Potential Lethality: Only Answer if Actual Lethality=0**<br>Likely lethality of actual attempt if no medical damage (the following examples, while having no actual medical damage, had potential for very serious lethality: put gun in mouth and pulled the trigger but gun fails to fire so no medical damage; laying on train tracks with oncoming train but pulled away before run over).<br>0 = Behavior not likely to result in injury<br>1 = Behavior likely to result in injury but not likely to cause death<br>2 = Behavior likely to result in death despite available medical care	*Enter Code*<br>______

© 2008 Research Foundation for Mental Hygiene, Inc. C-SSRS—Since Last Visit (Version 1/14/09)

Gastrointestinal Symptoms Rating Scale (GSRS)

Symptom	Number of Symptom Days	Duration or Number of Episodes (if applicable); Sum: duration/episode score	Highest Level of Urgency (if applicable)	Severity; Sum severity score	Interference with Activities; Sum interference score	Total Sum of Scores by Symptoms (Across Domains)
Diarrhea	2	13 episodes; sum=5	Extremely urgent; sum=7	Severe; sum=8	Completely; sum=7	Sum=27
Bloating	2	>5 hours; sum=4	N/A	Moderate; sum=4	Moderate; sum=3	Sum=11
Abdominal Pain	1	2 hours; sum=2	N/A	Moderate; sum=3	Moderate; sum=2	Sum=7
Headache	1	2 hours; sum=2	N/A	Mild; sum=2	A little bit; sum=1	Sum=5
Tiredness	1	8 hours; sum=4	N/A	Moderate; sum=3	Moderate; sum=2	Sum=9
Nausea	1	1 hour; sum=2	N/A	Mild; sum=2	A little bit; sum=1	Sum=5
Total	8 Symptom Days	Duration/Episodes Category Sum=19	Urgency Category Sum=7	2 Severe; 3 moderate; 2 mild; 1 very mild. Severity Category Sum=22	1 Complete; 1 Very much, 3 moderate; 3 A little bit. Interference Category Sum=16	Total Sum=64

Screening Tool: Geriatric Depression Scale (GDS)

1. Are you basically satisfied with your life?	Yes	**No (1)**
2. Have you dropped many of your activities and interests?	**Yes (1)**	No
3. Do you feel that your life is empty?	**Yes (1)**	No
4. Do you often get bored?	**Yes (1)**	No
5. Are you in good spirits most of the time?	Yes	**No (1)**
6. Are you afraid that something bad is going to happen to you?	**Yes (1)**	No
7. Do you feel happy most of the time?	Yes	**No (1)**
8. Do you often feel helpless?	**Yes (1)**	No
9. Do you prefer to stay at home, rather than going out and doing new things?	**Yes (1)**	No
10. Do you feel you have more problems with memory than most?	**Yes (1)**	No
11. Do you think it is wonderful to be alive now?	Yes	**No (1)**
12. Do you feel pretty worthless the way you are now?	**Yes (1)**	No
13. Do you feel full of energy?	Yes	**No (1)**
14. Do you feel that your situation is hopeless?	**Yes (1)**	No
15. Do you think that most people are better off than you are?	**Yes (1)**	No

Sum all bolded answers (worth one point each) for a total score: __________

A score >5 points is suggestive of depression and should warrant follow-up intervention.

A score >10 points is almost always depression.

____ ____ ____ ____ ____ ____ ____ ____<br>Patient Name or Subject ID	____ ____ ____<br>Site ID	___ ___ - ___ ___ - ___ ___ ___ ___<br>(mm-dd-yyyy)<br>Assessment Date	____ ____ ____<br>Investigator's Initials

MDS UPDRS
Part I: Non-Motor Aspects of Experiences of Daily Living (nM-EDL)

Part 1A: Complex behaviors: [completed by rater]

Primary source of information:

☐ Patient ☐ Caregiver ☐ Patient and Caregiver in Equal Proportion

To be read to the patient: I am going to ask you six questions about behaviors that you may or may not experience. Some questions concern common problems and some concern uncommon ones. If you have a problem in one of the areas, please choose the best response that describes how you have felt MOST OF THE TIME during the PAST WEEK. If you are not bothered by a problem, you can simply respond NO. I am trying to be thorough, so I may ask questions that have nothing to do with you.

1.1 COGNITIVE IMPAIRMENT **SCORE**

Instructions to examiner: Consider all types of altered level of cognitive function including cognitive slowing, impaired reasoning, memory loss, deficits in attention and orientation. Rate their impact on activities of daily living as perceived by the patient and/or caregiver.

Instructions to patients [and caregiver]: Over the past week have you had problems remembering things, following conversations, paying attention, thinking clearly, or finding your way around the house or in town? [If yes, examiner asks patient or caregiver to elaborate and probes for information]

0:	Normal:	No cognitive impairment.
1:	Slight:	Impairment appreciated by patient or caregiver with no concrete interference with the patient's ability to carry out normal activities and social interactions.
2:	Mild:	Clinically evident cognitive dysfunction, but only minimal interference with the patient's ability to carry out normal activities and social interactions.
3:	Moderate:	Cognitive deficits interfere with but do not preclude the patient's ability to carry out normal activities and social interactions.
4:	Severe:	Cognitive dysfunction precludes the patient's ability to carry out normal activities and social interactions.

☐

	SCORE
**1.2 HALLUCINATIONS AND PSYCHOSIS**<br><br>Instructions to examiner: Consider both illusions (misinterpretations of real stimuli) and hallucinations (spontaneous false sensations). Consider all major sensory domains (visual, auditory, tactile, olfactory and gustatory). Determine presence of unformed (for example sense of presence or fleeting false impressions) as well as formed (fully developed and detailed) sensations. Rate the patients insight into hallucinations and identify delusions and psychotic thinking.<br><br>*Instructions to patients [and caregiver]: Over the past week have you seen, heard, smelled or felt things that were not really there?* [If yes, examiner asks patient or caregiver to elaborate and probes for information]<br><br>0: Normal: No hallucinations or psychotic behaviour.<br>1: Slight: Illusions or non-formed hallucinations, but patient recognizes them without loss of insight.<br>2: Mild: Formed hallucinations independent of environmental stimuli. No loss of insight.<br>3: Moderate: Formed hallucinations with loss of insight.<br>4: Severe: Patient has delusions or paranoia.	☐
**1.3 DEPRESSED MOOD**<br><br>Instructions to examiner: Consider low mood, sadness, hopelessness, feelings of emptiness or loss of enjoyment. Determine their presence and duration over the past week and rate their interference with the patient's ability to carry out daily routines and engage in social interactions.<br><br>*Instruction to the patient (and caregiver): Over the past week have you felt low, sad, hopeless or unable to enjoy things? If yes, was this feeling for longer than one day at a time? Did it make it difficult for you carry out your usual activities or to be with people?* If yes, examiner asks patient or caregiver to elaborate and probes for information]<br><br>0: Normal: No depressed mood.<br>1: Slight: Episodes of depressed mood that are not sustained for more than one day at a time. No interference with patient's ability to carry out normal activities and social interactions.<br>2: Mild: Depressed mood that is sustained over days, but without interference with normal activities and social interactions.<br>3: Moderate: Depressed mood that interferes with, but does not preclude, the patient's ability to carry out normal activities and social interactions.<br>4: Severe: Depressed mood precludes patient's ability to carry out normal activities and social interactions.	☐

SCORE

1.4 ANXIOUS MOOD

Instructions to examiner: Determine nervous, tense, worried or anxious feelings (including panic attacks) over the past week and rate their duration and interference with the patient's ability to carry out daily routines and engage in social interactions.

Instructions to patients [and caregiver]: Over the past week have you felt nervous, worried or tense? If yes, was this feeling for longer than one day at a time? Did it make it difficult for you to follow your usual activities or to be with other people? [If yes, examiner asks patient or caregiver to elaborate and probes for information.]

0: Normal: No anxious feelings.

1: Slight: Anxious feelings present but not sustained for more than one day at a time. No interference with patient's ability to carry out normal activities and social interactions.

2: Mild: Anxious feelings are sustained over more than one day at a time, but without interference with patient's ability to carry out normal activities and social interactions.

3: Moderate: Anxious feelings interfere with, but do not preclude, the patient's ability to carry out normal activities and social interactions.

4: Severe: Anxious feelings preclude patient's ability to carry out normal activities and social interactions.

☐

1.5 APATHY

Instructions to examiner: Consider level of spontaneous activity, assertiveness, motivation and initiative and rate the impact of reduced levels on performance of daily routines and social interactions. Here the examiner should attempt to distinguish between apathy and similar symptoms that are best explained by depression.

Instructions to patients (and caregiver): Over the past week, have you felt indifferent to doing activities or being with people? If yes, examiner asks patient or caregiver to elaborate and probes for information.]

0: Normal: No apathy.

1: Slight: Apathy appreciated by patient and/or caregiver, but no interference with daily activities and social interactions.

2: Mild: Apathy interferes with isolated activities and social interactions.

3: Moderate: Apathy interferes with most activities and social interactions.

4: Severe: Passive and withdrawn, complete loss of initiative.

☐

1.6 FEATURES OF DOPAMINE DYSREGULATION SYNDROME	SCORE
Instructions to examiner: Consider involvement in a variety of activities including atypical or excessive gambling (e.g. casinos or lottery tickets), atypical or excessive sexual drive or interests (e.g., unusual interest in pornography, masturbation, sexual demands on partner), other repetitive activities (e.g. hobbies, dismantling objects, sorting or organizing), or taking extra non-prescribed medication for non-physical reasons (i.e., addictive behavior). Rate the impact of such abnormal activities/behaviors on the patient's personal life and on his family and social relations (including need to borrow money or other financial difficulties like withdrawal of credit cards, major family conflicts, lost time from work, or missed meals or sleep because of the activity).<br><br>*Instructions to patients [and caregiver]: Over the past week, have you had unusually strong urges that are hard to control? Do you feel driven to do or think about something and find it hard to stop?* [Give patient examples such as gambling, cleaning, using the computer, taking extra medicine, obsessing about food or sex, all depending on the patients.<br><br>0: Normal: No problems present.<br>1: Slight: Problems are present but usually do not cause any difficulties for the patient or family/caregiver.<br>2: Mild: Problems are present and usually cause a few difficulties in the patient's personal and family life.<br>3: Moderate: Problems are present and usually cause a lot of difficulties in the patient's personal and family life.<br>4: Severe: Problems are present and preclude the patient's ability to carry out normal activities or social interactions or to maintain previous standards in personal and family life.	☐

The remaining questions in Part I (Non-motor Experiences of Daily Living) [Sleep, Daytime Sleepiness, Pain and Other Sensation, Urinary Problems, Constipation Problems, Lightheadedness on Standing, and Fatigue] are in the **Patient Questionnaire** along with all questions in Part II [Motor Experiences of Daily Living].

Patient Questionnaire:

Instructions:

This questionnaire will ask you about your experiences of daily living.

There are 20 questions. We are trying to be thorough, and some of these questions may therefore not apply to you now or ever. If you do not have the problem, simply mark 0 for NO.

Please read each one carefully and read all answers before selecting the one that best applies to you.

We are interested in your average or usual function over the past week including today. Some patients can do things better at one time of the day than at others. However, only one answer is allowed for each question, so please mark the answer that best describes what you can do most of the time.

You may have other medical conditions besides Parkinson's disease. Do not worry about separating Parkinson's disease from other conditions. Just answer the question with your best response.

Use only 0, 1, 2, 3, 4 for answers, nothing else. Do not leave any blanks.

Your doctor or nurse can review the questions with you, but this questionnaire is for patients to complete, either alone or with their caregivers.

Who is filling out this questionnaire (check the best answer):

☐ Patient ☐ Caregiver ☐ Patient and Caregiver in Equal Proportion

Part I: Non-Motor Aspects of Experiences of Daily Living (nM-EDL)	
**1.7 SLEEP PROBLEMS**<br><br>Over the past week, have you had trouble going to sleep at night or staying asleep through the night? Consider how rested you felt after waking up in the morning.<br><br>0: Normal: No problems.<br>1: Slight: Sleep problems are present but usually do not cause trouble getting a full night of sleep.<br>2: Mild: Sleep problems usually cause some difficulties getting a full night of sleep.<br>3: Moderate: Sleep problems cause a lot of difficulties getting a full night of sleep, but I still usually sleep for more than half the night.<br>4: Severe: I usually do not sleep for most of the night.	**SCORE**<br><br>☐
**1.8 DAYTIME SLEEPINESS**<br><br>Over the past week, have you had trouble staying awake during the daytime?<br><br>0: Normal: No daytime sleepiness.<br>1: Slight: Daytime sleepiness occurs but I can resist and I stay awake.<br>2: Mild: Sometimes I fall asleep when alone and relaxing. For example, while reading or watching TV.<br>3: Moderate: I sometimes fall asleep when I should not. For example, while eating or talking with other people.<br>4: Severe: I often fall asleep when I should not. For example, while eating or talking with other people.	☐

	SCORE
**1.9 PAIN AND OTHER SENSATIONS**<br><br>Over the past week, have you had uncomfortable feelings in your body like pain, aches tingling or cramps?<br><br>0: Normal: No uncomfortable feelings.<br><br>1: Slight: I have these feelings. However, I can do things and be with other people without difficulty.<br><br>2: Mild: These feelings cause some problems when I do things or am with other people.<br><br>3: Moderate: These feelings cause a lot of problems, but they do not stop me from doing things or being with other people.<br><br>4: Severe: These feelings stop me from doing things or being with other people.	☐
**1.10 URINARY PROBLEMS**<br><br>Over the past week, have you had trouble with urine control? For example, an urgent need to urinate, a need to urinate too often, or urine accidents?<br><br>0: Normal: No urine control problems.<br><br>1: Slight: I need to urinate often or urgently. However, these problems do not cause difficulties with my daily activities.<br><br>2: Mild: Urine problems cause some difficulties with my daily activities. However, I do not have urine accidents.<br><br>3: Moderate: Urine problems cause a lot of difficulties with my daily activities, including urine accidents.<br><br>4: Severe: I cannot control my urine and use a protective garment or have a bladder tube.	☐

SCORE

1.11 CONSTIPATION PROBLEMS

Over the past week have you had constipation troubles that cause you difficulty moving your bowels?

0: Normal: No constipation.

1: Slight: I have been constipated. I use extra effort to move my bowels. However, this problem does not disturb my activities or my being comfortable.

2: Mild: Constipation causes me to have some troubles doing things or being comfortable.

3: Moderate: Constipation causes me to have a lot of trouble doing things or being comfortable. However, it does not stop me from doing anything.

4: Severe: I usually need physical help from someone else to empty my bowels.

☐

1.12 LIGHT HEADEDNESS ON STANDING

Over the past week, have you felt faint, dizzy or foggy when you stand up after sitting or lying down?

0: Normal: No dizzy or foggy feelings.

1: Slight: Dizzy or foggy feelings occur. However, they do not cause me troubles doing things.

2: Mild: Dizzy or foggy feelings cause me to hold on to something, but I do not need to sit or lie back down.

3: Moderate: Dizzy or foggy feelings cause me to sit or lie down to avoid fainting or falling.

4: Severe: Dizzy or foggy feelings cause me to fall or faint.

☐

	SCORE
**1.13 FATIGUE**<br><br>Over the past week, have you usually felt fatigued? This feeling is not part of being sleepy or sad<br><br>0: Normal: No fatigue.<br><br>1: Slight: Fatigue occurs. However it does not cause me troubles doing things or being with people.<br><br>2: Mild: Fatigue causes me some troubles doing things or being with people.<br><br>3: Moderate: Fatigue causes me a lot of troubles doing things or being with people. However, it does not stop me from doing anything.<br><br>4: Severe: Fatigue stops me from doing things or being with people.	☐
**Part II: Motor Aspects of Experiences of Daily Living (M-EDL)**	
**2.1 SPEECH**<br><br>Over the past week, have you had problems with your speech?<br><br>0: Normal: Not at all (no problems).<br><br>1: Slight: My speech is soft, slurred or uneven, but it does not cause others to ask me to repeat myself.<br><br>2: Mild: My speech causes people to ask me to occasionally repeat myself, but not everyday.<br><br>3: Moderate: My speech is unclear enough that others ask me to repeat myself every day even though most of my speech is understood.<br><br>4: Severe: Most or all of my speech cannot be understood.	☐

SCORE

2.2 SALIVA & DROOLING

Over the past week, have you usually had too much saliva during when you are awake or when you sleep?

0:	Normal:	Not at all (no problems).
1:	Slight:	I have too much saliva, but do not drool.
2:	Mild:	I have some drooling during sleep, but none when I am awake.
3:	Moderate:	I have some drooling when I am awake, but I usually do not need tissues or a handkerchief.
4:	Severe:	I have so much drooling that I regularly need to use tissues or a handkerchief to protect my clothes.

☐

2.3 CHEWING AND SWALLOWING

Over the past week, have you usually had problems swallowing pills or eating meals? Do you need your pills cut or crushed or your meals to be made soft, chopped or blended to avoid choking?

0:	Normal:	No problems.
1:	Slight:	I am aware of slowness in my chewing or increased effort at swallowing, but I do not choke or need to have my food specially prepared.
2:	Mild:	I need to have my pills cut or my food specially prepared because of chewing or swallowing problems, but I have not choked over the past week.
3:	Moderate.	I choked at least once in the past week.
4:	Severe:	Because of chewing and swallowing problems, I need a feeding tube.

☐

	SCORE
**2.4 EATING TASKS**<br><br>Over the past week, have you usually had troubles handling your food and using eating utensils? For example, do you have trouble handling finger foods or using forks, knifes, spoons, chopsticks?<br><br>0: Normal: Not at all (No problems).<br>1: Slight: I am slow, but I do not need any help handling my food and have not had food spills while eating.<br>2: Mild: I am slow with my eating and have occasional food spills. I may need help with a few tasks such as cutting meat.<br>3: Moderate: I need help with many eating tasks but can manage some alone.<br>4: Severe: I need help for most or all eating tasks.	☐
**2.5 DRESSING**<br><br>Over the past week, have you usually had problems dressing? For example, are you slow or do you need help with buttoning, using zippers, putting on or taking off your clothes or jewelry?<br><br>0: Normal: Not at all (no problems).<br>1: Slight: I am slow but I do not need help.<br>2: Mild: I am slow and need help for a few dressing tasks (buttons, bracelets).<br>3: Moderate: I need help for many dressing tasks.<br>4: Severe: I need help for most or all dressing tasks.	☐

	SCORE
**2.6 HYGIENE**<br><br>Over the past week, have you usually been slow or do you need help with washing, bathing, shaving, brushing teeth, combing your hair or with other personal hygiene?<br><br>0: Normal: Not at all (no problems).<br>1: Slight: I am slow but I do not need any help.<br>2: Mild: I need someone else to help me with some hygiene tasks.<br>3: Moderate: I need help for many hygiene tasks.<br>4: Severe: I need help for most or all of my hygiene tasks.	☐
**2.7 HANDWRITING**<br><br>Over the past week, have people usually had trouble reading your handwriting?<br><br>0: Normal: Not at all (no problems).<br>1: Slight: My writing is slow, clumsy or uneven, but all words are clear.<br>2: Mild: Some words are unclear and difficult to read.<br>3: Moderate: Many words are unclear and difficult to read.<br>4: Severe: Most or all words cannot be read.	☐
**2.8 DOING HOBBIES AND OTHER ACTIVITIES**<br><br>Over the past week, have you usually had trouble doing your hobbies or other things that you like to do?<br><br>0: Normal: Not at all (no problems).<br>1: Slight: I am a bit slow but do these activities easily.<br>2: Mild: I have some difficulty doing these activities.<br>3: Moderate: I have major problems doing these activities, but still do most.<br>4: Severe: I am unable to do most or all of these activities.	☐

	SCORE
**2.9 TURNING IN BED**<br><br>Over the past week, do you usually have trouble turning over in bed?<br><br>0: Normal: Not at all (no problems).<br>1: Slight: I have a bit of trouble turning, but I do not need any help.<br>2: Mild I have a lot of trouble turning and need occasional help from someone else.<br>3: Moderate: To turn over I often need help from someone else.<br>4: Severe: I am unable to turn over without help from someone else.	☐
**2.10 TREMOR**<br><br>Over the past week, have you usually had shaking or tremor?<br><br>0: Normal: Not at all. I have no shaking or tremor.<br>1: Slight: Shaking or tremor occurs but does not cause problems with any activities.<br>2: Mild: Shaking or tremor causes problems with only a few activities.<br>3: Moderate: Shaking or tremor causes problems with many of my daily activities.<br>4: Severe: Shaking or tremor causes problems with most or all activities.	☐
**2.11 GETTING OUT OF BED, A CAR, OR A DEEP CHAIR**<br><br>Over the past week, have you usually had trouble getting out of bed, a car seat, or a deep chair?<br><br>0: Normal: Not at all (no problems).<br>1: Slight: I am slow or awkward, but I usually can do it on my first try.<br>2: Mild: I need more than one try to get up or need occasional help.<br>3: Moderate: I sometimes need help to get up, but most times I can still do it on my own.<br>4: Severe: I need help most or all of the time.	☐

	SCORE
**2.12 WALKING AND BALANCE**<br><br>Over the past week, have you usually had problems with balance and walking?<br><br>0: Normal: Not at all (no problems).<br><br>1: Slight: I am slightly slow or may drag a leg. I never use a walking aid.<br><br>2: Mild: I occasionally use a walking aid, but I do not need any help from another person.<br><br>3: Moderate: I usually use a walking aid (cane, walker) to walk safely without falling. However, I do not usually need the support of another person.<br><br>4: Severe: I usually use the support of another persons to walk safely without falling.	☐
**2.13 FREEZING**<br><br>Over the past week, on your usual day when walking, do you suddenly stop or freeze as if your feet are stuck to the floor.<br><br>0: Normal: Not at all (no problems).<br><br>1: Slight: I briefly freeze but I can easily start walking again. I do not need help from someone else or a walking aid (cane or walker) because of freezing.<br><br>2: Mild: I freeze and have trouble starting to walk again, but I do not need someone's help or a walking aid (cane or walker) because of freezing.<br><br>3: Moderate: When I freeze I have a lot of trouble starting to walk again and, because of freezing, I sometimes need to use a walking aid or need someone else's help.<br><br>4: Severe: Because of freezing, most or all of the time, I need to use a walking aid or someone's help.	☐
This completes the questionnaire. We may have asked about problems you do not even have, and may have mentioned problems that you may never develop at all. Not all patients develop all these problems, but because they can occur, it is important to ask all the questions to every patient. Thank you for your time and attention in completing this questionnaire.	
