# Supplementary material for: Nilotinib Effects on Safety, Tolerability, and Potential Biomarkers in Parkinson Disease: A Phase 2 Randomized Clinical Trial
Source: JAMA Neurol. 2019 Dec 16;77(3):309–17. doi: 10.1001/jamaneurol.2019.4200 (PMC6990742; doi:10.1001/jamaneurol.2019.4200)
Supplement: Supplement 2. — eMethods. Detailed Methods eReferences. eFigure 1. Data Represent the Effects of 1-Year Nilotinib Treatment on Abl Activation via Tyrosine Phosphorylation and Plasma Dopamine Metabolites Compared to Placebo eFigure 2. Clinical Effects of Nilotinib in PD Using the Movement Disorders Society—Unified Parkinson Disease Rating Scale (MDS-UPDRS) and Parkinson’s Disease Questionnaire (PDQ)-39 eTable 1. Summary of EKG Values for All Participants Throughout All Study Visits Showing No QTc Prolongation in the Placebo Group eTable 2. Summary of EKG Values for All Participants Throughout All Study Visits Showing No QTc Prolongation in the 150mg Nilotinib Group eTable 3. Summary of EKG Values for All Participants Throughout All Study Visits Showing No QTc Prolongation in the 300mg Nilotinib Group eTable 4. Levels of Exploratory CSF and Plasma Biomarkers eTable 5. Pairwise Comparison of Clinical Endpoints Using Mean Differences Based on Raw Value at 6, 12 and 15 Months Compared to Baseline eTable 6. Average Values of Each Clinical Endpoint in Placebo, 150mg Nilotinib and 300mg Nilotinib Groups eTable 7. Statistical Analysis Showing in Mixed Effects Model and Test P Values and ANOVA Test for Group Differences eTable 8. Demographics Showing Participants Who Received Dopamine Agonists (DA) Alone Versus Participants Who Received DA+ Levodopa eTable 9. Concentration of Nilotinib in CSF and Plasma [file jamaneurol-77-309-s002.pdf]

Supplementary Online Content

Pagan FL, Hebron ML, Wilmarth B, et al. Nilotinib effects on safety, tolerability, and potential biomarkers in Parkinson disease: a phase 2 randomized clinical trial. *JAMA Neurol*. Published online December 16, 2019. doi:10.1001/jamaneurol.2019.4200

eMethods. Detailed Methods

eReferences.

eFigure 1. Data Represent the Effects of 1-Year Nilotinib Treatment on Abl Activation via Tyrosine Phosphorylation and Plasma Dopamine Metabolites Compared to Placebo

eFigure 2. Clinical Effects of Nilotinib in PD Using the Movement Disorders Society—Unified Parkinson Disease Rating Scale (MDS-UPDRS) and Parkinson's Disease Questionnaire (PDQ)-39

eTable 1. Summary of EKG Values for All Participants Throughout All Study Visits Showing No QTc Prolongation in the Placebo Group

eTable 2. Summary of EKG Values for All Participants Throughout All Study Visits Showing No QTc Prolongation in the 150mg Nilotinib Group

eTable 3. Summary of EKG Values for All Participants Throughout All Study Visits Showing No QTc Prolongation in the 300mg Nilotinib Group

eTable 4. Levels of Exploratory CSF and Plasma Biomarkers

eTable 5. Pairwise Comparison of Clinical Endpoints Using Mean Differences Based on Raw Value at 6, 12 and 15 Months Compared to Baseline

eTable 6. Average Values of Each Clinical Endpoint in Placebo, 150mg Nilotinib and 300mg Nilotinib Groups

eTable 7. Statistical Analysis Showing in Mixed Effects Model and Test P Values and ANOVA Test for Group Differences

eTable 8. Demographics Showing Participants Who Received Dopamine Agonists (DA) Alone Versus Participants Who Received DA+ Levodopa

eTable 9. Concentration of Nilotinib in CSF and Plasma.

This supplementary material has been provided by the authors to give readers additional information about their work.

eMethods. Detailed Methods

Participants. All participants were confirmed to have PD according to the UK Brain Bank diagnostic criteria, with Hoehn and Yahr (H&Y) stage 2.5-3, Movement Disorders Society-Unified Parkinson's Disease Rating Scale (MDS-UPDRS)-III motor score 20-40 and Montreal Cognitive Assessment (MoCA) score ≥22. All participants were optimized on PD medications, including≤ 800mg/day levodopa and/or dopamine agonists approximately 1-2 months before consenting in writing and screening. Baseline visits were scheduled 2-4 weeks after screening and results from all screening procedures were reviewed and all inclusion/exclusion criteria were met prior to baseline assessments. Male and female participants of any race and/or nationality aged 40-90 years were allowed to enroll with no restriction to any geographical boundaries as long as they could comply with study procedures. PD symptoms were stabilized on optimal levodopa with and without therapeutic doses of amantadine and dopamine agonists, including Bromocriptine (Parlodel), Pramipexole (Mirapex) and Ropinirole (Requip) and Rotigotine (Neupro). Monoamine oxidase (MAO)-B inhibitors were excluded at least 6 weeks prior to screening and enrollment in order to allow pk/pd studies of CSF dopamine metabolism. Other medications such as Acetylcholinesterase inhibitors (AChEI), including Galantamine (Razadyne), Rivastagmine (Excelon) and Donepezil (Aricept) were also used. Therapeutic doses of Selective Serotonin Reuptake Inhibitors (SSRIs), including Citalopram (Celexa, Cipramil), Escitalopram (Lexapro, Cipralex), Fluoxetine (Prozac), Paroxetine (Paxil) and Sertraline (Zoloft, Lustral) and Serotonin-Norepinephrine Reuptake Inhibitors (SNRIs), Duloxetine (Cymbalta) and Venlafaxine (Effexor) were allowed. Participants were also willing to undergo lumbar puncture (LP) at baseline and 12 months. Eligible participants provided a written informed consent and complied with study procedures.

Study Design and Objectives. This study is part of a phase II, 3-phase clinical trial that included (phase 1) an open label random single dose (RSD) experiment to perform a physiologically-based population pharmacokinetics/pharmacodynamics (popPK/PD) study that was already published[1]. In the RSD study, a total of 100 participants were screened and 75 individuals were randomized 1:1:1:1:1 into 5 groups receiving a single dose of 150, 200, 300 and 400 mg nilotinib versus placebo (n=15 per group)[1]. Following RSD, the same participants were again randomized 1:1:1 into 3 groups (n=25) and received placebo, 150mg or 300mg nilotinib (phase 2) once daily for one year followed by a 3-month washout period. Here we report the results of a randomized, double-blind, placebo-controlled study to evaluate the safety and clinical effects of nilotinib (phase 2) in participants with PD. Phase 3 is an open label extension for additional 12 months for all eligible participants who completed this phase 2 and 3-month wash-out period.

The primary objective of this study was to evaluate the safety, tolerability and pharmacokinetics of nilotinib in individuals with PD. Safety was measured using the occurrence of adverse events (AEs) and serious adverse events (SAEs) deemed to be possibly, probably, or definitely related to the study drug. AEs were defined as QTc prolongation, myelosuppression, hepatotoxicity and pancreatitis. These AEs were tracked over the course of the trial and reviewed by the Data and Safety Monitoring Board (DSMB) at scheduled meetings and in real time and on a case-by-case basis. Tolerability for a given participant was defined as the ability of participants to remain on treatment. Overall tolerability of the drug was defined as an acceptable number of up to 25% discontinuations. The primary objective of this study also included measurement of nilotinib in the CSF. We previously demonstrated that nilotinib enters the brain in a dose-independent manner[1], and here we report the pharmacodynamics (pd) of nilotinib (secondary objectives) in the plasma and CSF of all participants after 12 months. An exploratory objective

also included clinical assessments of motor and non-motor functions using MDS-UPDRS, Parkinson's Disease Questionnaire (PDQ)-39, MoCA and Timed-Up and Go (TUG).

Standard Protocol Approvals, Registrations, and Patient Consents. This is a single center study that was conducted by the Translational Neurotherapeutics Program (TNP) at Georgetown University Medical Center (GUMC) Clinical Research Unit (CRU) of Georgetown-Howard Universities Center for Clinical and Translational Science (GHUCCTS). GHUCCTS encompasses MedStar Health Research Institute (10 hospitals), including Georgetown University Hospital (GUH), Howard University, the Washington DC VA Medical Center (with its hospital and five clinics) and the Oak Ridge National Laboratory. Participants were enrolled from the movement disorder center at MedStar GUH and other affiliate centers as well other interested individuals who were eligible to enroll from the continental USA and Europe. This study was conducted in accordance with Good Clinical Practice guidelines and was approved by the Institutional Review Board (IRB# 2016-0380) at GUMC as well as GHUCCTS scientific review board. The study was conducted under FDA Investigational New Drug (IND) # 123183, and registered in ClinicalTrials.gov (NCT02954978). An external independent DSMB that includes a movement disorders neurologist, a biostatistician, a cardiologist and a clinical pharmacologist, as well as an independent study monitor, were appointed to monitor study safety and progress.

Plasma and CSF collection. We previously showed that nilotinib was detected in the CSF for 4 hours after administration[2]. Blood draw (15 ml) and lumbar puncture (LP) ~15 ml CSF were performed on all patients approximately 2 hours after the last levodopa dose and at 1, 2, 3 or 4 hours after nilotinib administration. Plasma was isolated immediately after blood draw and aliquoted and stored at –80°C. CSF was aliquoted and stored at –80°C. Freeze/thaw cycles were avoided. To avoid CSF contamination with blood, the first 1 mL of CSF collection was discarded

and all samples were centrifuged at 1000 g for 15 minutes. Samples that contained>25 ng/ml hemoglobin were eliminated and were not tested for biomarkers. The hemoglobin levels in CSF samples were measured using human hemoglobin Enzyme-Linked Immunosorbent Assay (ELISA) Quantitation (Cat # E80-136) Kit (Bethyl Lab Inc, Montgomery, TX, USA) according to the manufacturer's instructions.

Abl ELISA. Phospho-Abl (pan-tyrosine) ELISA detects levels of Bcr-Abl and Abl proteins when phosphorylated at tyrosine residues and phospho-Abl (Tyr 412) ELISA detects levels of Bcr-Abl and c-Abl proteins when phosphorylated at tyrosine 412 residue. PathScan® phospho-Abl solid phase sandwich ELISA was performed on CSF and plasma. 100 μL CSF or plasma was added to Abl polyclonal rabbit coated micro-wells for 2hrs at 37^0C. Bcr-Abl and Abl proteins are captured by the coated antibody. After washing, a pan-tyrosine phosphorylated Abl or tyrosine 412 phosphorylated detection antibodies were added to each well to detect captured tyrosine-phosphorylated Bcr-Abl and Abl proteins. Samples were incubated with detection antibody for 1 hour at 37^0C. After washing, anti-mouse IgG, horse radish peroxidase (HRP)-linked antibody was added and incubated for 10 min at 37^0C to recognize the bound detection antibody. HRP substrate, TMB was added to develop color. The magnitude of the absorbance for developed color is proportional to the quantity of tyrosine-phosphorylated Bcr-Abl and Abl proteins in the samples.

Tau ELISA. Milliplex ELISA exploits Xmap technology that uses magnetic microspheres that are internally coded with two fluorescent dyes. Through precise combinations of these two dyes, multiple proteins are simultaneously measured within a sample. Each of these spheres is coated with a specific capture antibody. CSF and plasma samples were analyzed in parallel using the same reagents. A total of 25 μL of soluble protein was incubated overnight at 4 C with 25 μL

of a mixed-bead solution containing total tau, and hyper-phosphorylated tau (p-tau 181) 25 µL of detection antibody solution (Cat. #HNABTMAG-68 K, Millipore). After washing, 25 µL of streptavidin-phycoerythrin was added to each well containing suspended beads and incubated at RT for 30 minutes. Samples were then washed and suspended in 100 µL of sheath fluid. Next, samples were run on MAGPIX with Xponent software. The median fluorescent intensity data were analyzed using a five-parameter logistic or spline curve-fitting method for calculating analyte concentrations in samples.

Mass spectrometry to evaluate nilotinib pharmacokinetics, Plasma and CSF samples (20µl) were thawed initially on ice at RT and transferred to Eppendorf tubes containing 100µl of water. Extraction solvent (500µl) acetonitrile/methanol (50:50) containing the internal standard (5 ng/mL of Nilotinib_13C_2H3) was added to the sample. The mixture was vortexed and incubated for 20 min on ice to accelerate protein precipitation and dialysis through 25µm membranes to obtain unbound or free nilotinib. After incubation, the samples were vortexed and centrifuged at 13,000 rpm for 20 minutes at 4°C. The supernatant containing unbound nilotinib was freeze-dried using speed vacuum and reconstituted in 200µL of methanol: water (50:50) and processed by mass spectrometry (MS).

The samples were resolved on an Acquity UPLC BEH C18 1.7µm, 2.1×50 mm column online with a triple quadrupole mass spectrometer (Xevo-TQ-S, Waters Corporation, USA) operating in the multiple reaction monitoring (MRM) mode as we previously described[1].

Quantification of dopamine metabolites DOPAC and HVA by LC-MS/MS. Concentrations of DOPAC and HVA in the CSF samples were measured by ultrahigh performance liquid chromatography with electrospray tandem MS (UHPLC-MS/MS) following derivatization with benzoyl chloride as previously described[3,4]. Briefly, the UHPLC-MS/MS system included a

PAL autosampler (CTC Analytics, Switzerland), an Advance UHPLC pump, an EVOQ Elite triple quadrupole mass spectrometer (Bruker Daltonics, USA) equipped with an electrospray ionization (EPI) source operating in a positive mode at +4500 V. The mobile phase B was acetonitrile, 0.1% formic acid. The gradient elution was as follows (min - %A/%B): 0 – 98/2; 0.2 – 98/2; 6 – 30/70; 7.5 – 30/70; 7.6 – 98/2; 8.2 – 98/2, the flow rate was 400µl/min. The stable isotope labeled DOPAC-d5 and HVA-d3 (Toronto Research Chemicals, Canada) were used as the internal standards. The derivatization procedure was performed at room temperature. To a 10µl volume of CSF sample or a calibration standard in CSF (148 mM NaCl, 4 mM KCl, 0.8 mM $MgCl_2$, 1.4 $CaCl_2$, 1.2 mM Na_2HPO_4, 0.3 mM NaH_2PO_4, pH 7.2) the following reagents were pipetted and the mixture was vigorously shaken after each pipetting step: 6 µl of the internal standard mixture (1 µM of each deuterated analyte standard in water), followed by 6 µl of 0.1 M sodium tetraborate buffer and 6 µl benzoyl chloride (1% v/v in acetonitrile, prepared fresh daily). The calibration curves were constructed in the range of 0.25 – 16384 nM. The levels of DOPAC and HVA in the CSF samples were measured by Pronexus Analytical AB, Bromma, Sweden and was verified by ELISA as detailed below.

Alpha-synuclein ELISA. To avoid freeze-thaw cycles, immediately after LP and blood draws, 15 ml CSF and 5 ml plasma were aliquoted on ice into 0.5 ml tubes and stored at –80°C. Fresh aliquots were used to perform ELISA. All samples were analyzed side-by-side using same reagents. Solid phase total alpha-synuclein sandwich ELISA (Cat#SIG38974, Biolegend) was performed on CSF. Total alpha-synuclein rabbit monoclonal antibody (amino acids 118–123) was coated on the microwells and 200µl CSF (diluted 1:10) was added to designated wells. After overnight sample incubation at 4°C, alpha-synuclein was captured by the coated antibody. After washing, a biotinylated mouse monoclonal alpha-synuclein (amino acids 103–107) detection

antibody was added to each well to detect the captured alpha-synuclein (amino acids 118–123). Samples were incubated with 50µl of detection antibody for 2 hours at room temperature. After washing, 200µl of streptavidin HRP was added and incubated for 1 hour at room temperature to recognize the bound biotinylated detection antibody. Samples were then washed and incubated with 100µl of chemiluminescent substrates. Plates were shaken for 10–15 seconds and read immediately by a luminometer.

Oligomeric alpha-synuclein ELISA. Solid phase human alpha-synuclein oligomer sandwich ELISA (Cat# MBS730762, Mybiosource) was performed on CSF. 50 µl of standards or CSF samples were added to the appropriate wells. 5 µl of balance solution and 100 µl of conjugate was dispensed into samples only and mixed well. Sample solution was mixed well and incubated for one hour at 37°C. After washing, samples were incubated with 50 µl substrate-A and 50 µl Substrate-B per well, including blank control well. Sample solution was incubated for 10-15 minutes at 37°C before 50 µl of stop solution was added to each well including blank control well. To determine the Optical Density (O.D.), samples were read at 450 nm using a microplate reader.

Clinical Assessments

All participants were tested in the "ON" state less than 2 hours since the last dose of levodopa. A single rater conducted all clinical exams in all participants across all study visits and ON state was also verified with the participant and the objective report of study partner and study investigator/rater. Motor assessments were performed at baseline, 6, 12, and 15 months via PDQ39, TUG, MoCA and MDS-UPDRS.

eReferences

1. Pagan FL, Hebron ML, Wilmarth B, et al. Pharmacokinetics and pharmacodynamics of a single dose Nilotinib in individuals with Parkinson's disease. *Pharmacol Res Perspect.* 2019;7(2):e00470.
2. Pagan F, Hebron M, Valadez EH, et al. Nilotinib Effects in Parkinson's disease and Dementia with Lewy bodies. *J Parkinsons Dis.* 2016;6(3):503-517.
3. Song P, Mabrouk OS, Hershey ND, Kennedy RT. In vivo neurochemical monitoring using benzoyl chloride derivatization and liquid chromatography-mass spectrometry. *Anal Chem.* 2012;84(1):412-419.
4. Rascol O, Fitzer-Attas CJ, Hauser R, et al. A double-blind, delayed-start trial of rasagiline in Parkinson's disease (the ADAGIO study): prespecified and post-hoc analyses of the need for additional therapies, changes in UPDRS scores, and non-motor outcomes. *Lancet Neurol.* 2011;10(5):415-423.

eFigure 1. Data represent the effects of 1-year nilotinib treatment on Abl activation via tyrosine phosphorylation and plasma dopamine metabolites compared to placebo. Graphs represent differences between study groups at 12 months in **A).** Levels of pan-tyrosine Abl (phosphorylation) in the CSF. **B**). Levels of Abl phosphorylation at tyrosine 412 in the CSF **C**). Levels of pan-tyrosine Abl (phosphorylation) in the plasma, **D**) Levels of Abl phosphorylation at tyrosine 412 in the plasma, **E**) Ratio of tyrosine412/pan-tyrosine in CSF, and **F**). Ratio of tyrosine412/pan-tyrosine in plasma. These data show that nilotinib has no effect on CSF or plasma Abl activity. Graphs represent plasma levels of **G**). HVA and, **H**) DOPAC indicating a significant increase in DOPAC alone. Graph **I**) represents the level of CSF TREM2, indicating no changes. Nilo: Nilotinib, Abl: Abelson, TREM2: Triggered Receptors on Myeloid Cells, Tyr: Tyrosine, HVA: Homovanillic Acid, DOPAC: 3,4-Dihydroxyphenylacetic acid, ABS: Absorption.

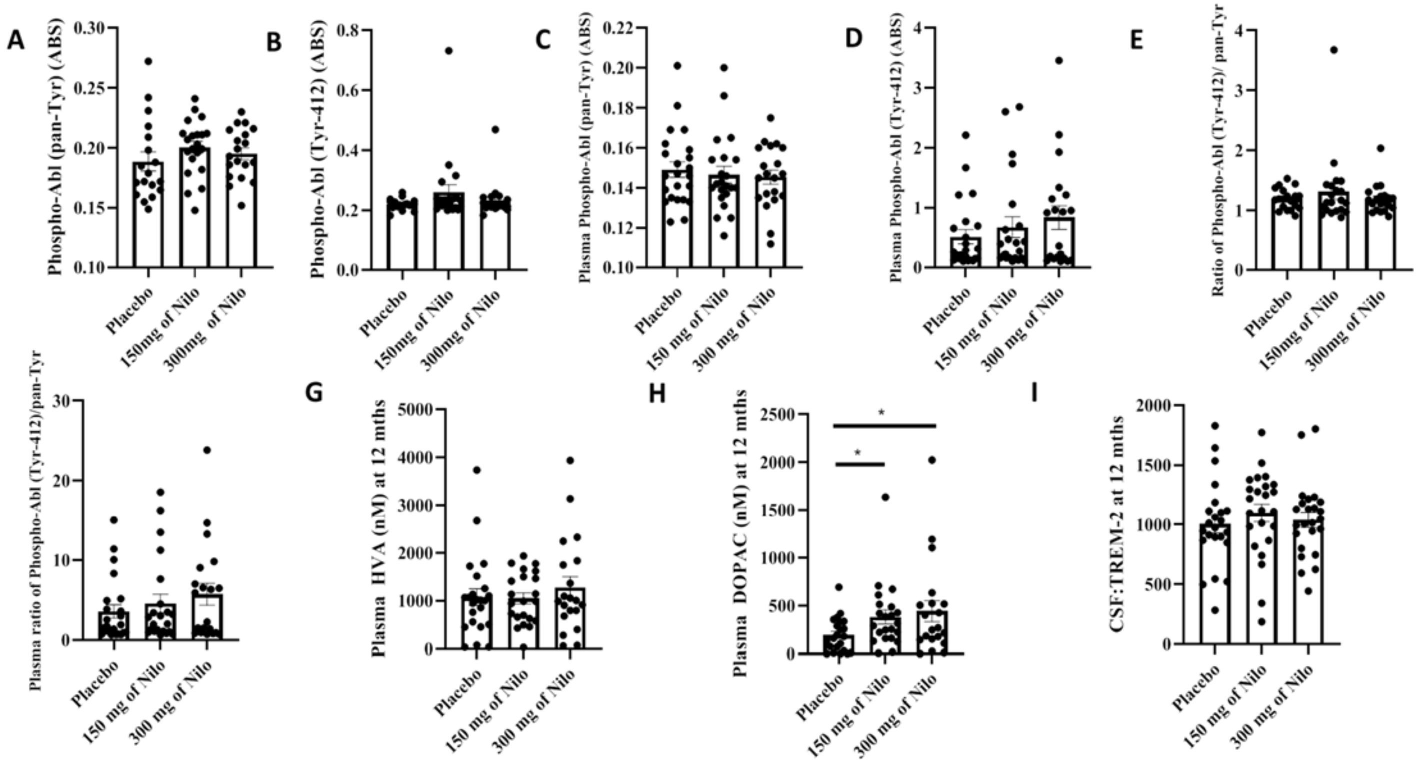

eFigure 2. Clinical effects of nilotinib in PD using the Movement Disorders Society-Unified Parkinson Disease Rating Scale (MDS-UPDRS) and Parkinson's Disease Questionnaire (PDQ)-39. Graphs represent nilotinib effects versus placebo in moderately advanced PD patients tested "ON" time and receiving optimal PD-medications. **A**). Shows UPDRS- III motor , **B**). Total UPDRS I-III, and **C**). (PDQ)-39 summary index (SI) in placebo, 150mg nilotinib and 300 mg nilotinib groups. MDS-UPDRS: Movement Disorders Society- Unified Parkinson's Disease Rating Scale; PDQ-39, Parkinson's Disease Questionnaire- 39 items, Nil: nilotinib.

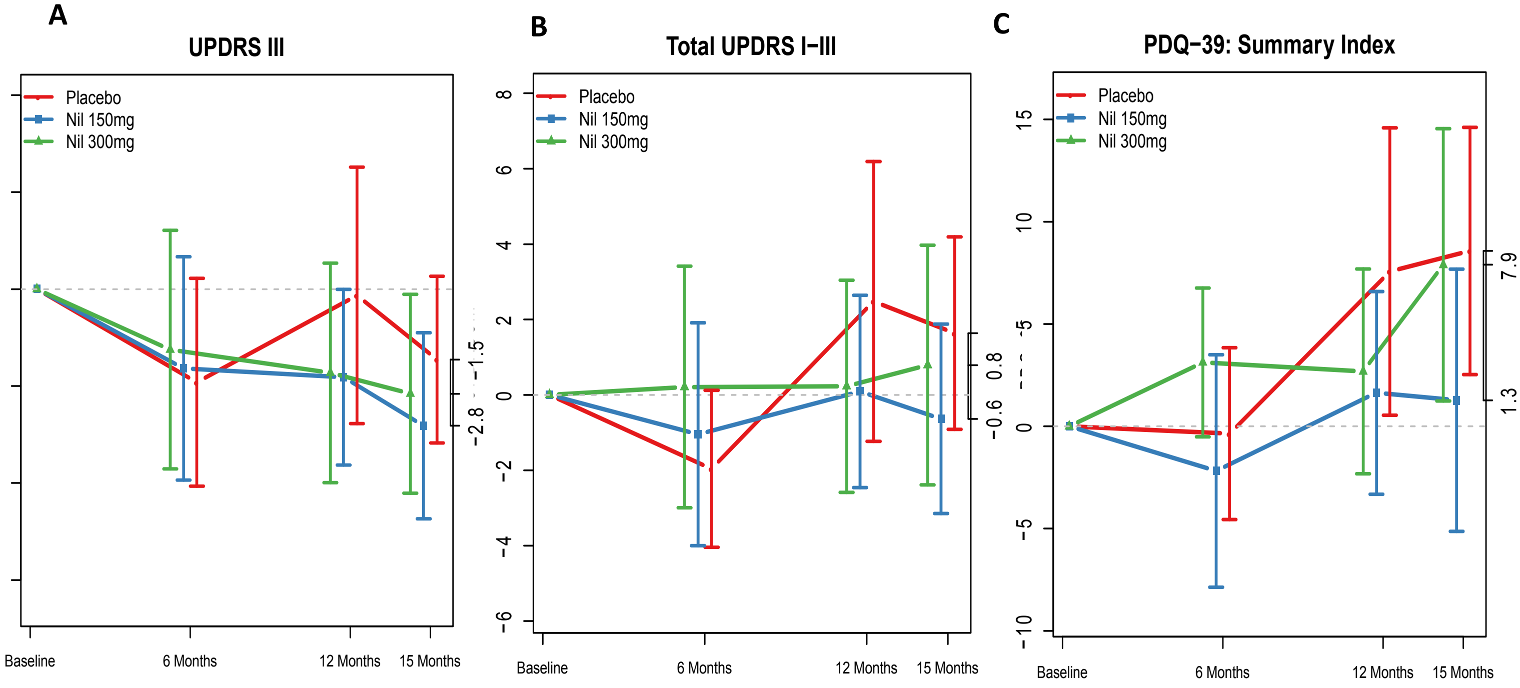

eTable 1. Summary of EKG values for all participants throughout all study visits showing no QTc prolongation in the placebo group. The baseline QTc range for inclusion into this study was 350-460 ms. An SAE was defined as QTc prolongation ≥ 60 ms from baseline of individual participants AND to a value ≥ 480 ms or QTcF prolongs ≥ 500 ms. ms: millisecond; m: month

Placebo (n=25)																		
	Screening	Baseline	0.5 m	1 m	1.5 m	2 m	3m	4 m	5m	6 m	7 m	8 m	9 m	10 m	11 m	miscellaneous visit	12 m	15 m
Nil-PD-001	418	409	410	408	412	391	403	418	413	405	426	417	416	413	415	413	425	430
Nil-PD-006	412	414	412	406	394	396	412	411	394	418	415	420	413	404	405	390	406	405
Nil-PD-007	455	458	457	436	457	450	440	442	446	445	452	449	451	462	431	447	438	445
Nil-PD-009	408	397	422	397	422	414	438	412	406	398	388	399	389	395	397	396	402	401
Nil-PD-010	428	420	413	410	414	426	420	420	422	420	445	426	422	419	434	436	425	430
Nil-PD-013	421/415	366; 378	377; 370	375; 362	384	391	407	393	382; 394	380	383	381	409	418	351	395	403	393
Nil-PD-021	423	419	425; 436	429	431	441	418	431	453	430	431	439	441	442	436	427	438	439
Nil-PD-024	456; 442	454	461	454	465	458; 465	457	443; 472	453	479	442	454	455	477	462	442	449	455
Nil-PD-025	418	421	427	424	420	428	404	420	424	414	413	430	423	423	420	426	430	420

**Nil-PD-032**	434	N/A	418	397	405	405	444	448	439	423	416	447	410	419	452	411	418	397
**Nil-PD-034**	426	430	426	406	418	432	420	424	455	419	437	435	419	426	422	444	414	437
**Nil-PD-035**	420	399	421	405	413	416	427	418	429	414	404	414	414	412	415	426	407	433
**Nil-PD-037**	399	435	431	430	447	460	445	430	418	427	447	439	421	432	421	446	440	422
**Nil-PD-042**	428	394	406	396	422	392	422	420	411	388	463	422	416	403	413	411	408	438
**Nil-PD-046**	436	427	441	434	436	429	425	441	422	426	436	440	427	445	419	430	440	428
**Nil-PD-047**	428	455	418	420	448	436	435	425	406	412	444	438	428	421	423	N/A	434	436
**Nil-PD-051**	416	394	405	444	398	419	407	412	397	396	393	365	394	416	385	N/A	409	402
**Nil-PD-053**	431	432	454	446	436	435	432; 437	440	432	440	440	438	438	442	**OUT OF STUDY**			
**Nil-PD-056**	439	431	454	432	430	433	434	426	432	428	442	437	420	438	422	N/A		441
**Nil-PD-**	403	417	415	401	413	402	415	409	413	404	402	393	390	396	404	396	396	406

**057**																		
**Nil-PD-059**	420	407	413	404	407	412	405	404	420	399	397	404	391	387	407	406	409	416
**Nil-PD-066**	409	424	427	440	422	419	409	427	405	430	430	433	424	**OUT OF STUDY**				
**Nil-PD-067**	435	431	463	438	447	445	455	423	422	441	440	435	440	432	418	435	421	439
**Nil-PD-071**	423; 412	426	412; 414	401	424	432	414	412	440	411	409	424	441	415	416	N/A	418	397
**Nil-PD-074**	381; 379	379	392	405	397	392	381	392	395	396	403	392	399	403	406	N/A	416	**OUT OF STUDY**

eTable 2. Summary of EKG values for all participants throughout all study visits showing no QTc prolongation in the 150mg nilotinib group The baseline QTc range for inclusion into this study was 350-460 ms. An SAE was defined as QTc prolongation ≥ 60 ms from baseline of individual participants AND to a value ≥ 480 ms or QTcF prolongs ≥ 500 ms. ms: millisecond: m: month.

150mg nilotinib (n=25)																	
	**Screening**	**Baseline**	**0.5 m**	**1 m**	**1.5 m**	**2 m**	**3m**	**4 m**	**5 m**	**6 m**	**7m**	**8 m**	**9 m**	**10 m**	**11 m**	**miscellaneous visit**	**12 m**	**15 m**
**Nil-PD-002**	414	435	444	440	421	409	423	426	425	418	434	429	443	424	432	420	406; 419	401
**Nil-PD-004**	425	404	411	434	431	428	427	430	419	409	396	416	414	437	419	429	434	422
**Nil-PD-011**	430	436	466/469	481	455	412	454	OUT OF STUDY										
**Nil-PD-012**	419	436	440	406	442	430	428	423	433	420	422	436	436	440	435	416	417	413
**Nil-PD-014**	396	407	426	432	406	402	417	420	440	427	400	425	404	425	444	412	429	420

**Nil-PD-018**	386	391	405	388	404	387	397	398	382	392	402	401	398	391	381	397	381	407
**Nil-PD-019**	451	436	455	441	462	455	460	447	450	454	454	454	457	449	461; 456	453	456	470
**Nil-PD-020**	421	428	432	441	440	441	434	445	439	427	429	439	449	450	451	454	462	450
**Nil-PD-026**	401	406	414	399	394	415	388	382	392	400	403	400	392	396	399	408	419	410
**Nil-PD-028**	432	423; 424	435;447;433	427	434	438	443	434	423	431	431	435	430	450	433	457	445	411
**Nil-PD-033**	453	442	451	461	444	443	443	461	455	459	458	452	452	474	449	448	442	454
**Nil-PD-036**	386; 384; 384	384	390	399	396	400	401	417	393	402	395	384	404	398	408	393	384	389

**Nil-PD-038**	456	446	429	443	430	418	440	438	447	436	431	441	434	423	429	441	434	425
**Nil-PD-039**	412	399	414	422	409	418	416	428	399	400	418	406	403	407	409	402	408	399
**Nil-PD-041**	383	364	420; 445	386; 367	422	425	394	408	375	398	408; 402	385	366	387	383	397	420	408
**Nil-PD-044**	433	432	435	422	431	458	444	433	417	434	428	397	402	431	436	420	427	428
**Nil-PD-049**	426	426	417	410; 403	402	428	410	421	431	399		408	386	415	410	N/A	425	419
**Nil-PD-050**	421	407	430	433	429	413	435	423	418	424	425	432	418	394	427	N/A	431	432
**Nil-PD-055**	424	426	443	435	434	427	440; 430	427	427	435	431	449	440	449	439	442	428	423

**Nil-PD-060**	391	378	380	385	399	418	420	390	406	408	412	407	385	430	391	404	421	384
**Nil-PD-061**	438	416	447	**OUT OF STUDY**														
**Nil-PD-063**	409	401	405	384	407	417	406	425	443	400	414	401	428	415	396	418	404	404
**Nil-PD-069**	442	446	431	435	425	431	431	423	425	414	451	422	431	429	422	N/A	437	436
**Nil-PD-072**	420	403	414	417	408	419	405	417	413	424	428	403	411	409	422	406	419	428
**Nil-PD-073**	445	436	434	443	487	491	418	**OUT OF STUDY**										

eTable 3. Summary of EKG values for all participants throughout all study visits showing no QTc prolongation in the 300mg nilotinib group. The baseline QTc range for inclusion into this study was 350-460 ms. An SAE was defined as QTc prolongation ≥ 60 ms from baseline of individual participants AND to a value ≥ 480 ms or QTcF prolongs ≥ 500 ms. ms: millisecond; m: month.

300mg nilotinib (n=25)																		
	**Screening**	**Baseline**	**0.5 m**	**1 m**	**1.5 m**	**2 m**	**3m**	**4 m**	**5m**	**6 m**	**7 m**	**8 m**	**9 m**	**10 m**	**11 m**	**miscellaneous visit**	**12 m**	**15 m**
**Nil-PD-003**	418	412	417	427	414	404	413	417	415	403	416	435	417	429	414	412	421	416
**Nil-PD-005**	420/405	449/441	408	435	430	420	416	433	411	405	413	397	420	406	398	445	435	416
**Nil-PD-008**	436	437	445	447	431	419	442	432	444	432	435	451	435	431	446	439	425	429
**Nil-PD-015**	423	411	416	415	409	424	411	424	426	418	407	405	399	406	434	415	407	416
**Nil-PD-016**	429	448	455	442	438	456	438	455	424; 421	431	437	430	452	448	434	378	463	414
**Nil-PD-017**	436	407/415	440	414	429	433	427	431	426	434	421	441	419	440	437	430	425	**OUT OF STUDY**
**Nil-PD-022**	428	421	416	444	442	454	418	454; 431	440	431	420	436	419	451	437	440	431	432
**Nil-PD-**	415	401; 404	399	401	414	413	404	409	414	411	399	401	401	416	409	406	394	404

**023**																		
**Nil-PD-027**	397	397; 412	403	388	416	449	440; 403	384	434	417; 409	404	422	384	422	407	414	409	442
**Nil-PD-029**	420	426	410	425	414	N/A	410	413	428	418	413	414	411	412	418	414	409	416
**Nil-PD-030**	426	416	441	428	435	430	434	415	438	**OUT OF STUDY**								
**Nil-PD-031**	452	447	464	460	462	461; 462	468	461	461	448	430	452	459	437	450	450	442	452
**Nil-PD-040**	450	433	451	477	450	455	433	443	445	440	435	450	472	440	462	443	438	455
**Nil-PD-043**	446	414	438	441	443	450	451	448	465	446	419	451	422	421	442	462	445	453
**Nil-PD-045**	401	409	409	411	400	394	401	394	398	401	410	396	401	416	409	406	409	399
**Nil-PD-048**	435	449	429	442	438	432	424	401; 449	433	445	442	437	426	**OUT OF STUDY**				

**Nil-PD-052**	416	403	424	418	426	411	440	409	428	385	421	419	405	402	420	411	428	408
**Nil-PD-054**	436	451	468	473; 481; 503	N/A	450	445	450	455	448	424	453	437	436	**OUT OF STUDY**			
**Nil-PD-058**	452	425	459	467	467	469	462	462	445	454	452	478	442	446	442	N/A	462	461
**Nil-PD-062**	434	450	458	445	446	437	450	466	459	453	469	477	471	454	463	447	**OUT OF STUDY**	
**Nil-PD-064**	418	426	445	419	425; 430	445	433	437	442	433	417	427	N/A	448	442	N/A	415	432
**Nil-PD-065**	426	407	433	418	425	414	388	406	418	407	406	416	418	419	409	431	410	411
**Nil-PD-068**	444	452	457	450	456	448	452	459	454	444	460	457	455	445	469	N/A	442	**OUT OF STUDY**
**Nil-PD-070**	419; 386	436	440	450	438	442	427	450	444	444	438	450	446	442	433	456	448	455
**Nil-PD-075**	437	433	440	452	424	425	412	415	404	415	432	421	412	410	455; 429; 436	N/A	410	426

eTable 4. Levels of exploratory **A**). CSF and **B**). plasma biomarkers are detailed as mean±SD, 95% CI and 90% CI and p=values comparing 150mg nilotinib and 300 mg nilotinib groups to placebo at 12 months. HVA: Homovanillic Acid, DOPAC: 3,4-Dihydroxyphenylacetic acid, TREM-2: Triggered Receptors on Myeloid Cells, Abl: Abelson. FDR: False Discovery Rate

A)

	**CSF**											
	**12 months: Placebo (n=21)**		**12 months: 150 mg nilotinib (n=20)**					**12 months: 300 mg nilotinib (n=20)**				
	**Mean ± SD**	**[95% CI of mean]**	**Mean ± SD**	**[95% CI of mean]**	**p value (FDR) Placebo vs 150 mg nilotinib**	**Difference between means (B - A) ± SEM**	**[90% CI]**	**Mean ± SD**	**[95% CI of mean]**	**p value (FDR) Placebo vs 300 mg nilotinib**	**Difference between means (B - A) ± SEM**	**[90% CI]**
**Total alpha-syncline (pg./ml)**	752.2 ± 340.1	[593.1 - 911.4]	833.9 ± 746.4	[503 - 1165]	0.32 (0.32)	81.66 ± 176.4	-217.7 to 381.0	114 ± 15	[419.1 - 1869]	0.14 (0.32)	391.9 ± 354.7	-218.6 to 1002.0
**Oligomeric alpha-syncline (pg./ml)**	0.2141 ± 0.07	[0.176 - 0.25]	0.16 ± 0.06	[0.1381 - 0.2017]	0.03 (0.09)	-0.044 ± 0.023	-0.083 to - 0.005	0.19 ± 0.05	[0.170 - 0.22]	0.22 (0.32)	-0.016 ± 0.022	-0.053 to 0.020
**Ratio of oligomeric/Total alpha-syncline**	0.0003 ± 0.0001	[0.0002- 0.0004]	0.0002 ± 0.0001	[0.0001 - 0.0003]	0.04 (0.09)	-0.0001± 6.58e-5	-0.0002 to - 0.000	0.0004 ±0.0003	[0.0002 - 0.0005]	0.44 (0.47)	1.34e-005 ± 9.03e-5	-0.0001 to 0.0002
**HVA (nM)**	393.0 ± 241.6	[283.1 - 503]	552.9 ± 345.6	[399.6 - 706.1]	0.04 (0.09)	159.8 ± 90.60	7.043 to 312.6	479.7 ± 444.6	[271.6 - 687.8]	0.22 (0.32)	86.64 ± 112.5	-104.6 to 277.9
**DOPAC (nM)**	5.730± 3.39	[4.143 - 7.316]	10.60 ± 8.55	[6.806 - 14.4]	0.01 (0.08)	4.872 ± 1.976	1.510 to 8.233	13.25 ± 13.03	[7.151 - 19.34]	0.01 (0.06)	7.519 ± 3.010	2.35 to 12.69
**Total tau (pg./ml)**	198.2 ± 83.92	[157.8 - 238.7]	176.9 ± 100.2	[131.3 - 222.5]	0.23 (0.26)	-21.34 ± 29.14	-70.47 to 27.80	169.0 ± 103.2	[117.7 - 220.3]	0.17 (0.32)	-29.23 ± 31.01	-81.72 to 23.26
**phosphor-tau (181) (pg./ml)**	32.09 ± 16.17	[24.3 - 39.88]	22.05 ± 10.36	[17.33 - 26.76]	0.01 (0.08)	-10.04 ± 4.343	-17.41 to - 2.67	20.04 ± 8.37	[15.87 - 24.2]	0.003 (0.05)	-12.05 ± 4.202	-19.21 to -4.90
**Ratio of p-tau (181)/ total tau**	0.16± 0.05	[0.14 - 0.19]	0.13 ± 0.06	[0.10 - 0.16]	0.03 (0.09)	-0.033 ± 0.018	-0.064 to - 0.003	0.13 ± 0.05	[0.10 - 0.16]	0.03 (0.13)	-0.034± 0.018	-0.064 to - 0.004
**TREM-2 (pg./ml)**	1005 ± 360.2	[849.4 - 1161]	1099 ± 362.1	[946.6 - 1252]	0.18 (0.23)	94.35 ± 105.4	-82.61 to 271.3	1041 ± 314.0	[908 - 1173]	0.36 (0.43)	35.49 ± 98.73	-130.4 to 201.4

**Phospho-Abl (Tyr-412) (ABS)**	0.22± 0.01	[0.21 - 0.22	0.26 ± 0.11	[0.20- 0.31]	0.06 (0.10)	0.039 ± 0.025	-0.004 to 0.083	0.23 ± 0.05	[0.20 - 0.25]	0.22 (0.32)	0.011± 0.014	-0.013 to 0.034
**Phospho-Abl (pan-Tyr) (ABS)**	0.18± 0.03	[0..17 - 0.20]	0.20 ± 0.023	[0.189- 0.21]	0.11 (0.17)	0.011 ± 0.009	-0.004 to 0.026	0.19± 0.02	[0.18- 0.20]	0.24 (0.32)	0.006 ± 0.009	-0.009 to 0.021
**Ratio phospho-Abl Tyr-412/ pan-Tyr**	1.192 ± 0.17	[1.10 - 1.27]	1.315 ± 0.58	[1.04 - 1.58]	0.18 (0.23)	0.123 ± 0.135	-0.108 to 0.354	1.18 ± 0.25	[1.07 - 1.30]	0.47 (0.47)	-0.005 ± 0.068	-0.119 to 0.110

B)

**Plasma**												
	**12months : Placebo (n=21)**		**12months: 150 mg nilotinib (n=20)**					**12months: 300 mg nilotinib (n=20)**				
	**Mean ± SD**	**[95% CI of mean]**	**Mean ± SD**	**[95% CI of mean]**	**p value (FDR) Placebo vs 150 mg nilotinib**	**Difference between means (B - A) ± SEM**	**[90 % CI]**	**Mean ± SD**	**[95% CI of mean]**	**p value (FDR) Placebo vs 300 mg nilotinib**	**Difference between means (B - A) ± SEM**	**[90 % CI]**
**HVA (nM)**	1077 ± 837.7	[715 - 1439]	1049 ± 531.3	[813. 2 - 1284]	0.44 (0.44)	-28.49 ± 208.2	- 379.6 to 322.6	1282 ± 1002	[813. 1 - 1751]	0.23 (0.23)	205.0 ± 284.2	- 274.3 to 684.4
**DOPAC (nM)**	194.6 ± 177.5	[115. 9 - 273.3]	382.4 ± 341.1	[231. 1 - 533.6]	0.01 (0.05)	187.8 ± 81.99	48.84 to 326.7	443.1 ± 494.8	[211. 5 - 674.7]	0.02 (0.10)	248.5 ± 116.9	48.24 to 448.7
**Phospho -Abl (Tyr-412) (ABS)**	0.51 ± 0.56	[0.27 - 0.75]	0.675 3 ± 0.80	[0.31- 1.03]	0.22 (0.40)	0.159 ± 0.208	- 0.192 to 0.510	0.83 ± 0.88	[0.42 - 1.25]	0.08 (0.15)	0.32 ± 0.23	- 0.070 to 0.713
**Phospho -Abl (pan-Tyr) (ABS)**	0.14± 0.01	[0.14- 0.15]	0.14 ± 0.01	[0.13 - 0.15]	0.33 (0.41)	-0.003 ± 0.006	- 0.012 to 0.007	0.14 ± 0.01	[0.13 - 0.15]	0.23 (0.23)	-0.003 ± 0.005	- 0.013 to 0.005
**Ratio phospho -Abl Tyr-412/ pan-Tyr**	3.58 ± 3.95	[1.87 - 5.29]	4.58 ± 5.3	[2.20 - 6.96]	0.24 (0.40)	0.998 ± 1.411	- 1.379 to 3.376	5.73 ± 6.1	[2.87 - 8.58]	0.09 (0.15)	2.151 ± 1.594	- 0.551 to 4.852

eTable 5. Pairwise comparison of clinical endpoints using mean differences based on Raw-value at 6, 12 and 15 months compared to baseline. MoCA: Montreal Cognitive Assessment; MDS-UPDRS: Movement Disorders Society- Unified Parkinson's Disease Rating Scale; PDQ-39, Parkinson's Disease Questionnaire- 39 items. FDR: False Discovery Rate

		Mean diff (baseline – 6 months)		Mean diff (6- 12 months)		Mean diff (baseline – 12 months)		Mean diff (baseline-15 months)		Mean diff (12-15months)	
**Endpoint**	Group	Est. (95% CI)	P-value (FDR)	Est. (95% CI)	P-value (FDR)	Est. (95% CI)	P-value (FDR)	Est. (95% CI)	P-value (FDR)	Est. (95% CI)	P-value (FDR)
**MoCA**	Placebo	-0.4 (-1.07, 0.27)	0.37 (0.93)	-0.44 (-1.42, 0.55)	0.73 (0.81)	-0.91 (-1.91, 0.09)	0.12 (0.27)	-0.18 (-0.69, 0.33)	0.48 (0.6)	0.64 (-0.12, 1.39)	0.13 (0.82)
	150mg nilotinib	0.18 (-0.34, 0.71)	0.6 (0.85)	-0.36 (-0.87, 0.14)	0.21 (0.48)	-0.18 (-0.78, 0.42)	0.82 (0.91)	0.04 (-0.67, 0.76)	0.64 (0.69)	0.23 (-0.3, 0.76)	0.46 (0.77)
	300mg nilotinib	**-1.04 (-1.65, -0.44)**	**0 (0.04)**	-0.59 (-1.23, 0.05)	0.09 (0.5)	**-1.46 (-2.2, -0.71)**	**0 (0.02)**	-0.47 (-1.13, 0.19)	0.23 (0.38)	0.79 (0.3, 1.28)	0.01 (0.09)
**Time Up & Go**	Placebo	0.5 (-1.06, 2.06)	0.64 (0.98)	2.24 (-1.76, 6.23)	0.44 (0.61)	2.73 (-0.73, 6.18)	0.17 (0.27)	**2.64 (0.82, 4.45)**	**0 (0.02)**	-0.29 (-2.64, 2.07)	0.23 (0.82)
	150mg nilotinib	0.15 (-0.54, 0.84)	0.77 (0.9)	0.55 (-0.89, 1.99)	0.62 (0.78)	1.54 (-0.29, 3.38)	0.12 (0.4)	1.36 (-0.37, 3.1)	0.18 (0.45)	-0.18 (-1.6, 1.24)	0.83 (1)
	300mg nilotinib	1.2 (-0.08, 2.47)	0.11 (0.24)	1.36 (-0.16, 2.87)	0.11 (0.5)	**2.76 (0.73, 4.79)**	**0.01 (0.03)**	**2.53 (0.43, 4.62)**	**0.01 (0.05)**	-0.22 (-1.79, 1.34)	1 (1)
**MDS-UPDRS I**	Placebo	0 (-0.49, 0.49)	1 (1)	0.04 (-0.55, 0.64)	0.95 (0.95)	0.09 (-0.54, 0.71)	0.75 (0.75)	0.14 (-0.47, 0.74)	0.7 (0.7)	0.04 (-0.51, 0.6)	0.95 (0.95)
	150mg nilotinib	0 (-0.5, 0.5)	0.98 (0.98)	0.23 (-0.09, 0.54)	0.18 (0.48)	0.23 (-0.33, 0.79)	0.38 (0.63)	0.46 (-0.01, 0.92)	0.08 (0.25)	0.23 (-0.24, 0.69)	0.45 (0.77)
	300mg nilotinib	0.38 (-0.09, 0.84)	0.12 (0.24)	0 (-0.32, 0.32)	1 (1)	0.27 (-0.28, 0.82)	0.32 (0.63)	0.26 (-0.5, 1.02)	0.54 (0.6)	0.1 (-0.32, 0.53)	0.82 (1)
**MDS-UPDRS II**	Placebo	-0.04 (-1.28, 1.2)	0.95 (1)	**2.39 (0.84, 3.94)**	**0.01 (0.02)**	2.52 (0.89, 4.15)	0.01 (0.1)	**2.96 (1.64, 4.27)**	**0 (0.01)**	0.64 (-0.81, 2.09)	0.55 (0.82)
	150mg nilotinib	0.59 (-0.55, 1.73)	0.38 (0.76)	1.09 (-0.04, 2.22)	0.13 (0.48)	1.68 (0.5, 2.87)	0.01 (0.14)	1.73 (0.48, 2.97)	0.02 (0.08)	0.04 (-1.12, 1.21)	1 (1)
	300mg nilotinib	1.08 (0.21, 1.96)	0.03 (0.14)	0.96 (-0.23, 2.14)	0.15 (0.5)	**1.68 (0.64, 2.73)**	**0.01 (0.03)**	**2.68 (1.41, 3.96)**	**0 (0.01)**	0.68 (-0.24, 1.61)	0.19 (0.58)
**MDS-UPDRS III**	Placebo	-1.92 (-4.07, 0.23)	0.08 (0.32)	2.35 (0, 4.69)	0.06 (0.11)	-0.13 (-2.79, 2.53)	0.64 (0.71)	-1.46 (-3.18, 0.27)	0.13 (0.34)	-0.91 (-2.97, 1.15)	0.57 (0.82)
	150mg nilotinib	-1.64 (-3.95, 0.68)	0.15 (0.76)	-0.18 (-2.12, 1.75)	1 (1)	-1.82 (-3.64, 0)	0.12 (0.4)	-2.82 (-4.75, -0.89)	0.01 (0.08)	-1 (-2.52, 0.52)	0.07 (0.73)

© 2019 Pagan FL et al. *JAMA Neurology*

	300mg nilotinib	-1.25 (-3.72, 1.22)	0.42 (0.6)	0.14 (-1.81, 2.08)	0.81 (0.9)	-1.73 (-4, 0.55)	0.12 (0.3)	-2.16 (-4.22, -0.1)	0.07 (0.18)	-0.32 (-2.46, 1.83)	0.9 (1)
**MDS-UPDRS IV**	Placebo	0.08 (-0.8, 0.96)	0.94 (1)	-0.3 (-1, 0.39)	0.49 (0.61)	-0.35 (-1.55, 0.86)	0.58 (0.71)	0.46 (-0.46, 1.36)	0.66 (0.7)	0.41 (-0.24, 1.06)	0.25 (0.82)
	150mg nilotinib	0.32 (-0.32, 0.96)	0.35 (0.76)	0.14 (-0.56, 0.83)	0.75 (0.83)	0.46 (-0.24, 1.15)	0.27 (0.63)	0.23 (-0.4, 0.86)	0.56 (0.69)	-0.23 (-0.74, 0.29)	0.4 (0.77)
	300mg nilotinib	-0.58 (-1.53, 0.36)	0.33 (0.56)	0.23 (-0.62, 1.07)	0.69 (0.86)	-0.23 (-1.17, 0.71)	0.69 (0.86)	0.05 (-1.36, 1.47)	0.58 (0.6)	0.32 (-0.51, 1.14)	0.42 (0.71)
**MDS-UPDRS I-III**	Placebo	-1.96 (-4.05, 0.13)	0.09 (0.32)	4.78 (1.2, 8.36)	0.03 (0.08)	2.48 (-1.25, 6.21)	0.19 (0.27)	1.64 (-0.93, 4.2)	0.23 (0.38)	-0.23 (-3.07, 2.61)	0.51 (0.82)
	150mg nilotinib	-1.04 (-4.02, 1.92)	0.26 (0.76)	1.14 (-1.14, 3.42)	0.29 (0.48)	0.09 (-2.48, 2.66)	0.96 (0.96)	-0.64 (-3.16, 1.89)	0.69 (0.69)	-0.73 (-2.88, 1.43)	0.43 (0.77)
	300mg nilotinib	0.21 (-3.01, 3.43)	0.75 (0.84)	1.09 (-1.58, 3.76)	0.3 (0.62)	0.23 (-2.6, 3.06)	0.94 (0.94)	0.79 (-2.41, 3.98)	0.56 (0.6)	0.47 (-1.93, 2.87)	0.5 (0.71)
**MDS-UPDRS I-IV**	Placebo	-1.88 (-4.38, 0.62)	0.1 (0.32)	4.48 (0.95, 8.01)	0.04 (0.08)	2.13 (-1.42, 5.68)	0.17 (0.27)	2.09 (-0.68, 4.86)	0.18 (0.35)	0.18 (-2.83, 3.2)	0.67 (0.83)
	150mg nilotinib	-0.73 (-3.87, 2.42)	0.35 (0.76)	1.27 (-1.37, 3.92)	0.32 (0.48)	0.55 (-2.23, 3.32)	0.68 (0.86)	-0.41 (-3.04, 2.22)	0.68 (0.69)	-0.96 (-3.16, 1.25)	0.4 (0.77)
	300mg nilotinib	-0.38 (-3.67, 2.92)	0.99 (0.99)	1.32 (-1.74, 4.37)	0.31 (0.62)	0 (-2.82, 2.82)	0.92 (0.94)	0.84 (-2.82, 4.5)	0.6 (0.6)	0.79 (-1.98, 3.56)	0.23 (0.58)
**PDQ-39 Summary Index (SI)**	Placebo	-0.36 (-4.58, 3.86)	0.69 (0.98)	**8.17 (3.1, 13.25)**	**0 (0.01)**	7.57 (0.51, 14.62)	0.06 (0.2)	8.57 (2.5, 14.64)	0.02 (0.07)	2.43 (-4.31, 9.17)	0.94 (0.95)
	150mg nilotinib	-2.18 (-7.9, 3.53)	0.51 (0.85)	3.82 (-0.7, 8.34)	0.11 (0.48)	1.64 (-3.34, 6.62)	0.34 (0.63)	1.27 (-5.17, 7.71)	0.65 (0.69)	-0.36 (-5.3, 4.57)	0.85 (1)
	300mg nilotinib	3.12 (-0.53, 6.78)	0.11 (0.24)	0.36 (-4.99, 5.71)	0.64 (0.86)	2.68 (-2.35, 7.71)	0.45 (0.76)	7.89 (1.21, 14.58)	0.03 (0.1)	4.11 (-1.26, 9.48)	0.19 (0.58)
**Emotional Well being**	Placebo	-1.08 (-3.77, 1.6)	0.58 (0.98)	**7.07 (1.62, 12.52)**	**0 (0.01)**	5.99 (0.39, 11.58)	0.05 (0.2)	4.54 (-1.76, 10.85)	0.32 (0.45)	-1.53 (-7.3, 4.24)	0.56 (0.82)
	150mg nilotinib	-0.58 (-5.46, 4.29)	0.81 (0.9)	1.72 (-2.19, 5.62)	0.33 (0.48)	1.14 (-4.02, 6.29)	0.61 (0.86)	0.96 (-3.24, 5.15)	0.54 (0.69)	-0.18 (-4.73, 4.36)	1 (1)
	300mg nilotinib	0 (-4.89, 4.88)	0.53 (0.66)	0.24 (-3.58, 4.06)	0.45 (0.75)	0.23 (-3.79, 4.25)	0.53 (0.76)	4.86 (-0.31, 10.02)	0.12 (0.23)	4.14 (-1.9, 10.19)	0.3 (0.59)

eTable 6. Average values of each clinical endpoint in placebo, 150mg nilotinib and 300mg nilotinib groups represented as mean±SD (95% CI) at baseline, 6, 12 and 15 months. MoCA: Montreal Cognitive Assessment; MDS-UPDRS: Movement Disorders Society- Unified Parkinson's Disease Rating Scale; PDQ-39, Parkinson's Disease Questionnaire- 39 items.

Endpoints	Group	Baseline	6 Months	12 Months	15 Months
MoCA	Placebo	29±0.96 (28.62, 29.38)	28.6±1.89 (27.86, 29.34)	28.09±2.71 (26.98, 29.2)	28.86±1.36 (28.3, 29.43)
	150mg nilotinib	28.8±1.78 (28.1, 29.5)	29.05±1.29 (28.51, 29.59)	28.68±1.46 (28.07, 29.29)	28.91±1.38 (28.33, 29.48)
	300mg nilotinib	29.04±1.59 (28.41, 29.66)	28±1.98 (27.21, 28.79)	27.5±2.35 (26.52, 28.48)	28.42±1.84 (27.6, 29.25)
Time Up & Go	Placebo	10.92±3.35 (9.61, 12.23)	11.38±5.1 (9.34, 13.41)	13.59±10.04 (9.4, 17.79)	13.23±6.6 (10.47, 15.98)
	150mg nilotinib	10.52±2.5 (9.54, 11.5)	10.25±1.89 (9.42, 11.08)	12.23±6.11 (9.67, 14.78)	12.04±5.7 (9.66, 14.43)
	300mg nilotinib	11.28±2.79 (10.19, 12.37)	12.41±4.91 (10.41, 14.42)	13.9±6.49 (11.13, 16.68)	13.79±6.84 (10.72, 16.86)
MDS- UPDRS I	Placebo	2.68±1.77 (1.99, 3.38)	2.68±1.68 (2.02, 3.34)	2.74±1.63 (2.07, 3.4)	2.64±1.62 (1.96, 3.31)
	150mg nilotinib	2.32±1.63 (1.68, 2.96)	2.46±1.34 (1.9, 3.01)	2.68±1.13 (2.21, 3.15)	2.91±1.74 (2.18, 3.64)
	300mg nilotinib	2.4±1.8 (1.69, 3.11)	2.62±1.53 (2.01, 3.24)	2.46±1.44 (1.85, 3.06)	2.63±1.64 (1.89, 3.37)
MDS- UPDRS II	Placebo	12.2±5.11 (10.2, 14.2)	12.16±5.5 (10.01, 14.31)	14.13±6.14 (11.62, 16.64)	14.14±5.81 (11.71, 16.56)
	150mg nilotinib	12.88±4.86 (10.97, 14.79)	13.54±5.37 (11.3, 15.79)	14.64±4.51 (12.75, 16.52)	14.68±4.76 (12.69, 16.67)
	300mg nilotinib	13.16±5.5 (11.01, 15.31)	13.75±5.82 (11.42, 16.08)	14.18±5.17 (12.02, 16.34)	14.95±5.93 (12.28, 17.61)
MDS- UPDRS III	Placebo	28.92±4.77 (27.05, 30.79)	27±4.98 (25.05, 28.95)	29.22±7.17 (26.29, 32.15)	28±5.14 (25.85, 30.15)
	150mg nilotinib	28.52±6.01 (26.16, 30.88)	27.18±7.12 (24.21, 30.16)	27±7.76 (23.75, 30.25)	26±7.08 (23.04, 28.96)
	300mg nilotinib	30.8±4.95 (28.86, 32.74)	29.67±7.34 (26.73, 32.6)	29.55±7.84 (26.27, 32.82)	29.16±7.87 (25.62, 32.7)
MDS- UPDRS IV	Placebo	4.4±2.29 (3.5, 5.3)	4.48±1.96 (3.71, 5.25)	4±2.47 (2.99, 5.01)	4.5±2.24 (3.56, 5.44)

	150mg nilotinib	4±1.89 (3.26, 4.74)	4.54±2.04 (3.69, 5.4)	4.68±1.25 (4.16, 5.2)	4.46±1.5 (3.83, 5.08)
	300mg nilotinib	4.6±2.61 (3.58, 5.62)	3.88±1.7 (3.19, 4.56)	4±2.12 (3.12, 4.88)	4.37±2.09 (3.43, 5.31)
**MDS- UPDRS I-III**	Placebo	43.8±7.64 (40.8, 46.8)	41.84±9.33 (38.18, 45.5)	46.09±13.58 (40.53, 51.64)	44.77±10.69 (40.31, 49.24)
	150mg nilotinib	43.72±9.62 (39.95, 47.49)	43.18±10.68 (38.72, 47.65)	44.32±11.13 (39.67, 48.97)	43.59±10.79 (39.08, 48.1)
	300mg nilotinib	46.36±9.34 (42.7, 50.02)	46.04±12.13 (41.19, 50.9)	46.18±11.62 (41.33, 51.04)	46.74±12.43 (41.15, 52.33)
**MDS- UPDRS I-IV**	Placebo	48.2±9.12 (44.62, 51.78)	46.32±10.45 (42.22, 50.42)	50.09±14.16 (44.3, 55.87)	49.27±11.7 (44.38, 54.16)
	150mg nilotinib	47.72±9.46 (44.01, 51.43)	47.73±11.18 (43.06, 52.4)	49±11.11 (44.36, 53.64)	48.05±10.49 (43.66, 52.43)
	300mg nilotinib	50.96±10.61 (46.8, 55.12)	49.92±12.63 (44.86, 54.97)	50.18±12.14 (45.11, 55.26)	51.1±12.54 (45.47, 56.74)
**PDQ39 – SI**	Placebo	28.12±17.6 (21.22, 35.02)	27.76±17.15 (21.04, 34.48)	34.39±17.96 (27.05, 41.73)	34.91±23.19 (24.99, 44.82)
	150mg nilotinib	28.64±17.6 (21.74, 35.54)	27.95±16.62 (21.01, 34.9)	31.77±14.09 (25.89, 37.66)	31.41±16.96 (24.32, 38.49)
	300mg nilotinib	31.16±23.61 (21.91, 40.41)	31.88±22.32 (22.94, 40.81)	30.95±19.69 (22.73, 39.18)	37.47±22.23 (27.48, 47.47)

eTable 7. Statistical analysis showing in **A**). mixed effects model and Test p-values and **B**). ANOVA test for group differences: p-values. FDR: False Discovery Rate

A)

Endpoints	Placebo vs. 150mg nilotinib	Placebo vs. 300mg nilotinib	baseline vs. 6 months	baseline vs. 12 months	baseline vs. 15 months	Placebo vs. 150mg nilotinib *6 months	Placebo vs. 300mg nilotinib *6 months	Placebo vs. 150mg nilotinib *12 months	Placebo vs. 300mg nilotinib *12 months	Placebo vs. 150mg nilotinib *15 months	Placebo vs. 300mg nilotinib *15 months
**MDS-UPDRS I**	-0.36 (0.433)	-0.28 (0.542)	0 (1)	0.064 (0.804)	0.066 (0.8)	0.04 (0.912)	0.33 (0.356)	0.203 (0.582)	0.194 (0.599)	0.429 (0.25)	0.272 (0.475)
**MDS-UPDRS II**	0.68 (0.661)	0.96 (0.536)	-0.04 (0.949)	2.392 (0)	2.846 (0)	0.643 (0.48)	1.043 (0.242)	-0.698 (0.45)	-0.643 (0.484)	-1.107 (0.234)	-0.212 (0.824)
**MDS-UPDRS III**	-0.4 (0.827)	1.88 (0.307)	-1.92 (0.066)	0.177 (0.868)	-0.954 (0.38)	0.38 (0.801)	0.708 (0.633)	-1.899 (0.216)	-1.551 (0.311)	-1.768 (0.253)	-0.877 (0.579)
**MDS-UPDRS IV**	-0.4 (0.496)	0.2 (0.733)	0.08 (0.843)	-0.322 (0.439)	0.264 (0.532)	0.347 (0.554)	-0.731 (0.205)	0.885 (0.137)	-0.09 (0.88)	0.072 (0.904)	-0.36 (0.556)
**MDS-UPDRS I-III**	-0.08 (0.979)	2.56 (0.399)	-1.96 (0.162)	2.607 (0.072)	1.932 (0.188)	1.023 (0.616)	2.056 (0.304)	-2.407 (0.245)	-2.006 (0.331)	-2.459 (0.239)	-0.835 (0.695)
**MDS-UPDRS I-IV**	-0.48 (0.881)	2.76 (0.389)	-1.88 (0.208)	2.306 (0.134)	2.231 (0.153)	1.313 (0.545)	1.359 (0.523)	-1.6 (0.467)	-2.051 (0.35)	-2.48 (0.264)	-1.176 (0.604)
**PDQ-39**	0.52 (0.926)	3.04 (0.585)	-0.36 (0.892)	7.509 (0.006)	9.239 (0.001)	-1.483 (0.7)	2.94 (0.436)	-5.534 (0.157)	-5.081 (0.192)	-7.628 (0.055)	-1.738 (0.668)
**MOCA**	-0.2 (0.689)	0.04 (0.936)	-0.4 (0.218)	-0.884 (0.009)	-0.225 (0.507)	0.609 (0.197)	-0.641 (0.167)	0.73 (0.128)	-0.658 (0.168)	0.297 (0.537)	-0.42 (0.394)
**Time Up & GO**	-0.4 (0.799)	0.36 (0.819)	0.468 (0.626)	2.665 (0.008)	2.426 (0.015)	0.041 (0.977)	0.704 (0.608)	-1.061 (0.451)	-0.019 (0.989)	-1.003 (0.476)	0.131 (0.927)

B)

ANOVA test for group differences	P-value			FDR : Multiple Testing		
**Endpoints**	**Placebo vs 150mg vs 300mg nilotinib**	**Month**	**Placebo vs 150mg nilotinib vs 300mg nilotinib/Month**	**Placebo vs 150mg nilotinib vs 300mg nilotinib**	**Month**	**Placebo vs 150mg nilotinib vs 300mg nilotinib/Month**
**MDS-UPDRS I**	0.8766	0.255	0.8419	0.9717	0.2833	0.9355
**MDS-UPDRS II**	0.8013	0	0.424	0.9717	0	0.7221
**MDS-UPDRS III**	0.2649	0.0191	0.6449	0.9717	0.0319	0.8062
**MDS-UPDRS IV**	0.9717	0.7945	0.4333	0.9717	0.7945	0.7221
**MDS-UPDRS I-III**	0.4743	0.0652	0.4153	0.9717	0.0886	0.7221
**MDS-UPDRS I-IV**	0.5002	0.0708	0.5992	0.9717	0.0886	0.8062
**PDQ-39**	0.6379	3.00E-04	0.2228	0.9717	7.00E-04	0.7221
**MoCA**	0.4016	2.00E-04	0.1102	0.9717	7.00E-04	0.7221
**Time Up & GO**	0.6016	1.00E-04	0.959	0.9717	6.00E-04	0.959

eTable 8. Demographics showing participants who received dopamine agonists (DA) alone versus participants who received DA+ levodopa. UN: Unknown. Please note that Nil-PD-056 and Nil-PD-028 received amantadine only + emergency rescue Mirapex when needed

Placebo (year of diagnosis)	DA	Levodopa	150 mg nilotinib (year of diagnosis)	DA	Levodopa	300 mg nilotinib (year of diagnosis)	DA	Levodopa
**Nil-PD-001 (2012)**		+	**Nil-PD-002 (2001)**		+	**Nil-PD-003 (2014)**		+
**Nil-PD-006 (2009)**		+	**Nil-PD-004 (2003)**	+	+	**Nil-PD-005 (2009)**		+
**Nil-PD-007 (2008)**		+	**Nil-PD-011 (UN)**	+	+	**Nil-PD-008 (2008)**		+
**Nil-PD-009 (UN)**		+	**Nil-PD-012 (1996)**	+	+	**Nil-PD-015 (2006)**	+	+
**Nil-PD-010 (2007)**		+	**Nil-PD-014 (2006)**		+	**Nil-PD-016 (2011)**	+	NONE
**Nil-PD-013 (2012)**		+	**Nil-PD-018 (UN)**		+	**Nil-PD-017 (2015)**		+
**Nil-PD-021 (2004)**	+	+	**Nil-PD-019 (UN)**		+	**Nil-PD-022 (2015)**		+
**Nil-PD-024 (2008)**		+	**Nil-PD-020 (2005)**		+	**Nil-PD-023 (2012)**		+
**Nil-PD-025 (2010)**	+	+	**Nil-PD-026 (2008)**		+	**Nil-PD-027 (2008)**		+
**Nil-PD-032 (2009)**		+	**Nil-PD-028 (2015)**	+	NONE	**Nil-PD-029 (2013)**		+
**Nil-PD-034 (2009)**		+	**Nil-PD-033 (2005)**		+	**Nil-PD-030 (1989)**		+
**Nil-PD-035 (2013)**		+	**Nil-PD-036 (2014)**		+	**Nil-PD-031 (2011)**		+
**Nil-PD-037 (2016)**	+	NONE	**Nil-PD-038 (2008)**		+	**Nil-PD-040 (2005)**		+
**Nil-PD-042 (2000)**		+	**Nil-PD-039 (2009)**		+	**Nil-PD-043 (2016)**		+
**Nil-PD-046 (2000)**		+	**Nil-PD-041 (2013)**	+	NONE	**Nil-PD-045 (2012)**		+
**Nil-PD-047 (2011)**		+	**Nil-PD-044 (2003)**		+	**Nil-PD-048 (2016)**		+
**Nil-PD-051 (2016)**		+	**Nil-PD-049 (2001)**		+	**Nil-PD-052 (2013)**		+
**Nil-PD-053 (1996)**	+	+	**Nil-PD-050 (2005)**		+	**Nil-PD-054 (2006)**		+
**Nil-PD-056 (2009)**	+	NONE	**Nil-PD-055 (2004)**		+	**Nil-PD-058 (2011)**	+	+
**Nil-PD-057 (2008)**		+	**Nil-PD-060 (2013)**	+	+	**Nil-PD-062 (2007)**		+
**Nil-PD-059 (2011)**		+	**Nil-PD-061 (2008)**		+	**Nil-PD-064 (2006)**		+
**Nil-PD-066 (2008)**		+	**Nil-PD-063 (2007)**		+	**Nil-PD-065 (1994)**		+
**Nil-PD-067 (2017)**	+	NONE	**Nil-PD-069 (2004)**	+	+	**Nil-PD-068 (2012)**	+	+
**Nil-PD-071 (2014)**		+	**Nil-PD-072 (2014)**		+	**Nil-PD-070 (2015)**		+
**Nil-PD-074 (UN)**	+	+	**Nil-PD-073 (UN)**		+	**Nil-PD-075 (2002)**		+

eTable 9. Concentration of nilotinib in CSF and plasma

**Nilotinib Concentration**	**Placebo**	**Nilotinib 150 mg**	**Noncompartmental**			**Nilotinib 300 mg**	**Noncompartmental**		
	**Mean ± SD**	**Mean ± SD**	**Tmax**	**Cmax (nM)**	**AUC (ng/ml*h)**	**Mean ± SD**	**Tmax**	**Cmax (nM)**	**AUC (ng/ml*h)**
**CSF (nM)**	**0.0 ± 0**	**0.9343 ± 0.5010**	**2**	**1.85**	**6.157**	**1.613 ± 1.280**	**4**	**4.12**	**9.56**
**Plasma (nM)**	**0.0 ± 0**	**245.2 ± 105.2**	**4**	**546**	**1612**	**299.5 ± 147.4**	**3**	**736**	**1930**
**Ratio of CSF/Plasma (%)**	**0.0 (0%)**	**0.0037 (0.39%)**				**0.005 (0.54%)**			
