# Supplementary material for: Nilotinib Effects on Safety, Tolerability, and Potential Biomarkers in Parkinson Disease: A Phase 2 Randomized Clinical Trial
Source: JAMA Neurol. 2019 Dec 16;77(3):309–17. doi: 10.1001/jamaneurol.2019.4200 (PMC6990742; doi:10.1001/jamaneurol.2019.4200)
Supplement: Supplement 3. — Data Sharing Statement [file jamaneurol-77-309-s003.pdf]

Data Sharing Statement

Pagan. Nilotinib Effects on Safety, Tolerability, and Potential Biomarkers in Parkinson Disease. *JAMA Neurol*. Published December 16, 2019. 10.1001/jamaneurol.2019.4200

Data
Data available: Yes
Data types: Deidentified participant data
How to access data: cem46@georgetown.edu
When available: With publication

Supporting Documents
Document types: None

Additional Information
Who can access the data: anyone requesting data
Types of analyses: as in publication
Mechanisms of data availability: as in publication or emial
Any additional restrictions: None
